# Supplementary material for: Heparan sulfate regulates amphiregulin programming of tissue reparative lung mesenchymal cells during influenza A virus infection in mice
Source: Nat Commun. 2025 Mar 3;16:2129. doi: 10.1038/s41467-025-57362-z (PMC11876457; doi:10.1038/s41467-025-57362-z)
Supplement: Supplementary file 1 — Supplementary Information [file 41467_2025_57362_MOESM1_ESM.pdf]

Supplementary Information for:

# Heparan sulfate regulates amphiregulin programming of tissue reparative lung mesenchymal cells during influenza A virus infection in mice

Lucas F. Loffredo, Anmol Kustagi, Olivia R. Ringham, Fangda Li, Kenia de los Santos-Alexis, Anjali Saqi, Nicholas Arpaia

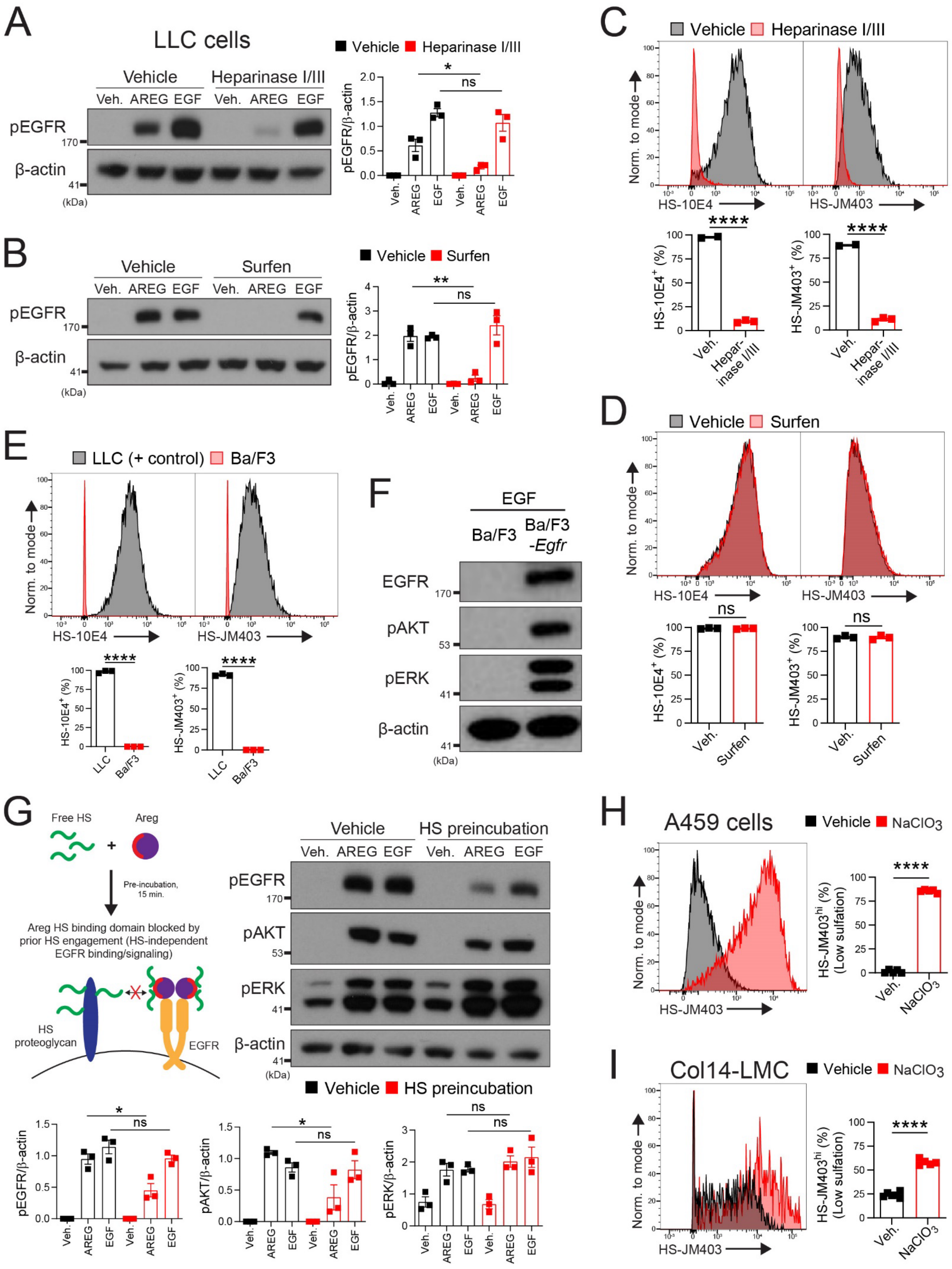
A
LLC cells
Vehicle
Heparinase I/III
Veh.
AREG
EGF
pEGFR
170
β-actin
41
(kDa)
pEGFR/β-actin
*
ns
B
Vehicle
Surfen
**
C
Norm. to mode
HS-10E4
HS-JM403
HS-10E4+ (%)
HS-JM403+ (%)
****
Heparinase I/III
D
ns
E
LLC (+ control)
Ba/F3
LLC
F
EGF
Ba/F3
Ba/F3 -Egfr
EGFR
pAKT
53
pERK
G
Free HS
Areg
Pre-incubation, 15 min.
Areg HS binding domain blocked by prior HS engagement (HS-independent EGFR binding/signaling)
HS proteoglycan
EGFR
HS preincubation
pAKT/β-actin
pERK/β-actin
H
A459 cells
NaClO3
HS-JM403hi (%) (Low sulfation)
I
Col14-LMC

**Supplementary Figure 1. Areg signaling is altered but not abrogated in the context of HS inhibition – additional data (Supplementary data for Figure 1)**

**(A-B)** Western blotting for phospho-EGFR (Y1068) and β-actin of vehicle or heparinase I/III-treated (1h) LLC cells (A), or vehicle or surfen-treated (15 min.) LLC cells (B), stimulated (15 min.) with vehicle, mouse AREG (500 ng/ml), or mouse EGF (100 ng/ml). Representative western blots shown. n=3 per condition, graph contains all values from 3 experiments. **(C-D)** Flow cytometry using HS-directed antibodies 10E4 and JM403 on LLC cells treated with vehicle (n=2) or heparinase I/III (n=3) (1h) (C), or on LLC cells treated with vehicle (n=3) or surfen (n=3) (15 min.) (D). Representative flow cytometry plots shown. Gating based on FMO controls. Graphs contain all values from 2 experiments. **(E)** Flow cytometry using HS-directed antibodies 10E4 or JM403 on untreated Ba/F3 cells, with untreated LLC cells used as a positive staining control. Representative flow cytometry plots shown. Gating based on FMO controls. n=3 per cell type, graphs contain all values from 2 experiments. **(F)** Western blot showing transfection of Ba/F3 cells with mouse *Egfr* (EGFR blot), and EGF (50 ng/ml)-inducible downstream signaling via phospho-AKT (S473) and phospho-ERK (T202/Y204) (β-actin also included). Representative western blots shown from 4 experiments. **(G)** Western blotting for phospho-EGFR (Y1068), phospho-AKT (S473), phospho-ERK (T202/Y204), and β-actin of vehicle- or HS-pretreated (15 min.) vehicle, mouse AREG (500 ng/ml), or mouse EGF (100 ng/ml), subsequently applied to LLC cells (15 min.). Representative western blots shown. n=3 per condition, graphs contain all values from 3 experiments. Diagram included of experimental setup (top left). **(H)** Flow cytometry using HS-directed antibody JM403 on A549 cells treated with vehicle or $NaClO_3$ (16-18h). Representative flow cytometry plot shown. Gating based on FMO control. n=4 per condition, graph contains all values from 2 experiments. **(I)** Flow cytometry using HS-directed antibody JM403 on Col14-LMC treated with vehicle or $NaClO_3$ (16-18h). Representative flow cytometry plot shown. Gating based on FMO control. n=5 per condition, graph contains all values from 2 experiments. Statistical analysis done for western blot/flow cytometry data using two-tailed unpaired t tests. Mean and standard error displayed on graphs; n.s: not significant, *:0.01<p<0.05, **:0.001<p<0.01, ***:0.0001<p<0.001, ****:p<0.0001. Source data are provided as a Source Data file.

## Gating strategy for LMC subsets from total lung

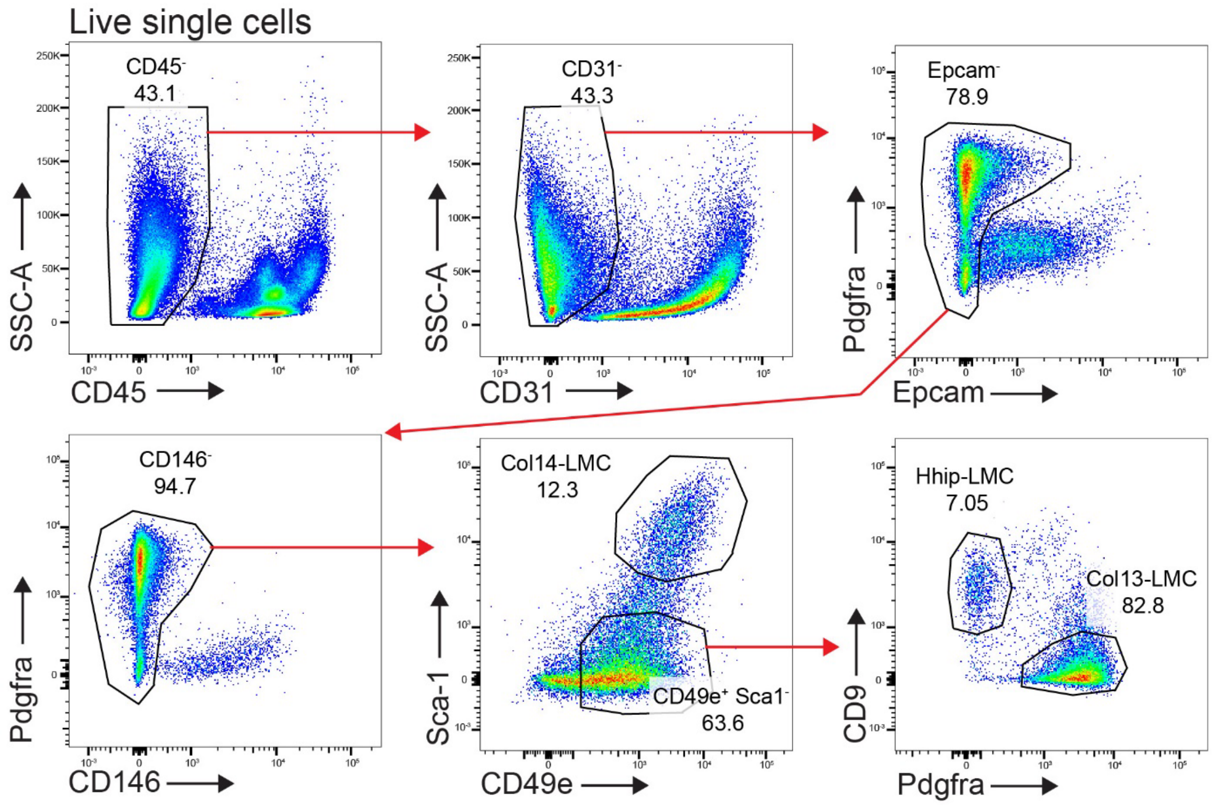


## Gating strategy for bulk LMC culture or Col14-LMC sorting from negative bead enriched lung (CD45, CD31, Epcam, Ter-119)

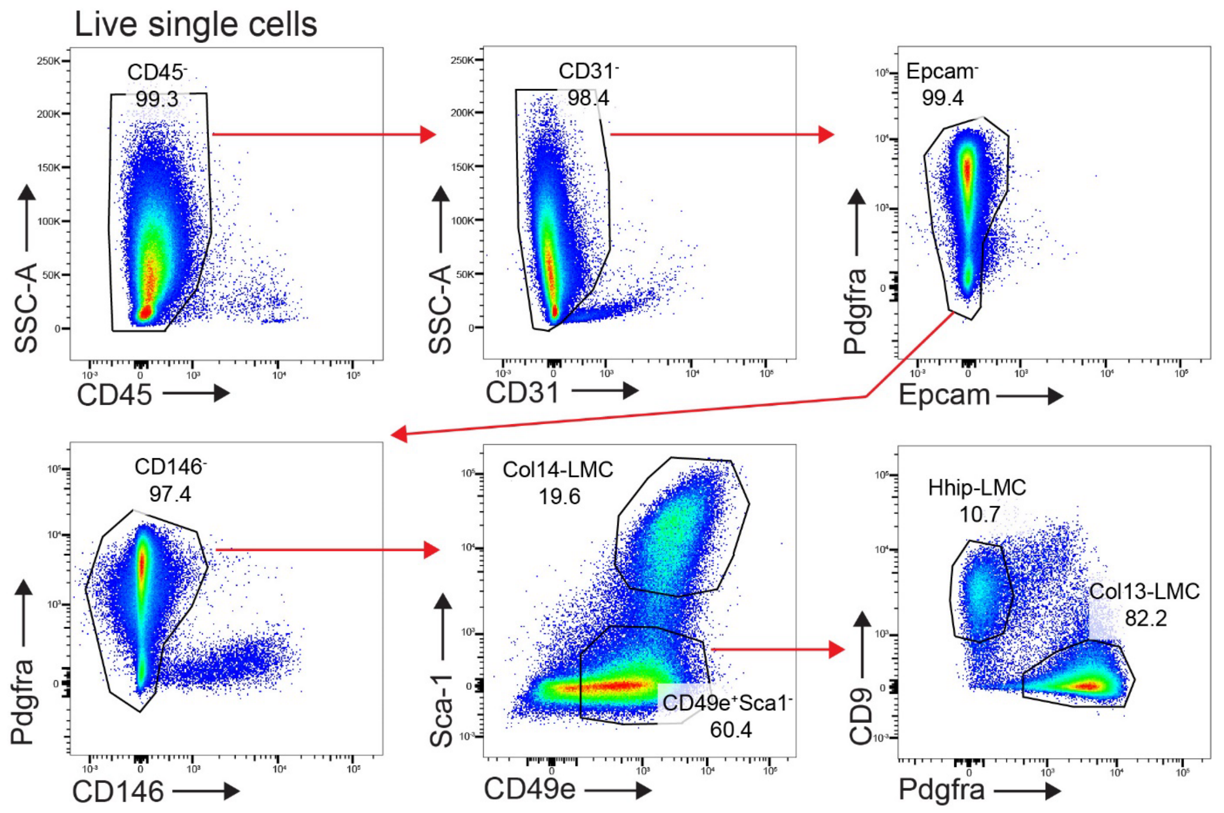

**Supplementary Figure 2. Flow cytometry gating strategies for mouse LMC**

Flow cytometry gating strategy for staining of LMC subsets from baseline WT mouse lung, and from negative bead enriched baseline WT mouse lung. Negative bead enrichment was done with biotinylated antibodies towards Ter-119, CD45, CD31, and Epcam, to remove red blood cells, hematopoietic cells, endothelial cells, and epithelial cells, respectively. Flow cytometry analyses of different LMC populations were done using the panel from total lung (no enrichment), while bulk LMC (cultured directly or cell sorting of Col14-LMC) for experiments was done from the negative bead-enriched lung panel. Percent of previously gated population displayed in plots.

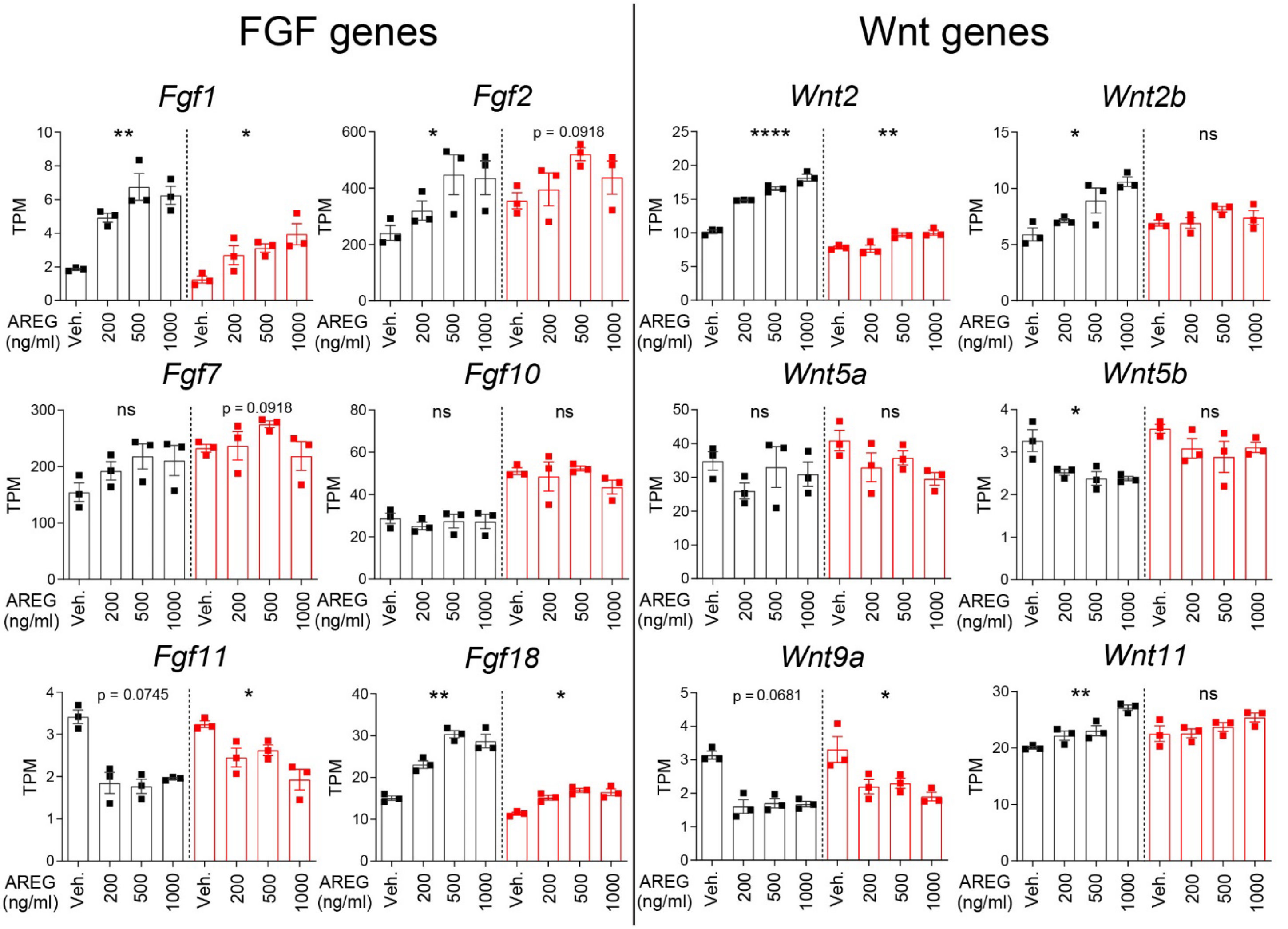


**Supplementary Figure 3. HS dependency of Areg-induced FGF and Wnt gene expression in Col14-LMC (Supplementary data for Figure 2)**

Transcript per million (TPM) values from RNA-seq for all FGF genes and Wnt genes with average TPM>2 (n=3 per group). Statistical analysis done for post-hoc RNA-seq data of individual genes across four groups using Kruskal-Wallis tests. Mean and standard error displayed on graphs; n.s: not significant, *:0.01<p<0.05, **:0.001<p<0.01, ***:0.0001<p<0.001, ****:p<0.0001. Source data are provided as a Source Data file.

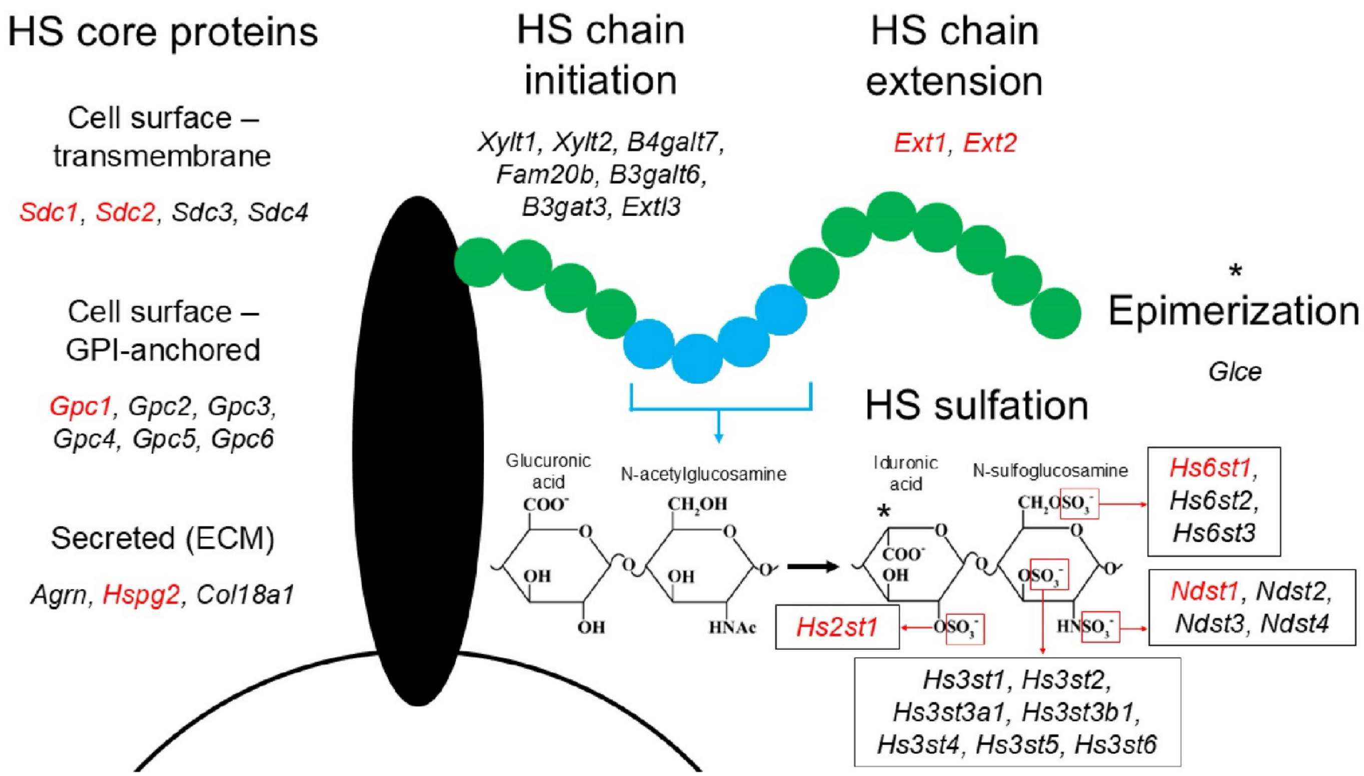


**Supplementary Figure 4. Summary of HS-related genes and their functions**

Schematic of HS-synthesis related genes, with those included in LLC cell KO panel in red. HS must be attached to core proteins at specific serine sites along their protein structure, either cell surface proteins – syndecans (transmembrane) or glypicans (GPI-anchored) – or secreted ECM proteins. While these core proteins are trafficked through the Golgi apparatus, a series of enzymes catalyzes the addition of the specific sugar moiety combination that initiates all HS chains. Then, the critical enzymes Ext1 and Ext2 are nonredundantly responsible for adding the repeated sugar subunit of glucuronic acid and N-acetylglucosamine to the HS chain. The HS chain upon formation lacks sulfation; a series of enzymes, also in the Golgi apparatus, operate on disparate regions of the HS chain to add sulfation groups, the negative charge of which largely confer signaling alteration potential to HS towards positively charge HS binding protein domains. These enzymes are thought to operate in sequential fashion (i.e., sulfation must occur at one site before it can occur at the next), in this order: the N-sulfotransferase enzyme group (Ndst1-4) exchanges an acetyl group for a sulfate group in N-acetylglucosamine (creating N-sulfoglucosamine); Glce epimerizes the indicated carbon in glucuronic acid (creating iduronic acid); the 2-O-sulfotransferase enzyme (Hs2st1) adds a sulfate group at the indicated hydroxyl group in iduronic acid; the 6-O-sulfotransferase enzyme group (Hs6st1-3) adds a sulfate group at the indicated hydroxyl group in N-sulfoglucosamine; and the 3-O-sulfotransferase enzyme group (Hs3st1-6) adds a sulfate group at the indicated hydroxyl group in iduronic acid. HS contains significant heterogeneity in their sulfation profile along each individual chain, with alternating regions of little or no sulfation and regions with high sulfation.

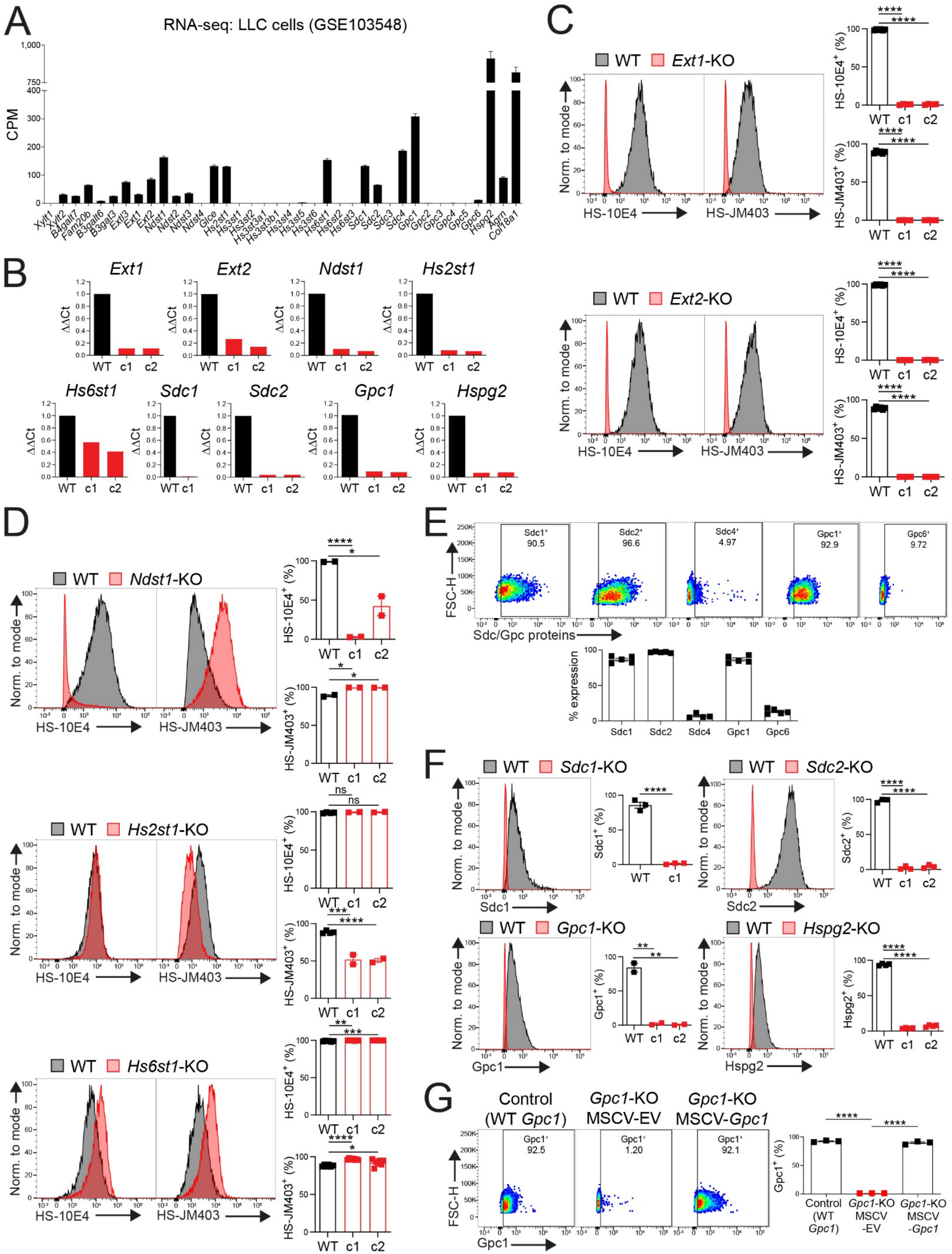
A
RNA-seq: LLC cells (GSE103548)
CPM
Xylt1 Xylt2 B4galt7 Fam20b B3galt6 B3gat3 Extl3 Ext1 Ext2 Ndst1 Ndst2 Ndst3 Ndst4 Glce Hs2st1 Hs3st1 Hs3st2 Hs3st3a1 Hs3st3b1 Hs3st4 Hs3st5 Hs3st6 Hs6st1 Hs6st2 Hs6st3 Sdc1 Sdc2 Sdc3 Sdc4 Gpc1 Gpc2 Gpc3 Gpc4 Gpc5 Gpc6 Hspg2 Agrn Col18a1
B
Ext1
Ext2
Ndst1
Hs2st1
Hs6st1
Sdc1
Sdc2
Gpc1
Hspg2
ΔΔCt
WT c1 c2
C
WT Ext1-KO
WT Ext2-KO
Norm. to mode
HS-10E4
HS-JM403
HS-10E4+ (%)
HS-JM403+ (%)
D
WT Ndst1-KO
WT Hs2st1-KO
WT Hs6st1-KO
E
Sdc1+ 90.5
Sdc2+ 96.6
Sdc4+ 4.97
Gpc1+ 92.9
Gpc6+ 9.72
FSC-H
Sdc/Gpc proteins
% expression
Sdc1 Sdc2 Sdc4 Gpc1 Gpc6
F
WT Sdc1-KO
WT Sdc2-KO
WT Gpc1-KO
WT Hspg2-KO
Sdc1+ (%)
Sdc2+ (%)
Gpc1+ (%)
Hspg2+ (%)
G
Control (WT Gpc1)
Gpc1-KO MSCV-EV
Gpc1-KO MSCV-Gpc1
Gpc1+ 92.5
Gpc1+ 1.20
Gpc1+ 92.1
Gpc1+ (%)

**Supplementary Figure 5. HS-related gene knockout cell lines identify glypican-1 as a critical HS core protein for Areg signaling – additional data (Supplementary data for Figure 3)**

**(A)** Data from public bulk RNA-seq of LLC cells (GSE103548), of HS synthesis-related genes. CPM: Counts per million. **(B)** qPCR on WT LLC cells or LLC cell KO sublines for genes targeted by CRISPR-Cas9 in LLC cell KO panel for HS-related genes. Expression values were computed as ΔΔCT values, compared to WT. c1/c2: clone 1/clone 2 (i.e., different single cell subcloned lines). Graphs contain all values from 1 experiment per subline. **(C-D)** Flow cytometry using HS-directed antibodies 10E4 and JM403 on WT (n=6) vs. *Ext1*-KO (n=4 per clone) or *Ext2*-KO (n=4 per clone) LLC cell sublines (C), or on WT (n=2 for *Ndst1*-KO controls, n=4 for *Hs2st1*-KO controls, and n=6 for *Hs6st1*-KO controls) vs. *Ndst1*-KO (n=2 per clone), *Hs2st1*-KO (n=2 per clone), or *Hs6st1*-KO (n=7 per clone) LLC cell sublines (D). Representative flow cytometry plots shown. Gating based on FMO controls. Graphs contain all values from 2-6 experiments. **(E)** Flow cytometry using antibodies targeting Sdc1 (n=5), Sdc2 (n=5), Sdc4 (n=4), Gpc1 (n=5), or Gpc6 (n=5) on WT LLC cells. Representative flow cytometry plots shown. Gating based on IgG controls. Percent staining positive displayed in plots. Graph contains all values from 2-4 experiments. **(F)** Flow cytometry using antibodies targeting Sdc1, Sdc2, or Gpc1 on WT (n=3 for *Sdc1*-KO controls and *Sdc2*-KO controls, n=2 for *Gpc1*-KO controls, and n=4 for *Hspg2*-KO controls) vs. *Sdc1*-KO (n=3), *Sdc2*-KO (n=3 per clone), *Gpc1*-KO (n=2 per clone), or *Hspg2*-KO (n=4 per clone) LLC cell sublines. Representative flow cytometry plots shown. Gating based on FMO controls. Graphs contain all values from 2-3 experiments. **(G)** Flow cytometry using Gpc1-targeted antibody on CRISPR-Cas9 control (WT *Gpc1*), *Gpc1*-KO LLC cells transduced with empty vector MSCV retrovirus (MSCV-EV), or *Gpc1*-KO LLC cells transduced with MSCV retrovirus with *Gpc1* mRNA (MSCV-*Gpc1*). Representative flow cytometry plots shown. Gating based on FMO control. Percent staining positive displayed in plots. n=3 per group, graph contains all values from 3 experiments. Statistical analysis done for flow cytometry data using two-tailed unpaired t tests. Mean and standard error displayed on graphs; n.s: not significant, *:0.01<p<0.05, **:0.001<p<0.01, ***:0.0001<p<0.001, ****:p<0.0001. Source data are provided as a Source Data file.

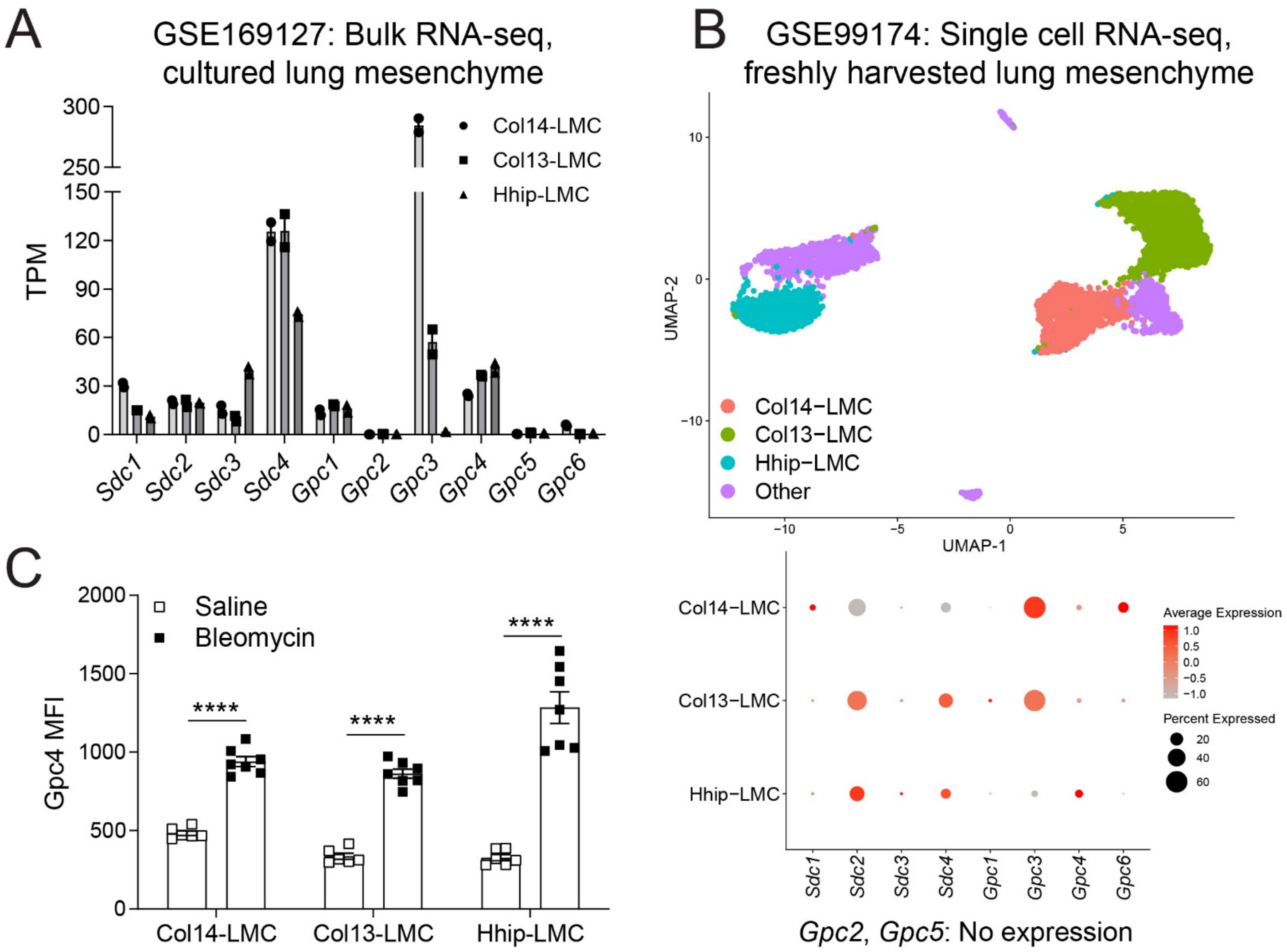


**Supplementary Figure 6. Glypican-4 is critical for proper Areg signaling in primary Areg-responsive cells and is upregulated on Col14-LMC in the context of viral lung infection – additional data (Supplementary data for Figure 4)**

**(A)** Data from public bulk RNA-seq of lung mesenchyme subsets (GSE169127), for HS core protein genes. TPM: transcripts per million. **(B)** Data from public scRNA-seq of lung mesenchyme (GSE99714), for HS core protein genes. **(C)** Gpc4 protein expression determined by flow cytometry (MFI: median fluorescence intensity), using a Gpc4-directed antibody, on freshly harvested LMC subsets, from either saline-treated (n=6) or bleomycin-treated (n=7) (1 U/kg) lungs (14 d.p.i). Gating strategy shown in Supplementary Fig. 2 (total lung). Graph contains all values from 2 experiments. Statistical analysis done for flow cytometry data using two-tailed unpaired t tests. Mean and standard error displayed on graphs; n.s: not significant, *:0.01<p<0.05, **:0.001<p<0.01, ***:0.0001<p<0.001, ****:p<0.0001. Source data are provided as a Source Data file.

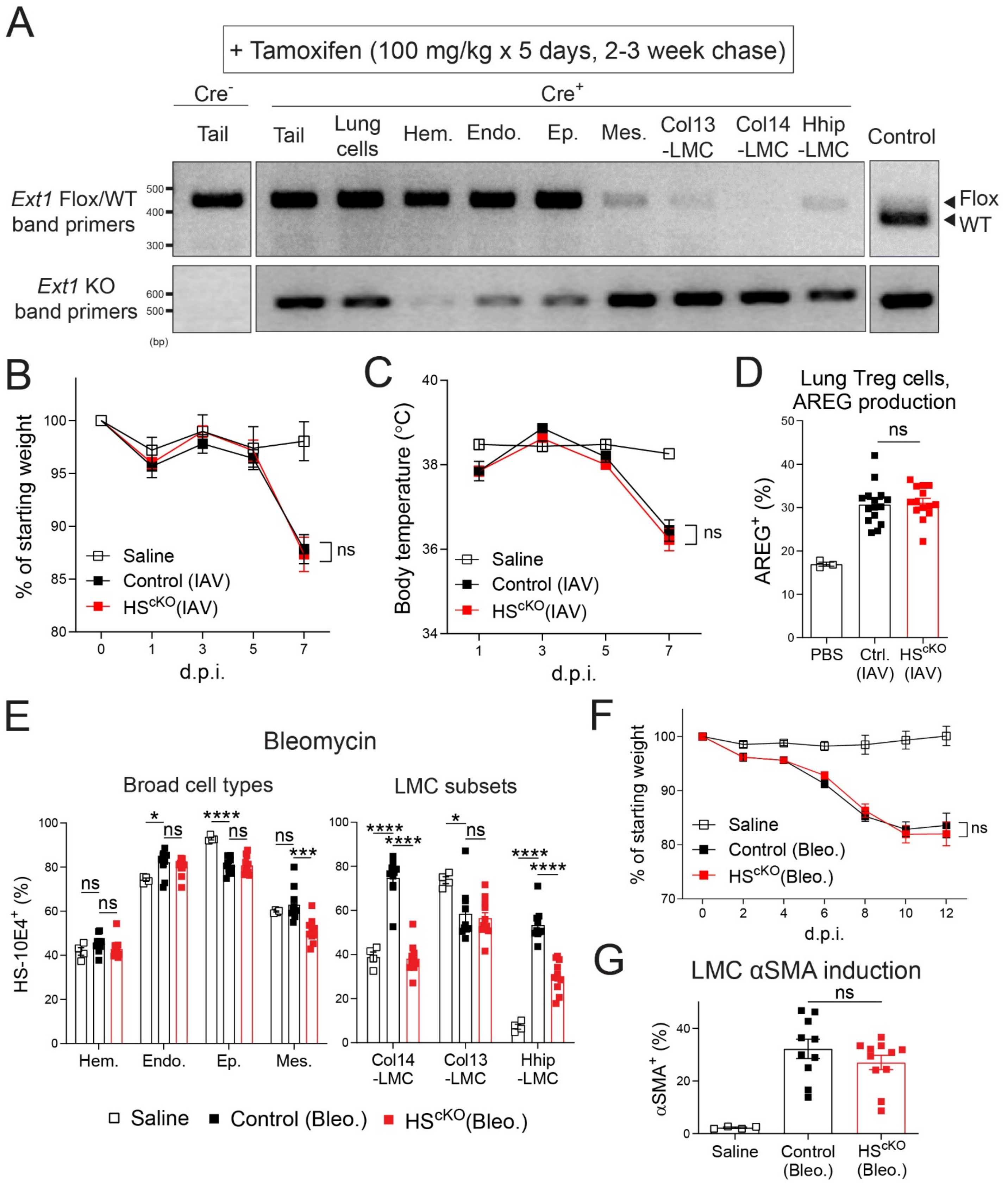
A
+ Tamoxifen (100 mg/kg x 5 days, 2-3 week chase)
Cre-
Cre+
Tail
Tail
Lung cells
Hem.
Endo.
Ep.
Mes.
Col13 -LMC
Col14 -LMC
Hhip -LMC
Control
Ext1 Flox/WT band primers
Ext1 KO band primers
Flox
WT
(bp)
B
% of starting weight
Saline
Control (IAV)
HScKO(IAV)
ns
d.p.i.
C
Body temperature (°C)
Saline
Control (IAV)
HScKO(IAV)
ns
d.p.i.
D
Lung Treg cells, AREG production
AREG+ (%)
ns
PBS
Ctrl. (IAV)
HScKO (IAV)
E
Bleomycin
Broad cell types
LMC subsets
HS-10E4+ (%)
Hem.
Endo.
Ep.
Mes.
Col14 -LMC
Col13 -LMC
Hhip -LMC
Saline
Control (Bleo.)
HScKO(Bleo.)
F
% of starting weight
Saline
Control (Bleo.)
HScKO(Bleo.)
ns
d.p.i.
G
LMC αSMA induction
αSMA+ (%)
ns
Saline
Control (Bleo.)
HScKO (Bleo.)

**Supplementary Figure 7. HS affects Areg-related tissue repair pathways in vivo – additional data (Supplementary data for Figure 5)**

**(A)** PCR-based genotyping of DNA from tails, bulk lung cells, and flow cytometry-sorted cell populations from TMX-administered control and $HS^{cKO}$ mice at baseline. Top gel: PCR with primers targeting WT mouse *Ext1* (WT band) (389 bp), or *Ext1* with LoxP sites (Flox band) (460 bp). Bottom gel: PCR with primers targeting LoxP recombination-mediated, exon 1-excised *Ext1* (KO band) (509 bp). Hem.: hematopoietic, Endo.: endothelial, Ep.: epithelial, Mes.: mesenchymal. Representative gel shown from 2 separate experiments. **(B-C)** Percent starting weight (B) and body temperature (C) for mock-infection control (PBS) mice (n=5), IAV-infected (100 TCID50) control mice (n=15), and IAV-infected $HS^{cKO}$ mice (n=14) (all TMX-treated) at indicated timepoints. Graph contains all values from 3 experiments. **(D)** AREG production by lung Treg cells from mice in (B-C) at 8 d.p.i., assessed by flow cytometry. Gating strategy for Treg cells in Supplementary Fig. 8. Gating based on FMO control. Graph contains all values from 3 experiments. **(E)** HS presence, assessed by flow cytometric staining for the HS-directed 10E4 antibody, on indicated cell populations from mock-treated control (saline) mice (n=4), bleomycin-treated (1U/kg) control mice (n=11), and bleomycin-treated $HS^{cKO}$ mice (n=12) (all TMX-treated) at 14 d.p.i.. Gating strategy in Supplementary Fig. 2 (total lung). Gating based on FMO control. Graphs contain all values from 3 experiments. **(F)** Percent starting weight for mock-treated control (saline) mice (n=4), bleomycin-treated (1U/kg) control mice (n=11), and bleomycin-treated $HS^{cKO}$ mice (n=12) (all TMX-treated) at indicated timepoints. Graph contains all values from 3 experiments. **(G)** Fibrosis induction, assessed via flow cytometry using a α-smooth muscle actin (αSMA)-directed antibody, in $Pdgfra^+$ LMC from mice in (F), at 14 d.p.i.. Gating strategy in Supplementary Fig. 2 (total lung). Gating based on FMO control. Graph contains all values from 3 experiments. Statistical analysis done for weight/body temperature data across all timepoints using two-way repeated measures ANOVA, and flow cytometry using two-tailed unpaired t tests. Mean and standard error displayed on graphs; n.s: not significant, *:$0.01<p<0.05$, **:$0.001<p<0.01$, ***:$0.0001<p<0.001$, ****:$p<0.0001$. Source data are provided as a Source Data file.

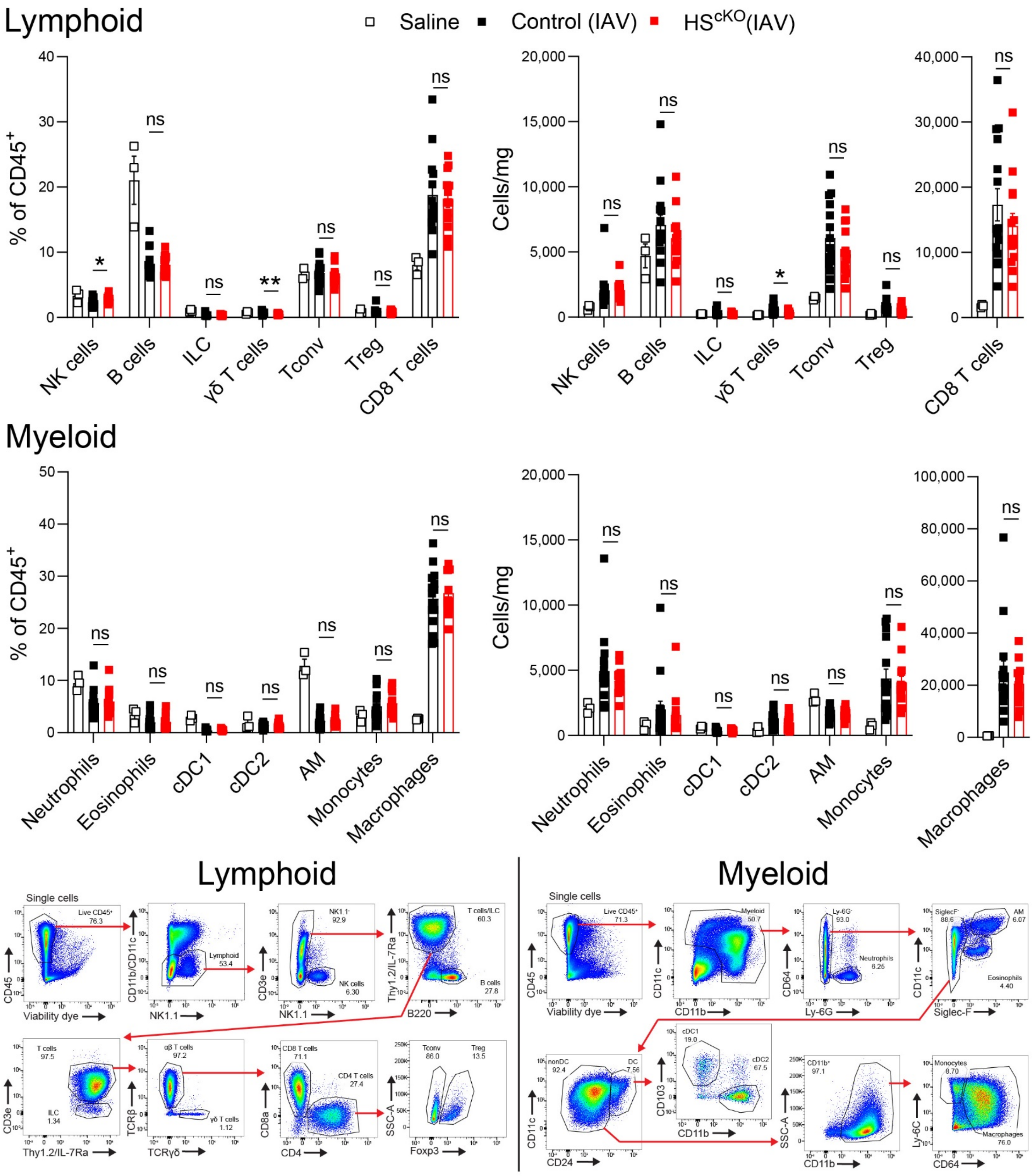

Lymphoid
Saline
Control (IAV)
HScKO(IAV)
% of CD45+
Cells/mg
ns
*
**
NK cells
B cells
ILC
γδ T cells
Tconv
Treg
CD8 T cells
Myeloid
Neutrophils
Eosinophils
cDC1
cDC2
AM
Monocytes
Macrophages
Lymphoid
Single cells
Live CD45+
76.3
Viability dye
CD45
CD11b/CD11c
NK1.1
Lymphoid
53.4
NK1.1-
92.9
CD3e
NK cells
6.30
T cells/ILC
60.3
Thy1.2/IL-7Ra
B220
B cells
27.8
T cells
97.5
ILC
1.34
Thy1.2/IL-7Ra
αβ T cells
97.2
γδ T cells
1.12
TCRβ
TCRγδ
CD8 T cells
71.1
CD4 T cells
27.4
CD8a
CD4
Tconv
86.0
Treg
13.5
SSC-A
Foxp3
Myeloid
Single cells
Live CD45+
71.3
Viability dye
Myeloid
50.7
CD11c
CD11b
Ly-6G-
93.0
Neutrophils
6.25
CD64
Ly-6G
SiglecF-
88.6
AM
6.07
Eosinophils
4.40
Siglec-F
nonDC
92.4
DC
7.56
CD11c
CD24
cDC1
19.0
cDC2
67.5
CD103
CD11b
CD11b+
97.1
SSC-A
CD11b
Monocytes
8.70
Macrophages
76.0
Ly-6C
CD64

**Supplementary Figure 8. Immune infiltrate in IAV-infected control and HS$^{cKO}$ mouse lungs (Supplementary data for Figure 5)**

Percentage of all CD45$^{+}$ cells and cell counts per lung weight (determined by flow cytometry) for predominant myeloid and lymphoid immune cell types from mock-infection control (PBS) mice (n=3), IAV-infected (100 TCID50) control mice (n=15), and IAV-infected HS$^{cKO}$ mice (n=14) (all TMX-treated) at 8 d.p.i.. ILC: Innate lymphoid cells, cDC1/2: conventional dendritic cells type 1/2, AM: alveolar macrophages. Gating strategies for immune cell type identification shown (below); example plots are from IAV-infected mouse lungs (8 d.p.i.). Percent of previously gated population displayed in plots. Graphs contain all values from 3 experiments. Statistical analysis done for flow cytometry data using two-tailed unpaired t tests. Mean and standard error displayed on graphs; n.s: not significant, *:0.01<p<0.05, **:0.001<p<0.01, ***:0.0001<p<0.001, ****:p<0.0001. Source data are provided as a Source Data file.

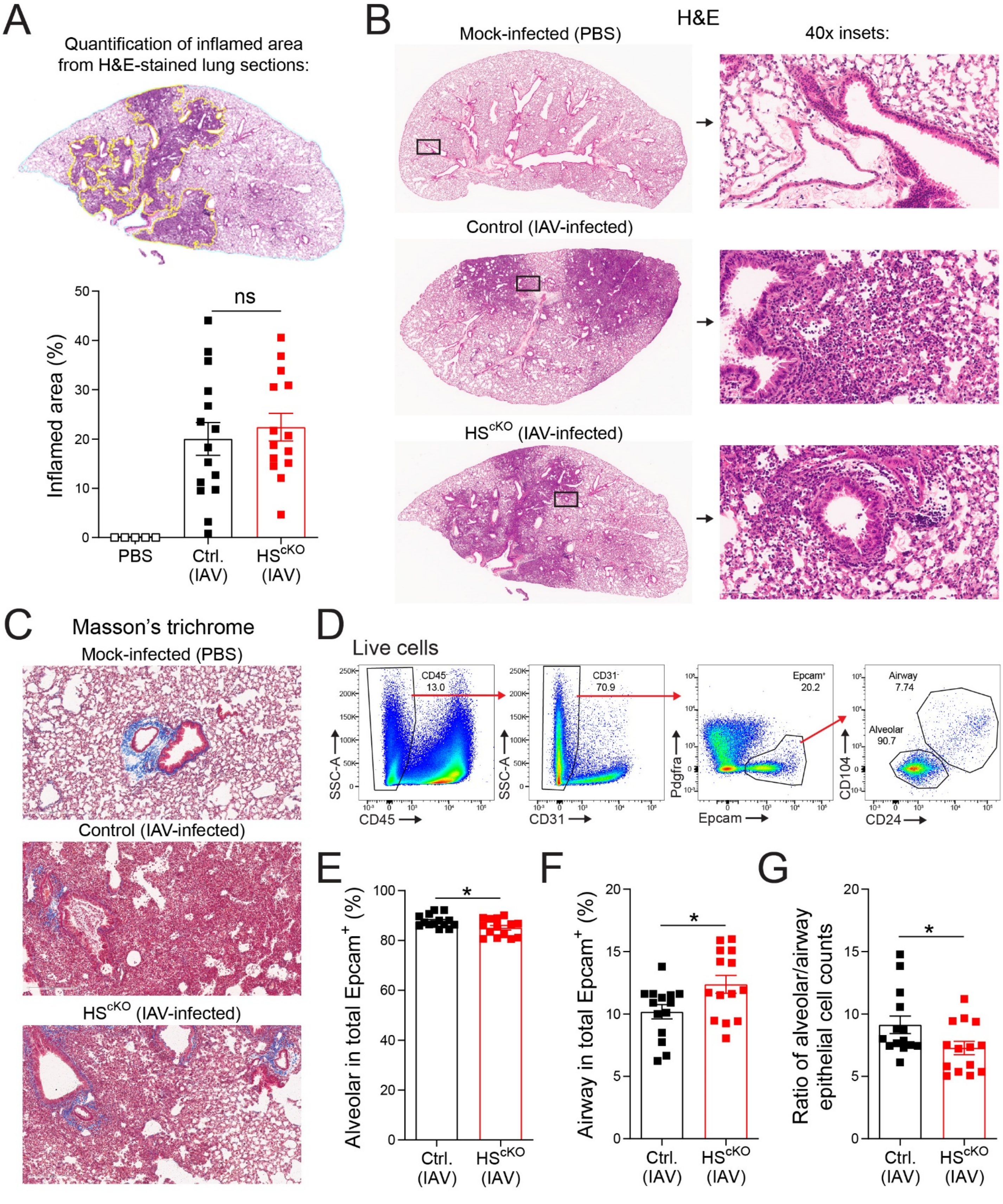

A
Quantification of inflamed area from H&E-stained lung sections:
ns
Inflamed area (%)
50
40
30
20
10
0
PBS
Ctrl. (IAV)
HScKO (IAV)
B
H&E
Mock-infected (PBS)
40x insets:
Control (IAV-infected)
HScKO (IAV-infected)
C
Masson's trichrome
Mock-infected (PBS)
Control (IAV-infected)
HScKO (IAV-infected)
D
Live cells
CD45- 13.0
CD31- 70.9
Epcam+ 20.2
Airway 7.74
Alveolar 90.7
SSC-A
CD45
CD31
Pdgfra
Epcam
CD104
CD24
E
Alveolar in total Epcam+ (%)
*
Ctrl. (IAV)
HScKO (IAV)
F
Airway in total Epcam+ (%)
*
Ctrl. (IAV)
HScKO (IAV)
G
Ratio of alveolar/airway epithelial cell counts
*
Ctrl. (IAV)
HScKO (IAV)

**Supplementary Figure 9. Histological analysis and epithelial flow cytometry analysis of IAV-infected control and $HS^{cKO}$ mouse lungs (Supplementary data for Figure 5)**

**(A)** Top: example of quantification of inflamed full mouse lung lobes, with yellow outlines highlighting inflamed regions following IAV infection (8 d.p.i.). Bottom: Percentage of inflamed area in mock-infected PBS-treated (n=5), control IAV-infected (n=15), and $HS^{cKO}$ IAV-infected mice (n=14). Graph contains all values from 3 experiments. **(B)** Full lobes of hematoxylin and eosin (H&E)-stained lungs from mock-infected PBS-treated, control IAV-infected, and $HS^{cKO}$ IAV-infected mice, with 40x zoomed insets highlighted with black boxes. Insets (right) show representative inflamed area from lungs of each treatment/genotype, to highlight airway and alveolar areas. **(C)** 40x zoomed-in sections on representative inflamed area from lungs of each treatment/genotype stained with Masson's Trichrome, to highlight lack of fibrosis in alveolar parenchyma (blue). **(D)** Gating strategy for analysis of airway vs. alveolar epithelial cells from lungs; example plots are from IAV-infected mouse lungs (8 d.p.i.). Percent of previously gated population displayed in plots. **(E-G)** Proportions of live alveolar epithelial cells (E), or airway cells (F) of total epithelial cells ($CD45^-CD31^-Epcam^+$) recovered from lung preparations for control IAV-infected (n=14) and $HS^{cKO}$ IAV-infected mice (n=14) (8 d.p.i.). Graph in (G) reflects ratio of alveolar epithelial cell counts to airway cell counts. Graphs contain all values from 3 experiments. Statistical analysis done for quantified immunofluorescence/flow cytometry data using two-tailed unpaired t tests. Mean and standard error displayed on graphs; n.s: not significant, *:$0.01<p<0.05$, **:$0.001<p<0.01$, ***:$0.0001<p<0.001$, ****:$p<0.0001$. Source data are provided as a Source Data file.

**Supplementary Methods**

*Western blotting*

For LLC cells and A549 cells, cells were plated at 250,000 cells per well in 6 well tissue culture treated plates, allowed to adhere for 24h, then washed and kept in starving media (all media components besides FBS) overnight (16-18h) before ligand treatment (15 min.) and lysis. HS-modifying agents used in certain experiments were sodium chlorate (3 mg/ml; added at time of starving media replacement, 16-18h prior to ligand treatment; Sigma-Aldrich), heparinase I/III (2-5 U/ml; added 1h prior to ligand treatment; Sigma-Aldrich), and surfen (5 μg/ml; added 15 min. prior to ligand treatment; EMD Millipore/Calbiochem). For pre-treatment experiments, prior to treatment of cells, ligands were pre-incubated with heparan sulfate (1 μg/ml; Sigma), heparin (1 μg/ml; Sigma), or desulfated heparin variants (2-O-desulfated heparin, 6-O-desulfated heparin, N-desulfated heparin, N-desulfated-reacetylated heparin [1 μg/ml; AMSBio]) for 15 min., then this mixture was applied to target cells. For Ba/F3 cells, cells were washed twice in starving media (all media components besides FBS; no rmIL-3), then plated at 2 million cells per well in 24 well non-tissue culture treated plates and cultured overnight (16-18h) before ligand treatment and lysis. For isolated bulk LMC or Col14-LMC (see "Bulk LMC or CD4 T cell negative bead enrichment, and Col14-LMC and Treg sorting"), cells were plated in mesenchymal cell media, allowed to adhere for 12-24h, then washed and kept in fresh mesenchymal cell media overnight (16-18h) or for 48h (for siRNA experiments) before ligand treatment and lysis. The next morning (LLC, Ba/F3, A549, Col14-LMC) or 48h later (bulk LMC treated with siRNA), cells were treated with vehicle, recombinant mouse Areg (R+D), human AREG (R+D), mouse EGF (Biolegend), or human EGF (R+D), for 15 min., then lysed for protein extraction. For LLC, A549, and Col14-LMC, cells were placed on ice, washed twice with cold 1x PBS to remove dead cells, then lysed by addition of TNT Lysis Buffer (20 mM Tris-HCl [Sigma-Aldrich], 150 mM sodium chloride [Fisher], 1 mM ethylenediaminetetraacetic acid [EDTA] disodium salt dihydrate [Fisher], 1 mM ethyleneglycol bis(2-aminoethyl ether)-N,N,N',N' tetraacetic acid [EGTA] [Gold Biotechnology], 1% Triton X-100 [Sigma-Aldrich], pH 8.0) with freshly added Halt Protease and Phosphatase Inhibitor Cocktail (Pierce) and

scraping of wells. For Ba/F3 cells (non-adherent), cells were harvested from wells, brought to 10 ml with cold 1x PBS, centrifuged at 550 x g, 4°C, 2 min., supernatant was aspirated, washed again in 10 ml cold 1x PBS, centrifuged/supernatant aspirated again, then lysed in TNT Lysis Buffer with freshly added inhibitors. Lysates were shaken for 30 min. on ice, then centrifuged for 15 min. at maximum speed; the supernatant was then removed and used for western blotting. Lysate supernatants were mixed with 6x SDS Sample Buffer (0.375 M Tris-HCl pH 6.8, 12% sodium dodecyl sulfate [SDS] [Millipore], 60% glycerol [Fisher], 0.6 M dithiothreitol [DTT] [Fisher], 0.06% bromophenol blue [Amresco]), incubated at 95°C for 5 min., cooled to room temperature, and loaded into Bolt 4–12% Bis-Tris Plus Gels (Thermo). SDS-PAGE (in Bolt MOPS SDS Running Buffer [Thermo]) and protein transfer (to Immobilon-P PVDF [Millipore] in Bolt Transfer Buffer [Thermo]) were done using the Mini Blot Module (Thermo). Membranes were washed with 1x TBS, blocked using 5% nonfat dry milk in TBST (1x TBS with 0.1% Tween-20 [Fisher]), washed 3 times with TBST, then stained with primary antibodies in 5% milk or BSA in TBST, overnight at 4°C (β-Actin [45 kDa; 1:1000; clone 8H10D10; Cell Signaling], Phospho-EGF Receptor Tyr1068 [175 kDa; 1:1000; clone D7A5; Cell Signaling], Phospho-Akt Receptor Ser473 [60 kDa; 1:2000; clone D9E; Cell Signaling], Phospho-p44/42 MAPK [Erk1/2] Thr202/Tyr204 [44/42 kDa; 1:2000; clone D13.14.4E; Cell Signaling], EGF Receptor [175 kDa; 1:1000; clone C74B9; Cell Signaling]). After overnight incubation, membranes were washed 3 times with TBST, then stained with secondary antibodies in 5% milk in TBST for 1h at room temperature (anti-mouse IgG HRP-linked antibody [1:5000; Cell Signaling], anti-rabbit IgG HRP-linked antibody [1:2500; Cell Signaling]). Afterwards, membranes were washed 3 times with TBST, then incubated with chemiluminescent substrate (Immobilon Classico Western HRP Substrate [Millipore], Immobilon Forte Western HRP Substrate [Millipore], SuperSignal West Femto Chemiluminescent Substrate [Thermo]). Membranes were then exposed to film, with films developed on a Kodak X-OMAT. Within each experiment, blots for phosphorylated proteins were performed first, with subsequent stripping with Restore Western Blot Stripping Buffer (Thermo) and reprobing for β-Actin as a loading control. Western blots were quantified using ImageJ.

*RNA extraction/qPCR*

RNA extraction was done using Trizol Reagent (Thermo) for lysis, followed by chloroform (Sigma-Aldrich)-based separation and precipitation of RNA with isopropanol (Fisher Scientific), sometimes using 5Prime Phase Lock Gel Heavy spin tubes (Quanta). RNA amounts were subsequently quantified using Nanodrop (Thermo), and cDNA was created using a qScript cDNA Synthesis Kit (Quanta, Catalog # 95048). qPCR was performed on cDNA using SYBR Green qPCR Master Mix (Thermo, Catalog # K0253) and a Bio-Rad CFX384 qPCR system. Primers were created for this study using Integrated DNA Technology's PrimerQuest platform, and University of California Santa Clara's In-Silico PCR platform; all primers were ordered from Integrated DNA Technology and are listed in Supplementary Table 1. Analysis was done by calculating ΔΔCt values relative to housekeeping gene (*Hprt*), or by calculating the copies of an analyzed gene per copies of housekeeping gene (*Hprt*) (see Methods section in main article).

*Lung processing*

Mice were euthanized and dissected to expose the lungs. Perfusion of the lungs was performed, after nicking the left femoral artery and the left atrium of the heart, through the left ventricle of the heart with 10 ml of cold 1x PBS. Bronchoalveolar lavage (BAL) was performed in the cases where tissue immune populations were analyzed or where bulk LMC was cultured directly after negative enrichment (this step was not done for sorting of Col14-LMC, sorting of Treg cells, or flow cytometric analysis of tissue mesenchymal cells). Lungs were extracted and placed in 0.5 ml tissue preparation media (RPMI with 100x penicillin/streptomycin, 100x GlutaMAX, 100x HEPES [all Gibco], and 5% FBS [Corning]) in a 5 ml Eppendorf tube, where they were minced. 3.5 ml was added to tubes of tissue preparation media with 5 U/ml DNAse, 1 mg/ml of collagenase A, and 1 mg/ml of dispase (with dispase pre-dissolved in 1x PBS consisting of 10% of the volume of the final digestion mixture). Lungs were digested in shaking incubator set to 110 r.p.m. at 37°C for 1h. Suspensions were then poured over a 100 µm cell strainer

(Corning) into a 50 ml Falcon tube (Corning), pushed through the mesh with the top of a syringe, rinsed with 10 ml tissue preparation media, pushed through again, then rinsed with 5 ml tissue preparation media. Cells were centrifuged at 450 x g/4°C/5 min., then supernatants were poured off (due to delicate nature of pellet resulting from use of dispase). Pellets were resuspended in 2 ml of 1x ACK lysis buffer (deionized water with 154 mM ammonium chloride [Fisher], 10 mM potassium bicarbonate [Fisher], 0.1 mM ethylenediaminetetraacetic acid [EDTA] disodium salt dihydrate [Fisher], pH 7.2) and incubated at room temperature for 2 min., then quenched with 10 ml tissue preparation media and ran through a 100 µm nylon mesh sheet into a 15 ml Falcon tube (Corning). Cells were centrifuged at 450 x g/4°C/5 min., the supernatant was aspirated, and cells were resuspended in 1 ml of tissue preparation media and placed on ice. Cells were then used for antibody staining for flow cytometry or negative bead enrichment and/or sorting.

*Bulk LMC or CD4 T cell negative bead enrichment, and Col14-LMC and Treg sorting*

Lungs were prepared as described in "Lung processing". Lung cells were then negatively enriched for mesenchymal cells or CD4 T cells using iMag Streptavidin Particles Plus – DM beads (BD, Catalog # 557812), according to their protocol, using a DynaMag-2 magnetic system (Invitrogen). For bulk LMC and Col14-LMC isolation, biotinylated antibodies towards mouse CD45 (1:200; clone 30-F11; Biolegend), CD31 (1:200; clone 390; Biolegend), Epcam (1:200; clone G8.8; Biolegend), and TER-119 (1:200; clone TER-119; Biolegend) were used. For CD4 T cell isolation, biotinylated antibodies towards mouse CD31 (1:200; clone 390; Biolegend), Epcam (1:200; clone G8.8; Biolegend), TER-119 (1:200; clone TER-119; Biolegend), Pdgfra (1:200; clone APA5; Biolegend), CD19 (1:200; clone 6D5; Biolegend), NK1.1 (1:200; clone PK136; Biolegend), CD11b (1:200; clone M1/70; Biolegend), CD11c (1:200; clone N418; Biolegend), and CD8a (1:200; clone 53-6.7; Biolegend) were used. For all staining of biotinylated antibodies, staining was preceded by a 5-10 min. incubation with FC block (1:200; purified anti-mouse CD16/CD32, clone 2.4G2; Cytek), with 2x antibody cocktail added afterwards. For bulk LMC, following negative enrichment, cells were washed and resuspended in mesenchymal cell

media (DMEM + 1x penicillin/streptomycin, 1x GlutaMAX, 1x sodium pyruvate, 1x nonessential amino acids [all Gibco], and 10% fetal bovine serum [FBS] [Corning]) and passed through 100 μm nylon mesh sheet to eliminate large conglomerates; cells were then washed again and resuspended in mesenchymal cell media for plating. Cells were confirmed to be <2% positive for CD45$^+$, CD31$^+$, or Epcam$^+$ cells following enrichment. For Col14-LMC or Treg cell sorting, post-bead enrichment, cells were stained with fluorescent mouse antibodies for flow cytometric sorting (100 μl per mouse). For Col14-LMC sorting, antibodies used were: CD31-BV605 (1:400; clone 390; Biolegend), Epcam-PerCP-Cy5.5 (1:200; clone G8.8; Biolegend); Pdgfra-PE (1:400; clone APA5; Biolegend), CD146-PE-Cy7 (1:400; clone ME-9F1; Biolegend), CD45-APC (1:400; clone 30-F11; Cytek), and Sca-1-APC-Cy7 (1:400; clone D7; Biolegend). For Treg cell sorting, antibodies used were: CD45-BUV395 (1:400; clone 30-F11; BD), CD11b-BV510 (1:400; clone M1/70; BD), CD11c-BV510 (1:400; clone HL3; BD), TCR β-BV711 (1:200; clone H57-597; BD), CD45R (B220)-PerCP-Cy5.5 (1:200; clone RA3-6B2; Cytek), CD8a-PE (1:200; clone 53-6.7; Cytek), NK1.1-PE-Cy7 (1:200; clone PK136; Biolegend), and CD4-APC (1:100; clone RM4-5; Cytek). Treg cell preparations were done with lungs of *Foxp3*$^{EGFP}$ mice, (i.e., Treg cells express GFP), allowing sorting for GFP$^+$ cells. Cells were incubated with antibodies for 20 min. at 4°C (with shaking at 10 min.). Post-staining, cells were washed, then resuspended in 200 μl per mouse, ran through a 70 μm mesh (for Col14-LMC sorting) or a 40 μm mesh (for Treg cell sorting), then sorted on a BD Aria sorter, with Sytox Blue added 5 min. prior to running samples for dead cell exclusion.

<u>*Flow cytometry*</u>

Adherent cultured cells (LLC cells, A549 cells, bulk LMC, or Col14-LMC) were prepared for flow cytometry by washing with 1x PBS, then rinsing with 0.05% (LLC cells) or 0.25% (A549 cells, LMC) Trypsin-EDTA (Corning) for 5 min., following quenching with media. Ba/F3 cells (non-adherent) were directly removed from wells for flow cytometry. HS-modifying agents used in certain experiments are as described in “Western blotting” (without subsequent ligand treatment). For cell lines/cultured primary cells, antibody staining was performed with 25,000-250,000 cells, with ~10,000 cells ran on a BD LSRII

or BD Fortessa. Lungs were prepared for flow cytometry as described above, with 2-3 million cells from final single cell suspensions used for antibody staining and 100,000-1 million cells ran on a BD LSRII or BD Fortessa. Cells were stained in flow cytometry buffer (1x PBS with 1% BSA [Gold Biotechnology], 2.5 mM ethylenediaminetetraacetic acid [EDTA] disodium salt dihydrate [Fisher], and 0.1% sodium azide [Fisher]). Zombie Violet Fixable Viability Dye (Biolegend) or GhostDye Red 780 (Cytek), stained in a separate step from surface antibodies in 1x PBS, or Sytox Blue (Thermo), added directly to sample 5 min. prior to running on cytometer, were used for dead cell exclusion. For analysis of HS on cell lines or lung single cell suspensions, flow cytometry was done using HS-directed monoclonal mouse IgM antibodies 10E4 and/or JM403 (1:200; AMSBIO), followed by staining with Biotin-SP (long spacer) AffiniPure $F(ab')_2$ Fragment Donkey Anti-Mouse IgM μ chain specific (1:400; Jackson ImmunoResearch), then staining with Streptavidin-APC (1:800; Invitrogen) or Streptavidin-Alexa Fluor 488 (1:800; Invitrogen); for mouse lung single cell suspensions, the Mouse-on-Mouse Immunodetection Kit (Vector Laboratories, Catalog # BMK-2202) was used for these staining steps. For wild-type LLC cells, HS core protein KO LLC cell sublines, and LMC, HS core proteins were assessed with flow cytometry using these anti-mouse antibodies: Sdc1-PE (clone 281-2; Biolegend), Sdc2 (polyclonal sheep IgG; R&D Systems), Sdc3 (polyclonal goat IgG; R&D Systems), Sdc4 (polyclonal rabbit IgG; Sigma-Aldrich), Gpc1 (polyclonal rabbit IgG, Invitrogen), Gpc3 (polyclonal rabbit IgG; Invitrogen), Gpc4 (polyclonal rabbit IgG; Proteintech), Gpc6 (polyclonal goat IgG; R&D Systems), and Hspg2 (rat monoclonal IgG, clone A7L6; Sigma-Aldrich); all staining for HS core proteins was done at 10 μg/ml antibody concentration. Live cells were used for all HS core protein stains, besides for Hspg2 staining where a Cytofix/Cytoperm Kit (BD, Catalog # 554714) was used for fixation/permeabilization of cells. Besides for anti-Sdc1 which was conjugated to PE, cells were then stained with secondary antibodies Donkey Anti-Sheep IgG Alexa Fluor 647, Donkey Anti-Goat IgG Alexa Fluor 647, Donkey Anti-Rabbit IgG Alexa Fluor 647, Donkey Anti-Rat IgG Alexa Fluor 647 (1:400; all Invitrogen), or Donkey Anti-Rabbit Cy5 (1:400; Jackson Immunoresearch). For HS core protein analysis on LMC populations from lung single cell suspensions, cells were blocked with 10% normal horse serum (Gibco) in flow cytometry

buffer, and stained with primary HS core protein antibodies/secondary antibodies in 5% normal horse serum in flow cytometry buffer, prior to staining with other conjugated antibodies to identify LMC subpopulations. For LMC analysis from lung single cell suspensions, surface staining was done using these anti-mouse antibodies: CD45-BUV395 (1:400; clone 30-F11; BD), Pdgfra-BV605 (1:200; clone APA5; Biolegend), CD31-BV711 (1:400; clone 390; Biolegend), Epcam-BV785 (1:200; clone G8.8; Biolegend); CD146-PerCPCy5.5 (1:400; clone ME-9F1; Biolegend), CD49e-PE (1:400; clone 5H10-27[MFR5]; Biolegend), Sca1-PE/Dazzle594 (1:400; clone D7; Biolegend), Pdpn-PE-Cy7 (1:200; clone 8.1.1; Biolegend), and CD9-APC-Fire750 (1:200; clone MZ3; Biolegend). Following surface marker staining, for staining with anti-mouse Ki67-AlexaFluor700 (1:200; clone SolA15; Invitrogen) or anti-mouse α-smooth muscle actin-eFluor660 (1:400; clone 1A4; Invitrogen), cells were fixed/permeabilized with Foxp3/Transcription Factor Staining Buffer Kit (Cytek, Catalog # TNB-0607-KIT). For lymphoid cell analysis from lung single cell suspensions, surface staining was done using these anti-mouse antibodies: NK1.1-BUV395 (1:200; clone PK136; BD), CD11b-BV510 (1:400; clone M1/70; BD), CD11c-BV510 (1:400; clone HL3; BD), CD45-BV786 (1:400; clone 30-F11; BD), CD45R (B220)-PerCP-Cy5.5 (1:200; clone RA3-6B2; Cytek), CD8a-PE (1:200; clone 53-6.7; Cytek), CD90.2 (Thy1.2)-PE-Cy7 (1:200; clone 53-2.1; Biolegend); CD127 (IL-7Ra)-PE-Cy7 (1:200; clone A7R34; Cytek). Following surface staining, for staining lymphoid panel with anti-mouse Foxp3-FITC (1:200; clone FJK-16s; Invitrogen), CD3-BUV496 (1:400; clone 145-2C11; BD), CD4-BUV737 (1:200; clone RM4-5; BD), TCR γ/δ-BV421 (1:200; clone GL3; Biolegend), TCR β-PE/Dazzle594 (1:400; clone H57-597; Biolegend), and biotinylated anti-mouse AREG (1:200; polyclonal goat IgG; R&D systems) followed by Streptavidin-APC (1:800; Thermo) in a separate staining step, cells were fixed/permeabilized with Foxp3/Transcription Factor Staining Buffer Kit (Cytek, Catalog # TNB-0607-KIT). For myeloid cell analysis from lung single cell suspensions, surface staining was done using these anti-mouse antibodies: CD45-BUV395 (1:400; clone 30-F11; BD), CD24-BV510 (1:200; clone M1/69; Biolegend), CD11b-BV650 (1:400; clone M1/70; Biolegend), CD103-BV711 (1:200; clone M290; BD), CD11c-FITC (1:200; clone N418; Cytek), Ly6C-PerCP-Cy5.5 (1:200; clone HK1.4; Biolegend), Siglec-F-PE (1:400;

clone E50-2440; BD), Ly6G-PE/Dazzle594 (1:400; clone 1A8; Biolegend), CD64-PE-Cy7 (1:200; clone X54-5/7.1; Biolegend). For epithelial cell analysis from lung single cell suspensions, surface staining was done using these anti-mouse antibodies: CD45-BUV395 (1:400; clone 30-F11; BD), CD24-BV510 (1:200; clone M1/69; Biolegend), Pdgfra-BV605 (1:200; clone APA5; Biolegend), CD31-BV711 (1:400; clone 390; Biolegend), Epcam-BV785 (1:200; clone G8.8; Biolegend); CD104-APC (1:200; clone 346-11A; Biolegend). For all staining of conjugated antibodies, staining was preceded by a 10 min. incubation with FC block (1:200; purified anti-mouse CD16/CD32, clone 2.4G2; Cytek) in flow cytometry buffer, with 2x antibody cocktail added afterwards. Fluorescence-minus-one (FMO) controls were included to define staining boundaries where necessary.

_Ba/F3 electroporation_

m*Egfr* sequence-containing pCMV6-Entry plasmid (Egfr [NM_207655] Mouse Tagged ORF Clone) was acquired from Origene (MR226160). The m*Egfr* sequence from the plasmid was amplified via PCR with addition of a stop codon at the end and flanking restriction enzyme cut sites (KpnI and NotI, beginning/end respectively), with resultant PCR product purified with Wizard SV Gel and PCR Clean-Up System (Promega, Catalog # A9281). pCDNA3.0 plasmid was acquired from Invitrogen. pCDNA3.0 and amplified m*Egfr* PCR product (with additions) were cut with KpnI and NotI restriction enzymes (New England Biolabs), ran on an agarose gel, cut out of gel, and purified with Wizard SV Gel and PCR Clean-Up System (Promega, Catalog # A9281). T4 DNA Ligase (New England Biolabs, Catalog # M0202S) was used to ligate cut plasmid and cut insert, with the product transformed into DH5α competent cells (Thermo). Individual clones were isolated on carbenicillin (Gold Biotechnology) agar plates, and Sanger sequencing (Azenta) was used to verify correct insertion (primers created by MacVector, ordered from Integrated DNA Technology). pCDNA3.0-m*Egfr* was transiently transfected into Ba/F3 cells using electroporation with a Gene Pulser Xcell Electroporation System (Bio-Rad) (25 µg uncut plasmid, 2.5 million Ba/F3 cells, 4 mm cuvette, 0.975 µF, 0.3 kV), then cultured with G418 (Sigma-Aldrich; 500 µg/ml) and rmIL-3 (Biolegend; 1 ng/ml) in Ba/F3 media (see “Cell Lines”). After

outgrowth of electroporated cells in selection media, cells were assessed for EGFR protein expression and downstream activation in response to rmEGF by western blotting, and single clones were isolated by limiting dilution. A single clone derivative was used for rmEgfr and rmAreg treatment experiments.

*CRISPR-Cas9 knockout line generation in LLC cells*

pSpCas9(BB)-2A-GFP (PX458) was acquired from Addgene (Plasmid # 48138). Single guide RNAs (sgRNAs) for selected mouse genes were generated using the Broad Institute sgRNA Designer. For each gene, two sgRNAs were selected targeting different exons (Supplementary Table 2). Selected sgRNAs were produced by Integrated DNA Technologies, with added overhangs for annealing into BbsI restriction enzyme (New England Biolabs)-cut PX458 (CACCG[sgRNA sequence] on forward annealing half and AAAC[sgRNA reverse complement sequence]C on reverse annealing half). sgRNAs were cloned into PX458 in a one-step reaction with BbsI and Quick Ligase (New England Biolabs, Catalog # M2200S), with products transformed into DH5α competent cells (Thermo). Individual clones were isolated on carbenicillin (Gold Biotechnology) agar plates, and Sanger sequencing (Azenta) was used to verify correct insertion (primers ordered from Integrated DNA Technology). Wild-type LLC cells were transfected with a mixture of Lipofectamine-2000 (Thermo) and both sgRNA-PX458 constructs (i.e., for both exons simultaneously) for each gene, diluted in Opti-MEM (Thermo). Media was changed after 6h, and 36h after transfection, cells were sorted on a BD Aria for $GFP^+$ cells, into individual wells. Single cell clonal lines of cells were grown, and screened at the DNA level using specific primers for each exon targeted in each gene. Clones with DNA alteration were further analyzed at the RNA level (see “RNA extraction and qPCR”). Protein-level confirmation of KO or HS-level alterations conferred by KO was done with flow cytometry, as described above. For certain experiments, empty PX458 was transfected into LLC cells to create control cell lines. Sublines were subsequently used for western blotting experiments.

*Transduction of LLC cells for knockout rescue*

MGC Mouse *Gpc1* cDNA (Horizon Discovery MMM1013-202859423) was acquired in a pYX-Asc vector. MSCV2.2 retroviral vector was acquired from Addgene (Plasmid #60206). Gibson assembly was used to clone *Gpc1* cDNA coding region into MSCV2.2 (strategy designed in MacVector). *Gpc1* was amplified from the original delivery vector, with primers including Gibson assembly overhangs, with Q5 High Fidelity 2X Master Mix (New England Biolabs, Catalog # M0492S), with resultant PCR product purified with Wizard SV Gel and PCR Clean-Up System (Promega, Catalog # A9281). MSCV2.2 was cut with NotI (New England Biolabs), then ran on an agarose gel, cut out of gel, and purified with Wizard SV Gel and PCR Clean-Up System (Promega, Catalog # A9281). Gibson Assembly Master Mix (New England Biolabs, Catalog # 2611S) was used with amplified *Gpc1* cDNA and cut MSCV2.2 to create MSCV2.2 with *Gpc1* insert (MSCV-*Gpc1*). Individual clones were isolated on carbenicillin (Gold Biotechnology) agar plates, and Sanger sequencing (Azenta) was used to verify correct insertion (primers created by MacVector, ordered from Integrated DNA Technology). MSCV2.2 (MSCV-Empty vector [EV]) or MSCV-*Gpc1* were transfected into Phoenix-ECO cells with a mixture of plasmid and Lipofectamine-2000 (Thermo) diluted in Opti-MEM (Thermo). Transfected cells were passaged at 24h. At 48h post-transfection, viral supernatants were removed from wells, centrifuged at 450 x g/room temperature/5 min., and supernatants were run through a 0.45 μm filter (Corning). Hexadimethrine bromide (polybrene) (5 ug/ml) Sigma-Aldrich) was added to filtered supernatants, then Gpc1-KO LLC cells were treated with viral supernatants. After ~12h incubation, media was replaced, and transduced cells were passaged at 36h post-transfection. 48h later, transduced cells were sorted on a BD Aria for $GFP^+$ cells (bulk sorted). Transduced cell lines were confirmed to maintain GFP and Gpc1 protein expression over several passages, and subsequently used for flow cytometry and western blotting experiments.

*siRNA treatment*

ON-TARGETplus siRNA was acquired from Horizon Discovery/Dharmacon, with SMARTPools (4 siRNA constructs included) for mouse *Sdc2*, mouse *Gpc4*, or non-targeting siRNA control. Bulk LMC

were isolated as described above and plated in mesenchymal cell media at 2 million cells/well in 6 well tissue culture-treated plates (Corning). Cells were allowed to adhere overnight (16-18h); the following day, media was aspirated from cells, cells were washed with DMEM (Gibco) to remove dead cells, and fresh mesenchymal cell media was added. Bulk LMC were then transfected with a mixture of siRNA and Lipofectamine RNAiMax Transfection Reagent (Thermo) diluted in Opti-MEM (Thermo). 48h post-treatment (no media change, siRNA left in wells throughout), cells were used for flow cytometry or western blotting experiments.

*Col14-LMC/Treg cell co-culture*

Col14-LMC were isolated/sorted as described above and plated in mesenchymal cell media at 40,000-50,000 cells/well in 48 well tissue culture-treated plates (Corning). Cells were allowed to adhere overnight (16-18h); the following day, media was aspirated from cells, cells were washed with RPMI (Gibco) to remove dead cells, and fresh T cell media (RPMI + 100x penicillin/streptomycin, 100x GlutaMAX, 100x HEPES, 100x sodium pyruvate, 100x nonessential amino acids, 1000x β-mercaptoethanol [all Gibco], and 10% fetal bovine serum [FBS]) was added; cells were rested for ~12h while Treg cell isolation/sorting occurred. Treg cells from lungs of IAV-infected mice (8 d.p.i.) were isolated/sorted as described above. 20,000-25,000 Treg cells (1:2 Treg cell:Col14-LMC ratio) were then added directly to Col14-LMC wells (no media change) in small volumes (1:25 volume of media in wells). Dynabeads Mouse T-Activator CD3/CD28 for T-Cell Expansion and Activation Beads (Thermo) were added to Treg cells at a 1:1 Treg cell/bead ratio prior to addition to wells. rhIL-2 (200 U/ml; NCI Preclinical Repository) and rhIL-7 (10 ng/ml; NCI Preclinical Repository) were added directly to wells at time of Treg cell addition. Cell co-cultures were incubated for 12h. At this time, wells were subjected to one wash of 5 mM EDTA in 1x PBS, and two additional washes of 1x PBS to remove Treg cells; following this removal, Col14-LMC in wells were lysed and analyzed for RNA (see “RNA extraction and qPCR”).

**Supplementary Table 1. qPCR primers used in this study.**

| | |
|---|---|
| *Hprt*-FW | TCAGTCAACGGGGGACATAAA |
| *Hprt*-RV | GGGGCTGTACTGCTTAACCAG |
| *Ext1*-FW | AGTTCTTGTGGGAGGCTTATTT |
| *Ext1*-RV | CCTCCAGGATGTTTGTTCCATA |
| *Ext2*-FW | ACAGAGGCAGATTGAAGAGATG |
| *Ext2*-RV | GGAGATGGCTGCATATGGATAG |
| *Ndst1*-FW | AGAGGAGTACCCACATCTGAA |
| *Ndst1*-RV | TGTGTGTAAAGAGGCCACAG |
| *Hs2st1*-FW | CCAGGATTAAGGAGACGGAAAC |
| *Hs2st1*-RV | CACAGAAGAACGGGATCTGAAG |
| *Hs6st1*-FW | ACCGAACTCACCAACTGTG |
| *Hs6st1*-RV | CAGCAGGGTGATGTAGTAGAAC |
| *Sdc1*-FW | AAAGAGGTTGTCGAGGATGG |
| *Sdc1*-RV | CCCAGATGTTTCAAAGGTGAAG |
| *Sdc2*-FW | CACAGATGTGTACACGGAGAA A |
| *Sdc2*-RV | GGTACACCAATAGCAGGATGAG |
| *Gpc1*-FW | TTGCCGAAATGTGCTCAAAG |
| *Gpc1*-RV | CCCAGAACTTGTCAGTGATGA |
| *Hspg2*-FW | GCTGCCAGAAATATACCAAGGA |
| *Hspg2*-RV | TCTGGATATCAGGGTCCAAGAG |
| *PR8 NS1*-FW | TGTCAAGCTTTCAGGTAGATTG |
| *PR8 NS1*-RV | CTCTTAGGGATTTCTGATCTC |
| *PR8 M protein*-FW | GGACTGCAGCGTTAGACGCTT |
| *PR8 M protein*-RV | CATCCTGTTGTATATGAGGCCCAT |
| *Lif*-FW | AAACGGCCTGCATCTAAGG |
| *Lif*-RV | GCAGAACCAGCAGCAGTAA |
| *Il6*-FW | CTCTCTGCAAGAGACTTCCATC |
| *Il6*-RV | CTCCGACTTGTGAAGTGGTATAG |
| *Il11*-FW | TGCTCACACTCACAAACCTCC |
| *Il11*-RV | CAGGCGACAAACACAGTTCAT |
| *Vegfa*-FW | ACGACAGAAGGAGAGCAGAA |
| *Vegfa*-RV | ATGTCCACCAGGGTCTCAA |

**Supplementary Table 2. CRISPR-Cas9 sgRNAs used in this study.**

| | |
|---|---|
| *Ext1*_Exon1_FW | CACCGCTTATATCACGTCCATAACG |
| *Ext1*_Exon1_RV | AAACCGTTATGGACGTGATATAAGC |
| *Ext1*_Exon3_FW | CACCGTTGGTTCCAATTAATCACTT |
| *Ext1*_Exon3_RV | AAACAAGTGATTAATTGGAACCAAC |
| *Ext2*_Exon3_FW | CACCGAGGGCAGTGTTGTAATCTGG |
| *Ext2*_Exon3_RV | AAACCCAGATTACAACACTGCCCTC |
| *Ext2*_Exon5_FW | CACCGCAAATGCACCAATCTCTCGG |
| *Ext2*_Exon5_RV | AAACCCGAGAGATTGGTGCATTTGC |
| *Ndst1*_Exon3_FW | CACCGGGCCGTGTCACATAGAGCAG |
| *Ndst1*_Exon3_RV | AAACCTGCTCTATGTGACACGGCCC |
| *Ndst1*_Exon6_FW | CACCGCACAGATATGGGCTATGCGG |
| *Ndst1*_Exon6_RV | AAACCCGCATAGCCCATATCTGTGC |
| *Hs2st1*_Exon2_FW | CACCGGTAGGCTATATTGGTGAACG |
| *Hs2st1*_Exon2_RV | AAACCGTTCACCAATATAGCCTACC |
| *Hs2st1*_Exon4_FW | CACCGAATTTATATCAATGTCATCA |
| *Hs2st1*_Exon4_RV | AAACTGATGACATTGATATAAATTC |
| *Hs6st1*_Exon1_FW | CACCGGGCGCACGTTCTGCACTAGG |
| *Hs6st1*_Exon1_RV | AAACCCTAGTGCAGAACGTGCGCCC |
| *Hs6st1*_Exon2_FW | CACCGCACTCAGGTAGCGGGATACG |
| *Hs6st1*_Exon2_RV | AAACCGTATCCCGCTACCTGAGTGC |
| *Sdc1*_Exon3_FW | CACCGGTCACATCTCATCCGCACGG |
| *Sdc1*_Exon3_RV | AAACCCGTGCGGATGAGATGTGACC |
| *Sdc1*_Exon4_FW | CACCGCCTGCGGAATCAGCCCCCGG |
| *Sdc1*_Exon4_RV | AAACCCGGGGGCTGATTCCGCAGGC |
| *Sdc2*_Exon3_FW | CACCGTAGTGCTGCTTCCCCCAAAG |
| *Sdc2*_Exon3_RV | AAACCTTTGGGGGAAGCAGCACTAC |
| *Sdc2*_Exon4_FW | CACCGGGAGGAAGTTGACATTTCTG |
| *Sdc2*_Exon4_RV | AAACCAGAAATGTCAACTTCCTCCC |
| *Gpc1*_Exon1_FW | CACCGCGAAGTCCGCCAGATCTACG |
| *Gpc1*_Exon1_RV | AAACCGTAGATCTGGCGGACTTCGC |
| *Gpc1*_Exon3_FW | CACCGGCTGCGCCTCTACTACCGTG |
| *Gpc1*_Exon3_RV | AAACCACGGTAGTAGAGGCGCAGCC |
| *Hspg2*_Exon12_FW | CACCGAGGGTGACAATGACCAGCGA |
| *Hspg2*_Exon12_RV | AAACTCGCTGGTCATTGTCACCCTC |
| *Hspg2*_Exon28_FW | CACCGTACTATGGGGATGCCCAACG |
| *Hspg2*_Exon28_RV | AAACCGTTGGGCATCCCCATAGTAC |
