# Supplementary material for: Heparan sulfate regulates amphiregulin programming of tissue reparative lung mesenchymal cells during influenza A virus infection in mice
Source: Nat Commun. 2025 Mar 3;16:2129. doi: 10.1038/s41467-025-57362-z (PMC11876457; doi:10.1038/s41467-025-57362-z)
Supplement: Supplementary file 2 — Reporting Summary [file 41467_2025_57362_MOESM2_ESM.pdf]

nature portfolio

Corresponding author(s): Nicholas Arpaia

Last updated by author(s): Feb 5, 2025

Reporting Summary

Nature Portfolio wishes to improve the reproducibility of the work that we publish. This form provides structure for consistency and transparency in reporting. For further information on Nature Portfolio policies, see our Editorial Policies and the Editorial Policy Checklist.

Statistics

For all statistical analyses, confirm that the following items are present in the figure legend, table legend, main text, or Methods section.

n/a	Confirmed	
☐	☒	The exact sample size (*n*) for each experimental group/condition, given as a discrete number and unit of measurement
☐	☒	A statement on whether measurements were taken from distinct samples or whether the same sample was measured repeatedly
☐	☒	The statistical test(s) used AND whether they are one- or two-sided<br>*Only common tests should be described solely by name; describe more complex techniques in the Methods section.*
☐	☒	A description of all covariates tested
☐	☒	A description of any assumptions or corrections, such as tests of normality and adjustment for multiple comparisons
☐	☒	A full description of the statistical parameters including central tendency (e.g. means) or other basic estimates (e.g. regression coefficient) AND variation (e.g. standard deviation) or associated estimates of uncertainty (e.g. confidence intervals)
☐	☒	For null hypothesis testing, the test statistic (e.g. *F*, *t*, *r*) with confidence intervals, effect sizes, degrees of freedom and *P* value noted<br>*Give P values as exact values whenever suitable.*
☒	☐	For Bayesian analysis, information on the choice of priors and Markov chain Monte Carlo settings
☒	☐	For hierarchical and complex designs, identification of the appropriate level for tests and full reporting of outcomes
☒	☐	Estimates of effect sizes (e.g. Cohen's *d*, Pearson's *r*), indicating how they were calculated

Our web collection on statistics for biologists contains articles on many of the points above.

Software and code

Policy information about availability of computer code

Data collection	No original code was used in this study for data collection.
Data analysis	No original code was used in this study for data analysis. Software used for data analysis in this study includes bclfastq2 (version 2.19), kallisto (version 0.44.0), iDEP (version 1.13), g:Profiler (version: database built on 2022-12-28), Seurat (version 3.0), QuPath (version 0.3.0), FlowJo (version 10.7.1), SnapGene Viewer (version 6.0.4), Adobe Illustrator (version 24.1.2), ImageJ (version 1.53m), MacVector (version 17.0), R (version 4.2.2), Microsoft Excel (version 16.0), and Microsoft Powerpoint (version 16.0)

For manuscripts utilizing custom algorithms or software that are central to the research but not yet described in published literature, software must be made available to editors and reviewers. We strongly encourage code deposition in a community repository (e.g. GitHub). See the Nature Portfolio guidelines for submitting code & software for further information.

Data

Policy information about availability of data

All manuscripts must include a data availability statement. This statement should provide the following information, where applicable:

- Accession codes, unique identifiers, or web links for publicly available datasets
- A description of any restrictions on data availability
- For clinical datasets or third party data, please ensure that the statement adheres to our policy

RNA-seq data associated with this manuscript have been deposited in NCBI's Gene Expression Omnibus under GEO accession number GSE263616. All other data have been included in the manuscript or Source Data.

Research involving human participants, their data, or biological material

Policy information about studies with human participants or human data. See also policy information about sex, gender (identity/presentation), and sexual orientation and race, ethnicity and racism.

Reporting on sex and gender	No human participants were used in this study.
Reporting on race, ethnicity, or other socially relevant groupings	No human participants were used in this study.
Population characteristics	No human participants were used in this study.
Recruitment	No human participants were used in this study.
Ethics oversight	No human participants were used in this study.

Note that full information on the approval of the study protocol must also be provided in the manuscript.

Field-specific reporting

Please select the one below that is the best fit for your research. If you are not sure, read the appropriate sections before making your selection.

- [x] Life sciences
- [ ] Behavioural & social sciences
- [ ] Ecological, evolutionary & environmental sciences

For a reference copy of the document with all sections, see nature.com/documents/nr-reporting-summary-flat.pdf

Life sciences study design

All studies must disclose on these points even when the disclosure is negative.

Sample size	As determined by previous studies, 3 separate experiments were performed in experiments involving western blotting. As determined by previous studies, in vivo experimentation was done using 3 separate cohorts of mice (~5 mice per group).
Data exclusions	As detailed in Methods, for influenza A infection experiments, mice were disincluded from final analysis if they experienced loss of SpO2 to a level <85% at 3-4 d.p.i. (as this is an indication of lung damage from technical issues with administration, not from IAV infection), if they did not experience a loss of body temperature <38°C at 7 d.p.i. (as this is evidence of administration issues leading to an unproductive infection), or if there was visible failure to uptake IAV intranasally during infection.
Replication	All attempts at replication were successful.
Randomization	Samples for in vivo experiments were sorted in experimental groups based on genotype, with order of experimentation randomly determined by genotyping order. Each cage included mice from both genotypes, to control for any microbiota differences between cages.
Blinding	Histology analysis on IAV-infected lung sections was done in a blinded manner. No other measurements taken in this study utilized metrics that could be subject to bias by blinding status, thus no blinding was necessary in assessment of experimental/disease parameters.

Reporting for specific materials, systems and methods

We require information from authors about some types of materials, experimental systems and methods used in many studies. Here, indicate whether each material, system or method listed is relevant to your study. If you are not sure if a list item applies to your research, read the appropriate section before selecting a response.

Materials & experimental systems

n/a	Involved in the study	
	x	Antibodies
	x	Eukaryotic cell lines
x		Palaeontology and archaeology
	x	Animals and other organisms
x		Clinical data
x		Dual use research of concern
x		Plants

Methods

n/a	Involved in the study	
x		ChIP-seq
	x	Flow cytometry
x		MRI-based neuroimaging

Antibodies

Antibodies used

For western blotting, antibodies used were β-Actin (1:1000; clone 8H10D10; Cell Signaling), Phospho-EGF Receptor Tyr1068 (1:1000; clone D7A5; Cell Signaling), Phospho-Akt Receptor Ser473 (1:2000; clone D9E; Cell Signaling), Phospho-p44/42 MAPK [Erk1/2] Thr202/Tyr204 (1:2000; clone D13.14.4E; Cell Signaling), EGF Receptor (1:1000; clone C74B9; Cell Signaling), anti-mouse IgG HRP-linked antibody (1:5000; Cell Signaling), anti-rabbit IgG HRP-linked antibody (1:2500; Cell Signaling). For streptavidin bead-based enrichment, antibodies used were (all anti-mouse) (all 1:200 dilution): CD45-biotin (clone 30-F11; Biolegend), CD31-biotin (clone 390; Biolegend), Epcam-biotin (clone G8.8; Biolegend), TER-119-biotin (clone TER-119; Biolegend), Pdgfra-biotin (clone APA5; Biolegend), CD19-biotin (clone 6D5; Biolegend), NK1.1-biotin (clone PK136; Biolegend), CD11b-biotin (clone M1/70; Biolegend), CD11c-biotin (clone N418; Biolegend), CD8a-biotin (clone 53-6.7; Biolegend). For Col14-lung mesenchymal cell sorting, antibodies used were (all anti-mouse): CD31-BV605 (1:400; clone 390; Biolegend), Epcam-PerCP-Cy5.5 (1:200; clone G8.8; Biolegend); Pdgfra-PE (1:400; clone APA5; Biolegend), CD146-PE-Cy7 (1:400; clone ME-9F1; Biolegend), CD45-APC (1:400; clone 30-F11; Cytek), Sca-1-APC-Cy7 (1:400; clone D7; Biolegend). For Treg cell sorting, antibodies used were (all anti-mouse): CD45-BUV395 (1:400; clone 30-F11; BD), CD11b-BV510 (1:400; clone M1/70; BD), CD11c-BV510 (1:400; clone HL3; BD), TCR β-BV711 (1:200; clone H57-597; BD), CD45R (B220)-PerCP-Cy5.5 (1:200; clone RA3-6B2; Cytek), CD8a-PE (1:200; clone 53-6.7; Cytek), NK1.1-PE-Cy7 (1:200; clone PK136; Biolegend), CD4-APC (1:100; clone RM4-5; Cytek). For heparan sulfate core proteins, antibodies used were (all anti-mouse) (all diluted to 10 μg/ml): Sdc1-PE (clone 281-2; Biolegend), Sdc2 (polyclonal sheep IgG; R&D Systems), Sdc3 (polyclonal goat IgG; R&D Systems), Sdc4 (polyclonal rabbit IgG; Sigma-Aldrich), Gpc1 (polyclonal rabbit IgG, Invitrogen), Gpc3 (polyclonal rabbit IgG; Invitrogen), Gpc4 (polyclonal rabbit IgG; Proteintech), Gpc6 (polyclonal goat IgG; R&D Systems), Hspg2 (rat monoclonal IgG, clone A7L6; Sigma-Aldrich). For analysis of heparan sulfate, antibodies used were HS-directed monoclonal mouse IgM 10E4 antibody or JM403 antibody (1:200; AMSBIO), followed by staining with Biotin-SP (long spacer) AffiniPure F(ab')$_2$ Fragment Donkey Anti-Mouse IgM μ chain specific (1:400; Jackson ImmunoResearch), then staining with Streptavidin-APC (1:800; Invitrogen) or Streptavidin-Alexa Fluor 488 (1:800; Invitrogen). For lung mesenchymal cell staining, antibodies used were (all anti-mouse): CD45-BUV395 (1:400; clone 30-F11; BD), Pdgfra-BV605 (1:200; clone APA5; Biolegend), CD31-BV711 (1:400; clone 390; Biolegend), Epcam-BV785 (1:200; clone G8.8; Biolegend); CD146-PerCPCy6.6 (1:400; clone ME-9F1; Biolegend), CD49e-PE (1:400; clone 5H10-27[MFR5]; Biolegend), Sca1-PE/Dazzle594 (1:400; clone D7; Biolegend), Pdpn-PE-Cy7 (1:200; clone 8.1.1; Biolegend), CD9-APC-Fire750 (1:200; clone MZ3; Biolegend), Ki67-AlexaFluor700 (1:200; clone SolA15; Invitrogen), α-smooth muscle actin-eFluor660 (1:400; clone 1A4; Invitrogen). For lung lymphoid cell staining, antibodies used were (all anti-mouse): NK1.1-BUV395 (1:200; clone PK136; BD), CD3-BUV496 (1:400; clone 145-2C11; BD), CD4-BUV737 (1:200; clone RM4-5; BD), TCR γ/δ-BV421 (1:200; clone GL3; Biolegend), CD11b-BV510 (1:400; clone M1/70; BD), CD11c-BV510 (1:400; clone HL3; BD), CD45-BV786 (1:400; clone 30-F11; BD), CD45R (B220)-PerCP-Cy5.5 (1:200; clone RA3-6B2; Cytek), CD8a-PE (1:200; clone 53-6.7; Cytek), TCR β-PE/Dazzle594 (1:400; clone H57-597; Biolegend), CD90.2 (Thy1.2)-PE-Cy7 (1:200; clone 53-2.1; Biolegend); CD127 (IL-7Ra)-PE-Cy7 (1:200; clone A7R34; Cytek), Foxp3-FITC (1:200; clone FJK-16s; Invitrogen), biotinylated anti-mouse AREG (1:200; polyclonal goat IgG; R&D systems) followed by Streptavidin-APC (1:800; Thermo). For lung myeloid cell staining, antibodies used were (all anti-mouse): CD45-BUV395 (1:400; clone 30-F11; BD), CD24-BV510 (1:200; clone M1/69; Biolegend), CD11b-BV650 (1:400; clone M1/70; Biolegend), CD103-BV711 (1:200; clone M290; BD), CD11c-FITC (1:200; clone N418; Cytek), Ly6C-PerCP-Cy5.5 (1:200; clone HK1.4; Biolegend), Siglec-F-PE (1:400; clone E50-2440; BD), Ly6G-PE/Dazzle594 (1:400; clone 1A8; Biolegend), CD64-PE-Cy7 (1:200; clone X54-5/7.1; Biolegend). For lung epithelial cell staining, antibodies used were (all anti-mouse): CD45-BUV395 (1:400; clone 30-F11; BD), CD24-BV510 (1:200; clone M1/69; Biolegend), Pdgfra-BV605 (1:200; clone APA5; Biolegend), CD31-BV711 (1:400; clone 390; Biolegend), Epcam-BV785 (1:200; clone G8.8; Biolegend); CD104-APC (1:200; clone 346-11A; Biolegend). For all staining of conjugated antibodies, FC block (1:200; purified anti-mouse CD16/CD32, clone 2.4G2; Cytek) was used.

Validation

Validation of each antibody was done by the companies (included in the product descriptions for each antibody), and were confirmed by our laboratory in western blot and flow cytometry experiments included in the manuscript (see gating strategies and knockout cell line validation).

Eukaryotic cell lines

Policy information about cell lines and Sex and Gender in Research

Cell line source(s)

LLC (also known as LL/2 or LLC1) (male): ATCC (Catalog # CRL-1642); Phoenix-ECO (female): ATCC (Catalog # CRL-3214); A549 (male): Richard Vallee Lab at Columbia University; Ba/F3 (gender unknown): Michael Green Lab at UMass Chan Medical School

Authentication

None of the cell lines used were authenticated.

Mycoplasma contamination

All cell lines tested negative for mycoplasma contamination.

Commonly misidentified lines (See ICLAC register)	None

Animals and other research organisms

Policy information about studies involving animals; ARRIVE guidelines recommended for reporting animal research, and Sex and Gender in Research

Laboratory animals	Mus musculus, C57BL/6, 8-12 weeks old
Wild animals	This study did not involve wild animals.
Reporting on sex	For influenza A virus in vivo experiments, both male and female mice were used in experiments. For bleomycin in vivo experiments, only male mice were used in experiments, based on past research showing suboptimal model induction in females.
Field-collected samples	This study did not involve samples collected from the field.
Ethics oversight	Columbia University Institutional Animal Care and Use Committee (protocol AC-AABT2656)

Note that full information on the approval of the study protocol must also be provided in the manuscript.

Plants

Seed stocks	No plants were used in this study.
Novel plant genotypes	No plants were used in this study.
Authentication	No plants were used in this study.

Flow Cytometry

Plots

Confirm that:

- [ ] The axis labels state the marker and fluorochrome used (e.g. CD4-FITC).
- [x] The axis scales are clearly visible. Include numbers along axes only for bottom left plot of group (a 'group' is an analysis of identical markers).
- [x] All plots are contour plots with outliers or pseudocolor plots.
- [x] A numerical value for number of cells or percentage (with statistics) is provided.

Methodology

Sample preparation	See Methods for detailed description.
Instrument	BD LSR Fortessa, BD LSR II, BD FACSAria
Software	BD FACSDiva, FlowJo
Cell population abundance	Abundances are described by percentages in gating strategies. Post-sort purity was determined for sorted cell populations by re-running sorted cells through the flow cytometer and determining the majority of cells lied in the proper gates.
Gating strategy	See Supplementary Figures/Methods

- [x] Tick this box to confirm that a figure exemplifying the gating strategy is provided in the Supplementary Information.
