# Supplementary figures and images for: Heparan sulfate regulates amphiregulin programming of tissue reparative lung mesenchymal cells during influenza A virus infection in mice
Source: Nat Commun. 2025 Mar 3;16:2129. doi: 10.1038/s41467-025-57362-z (PMC11876457; doi:10.1038/s41467-025-57362-z)

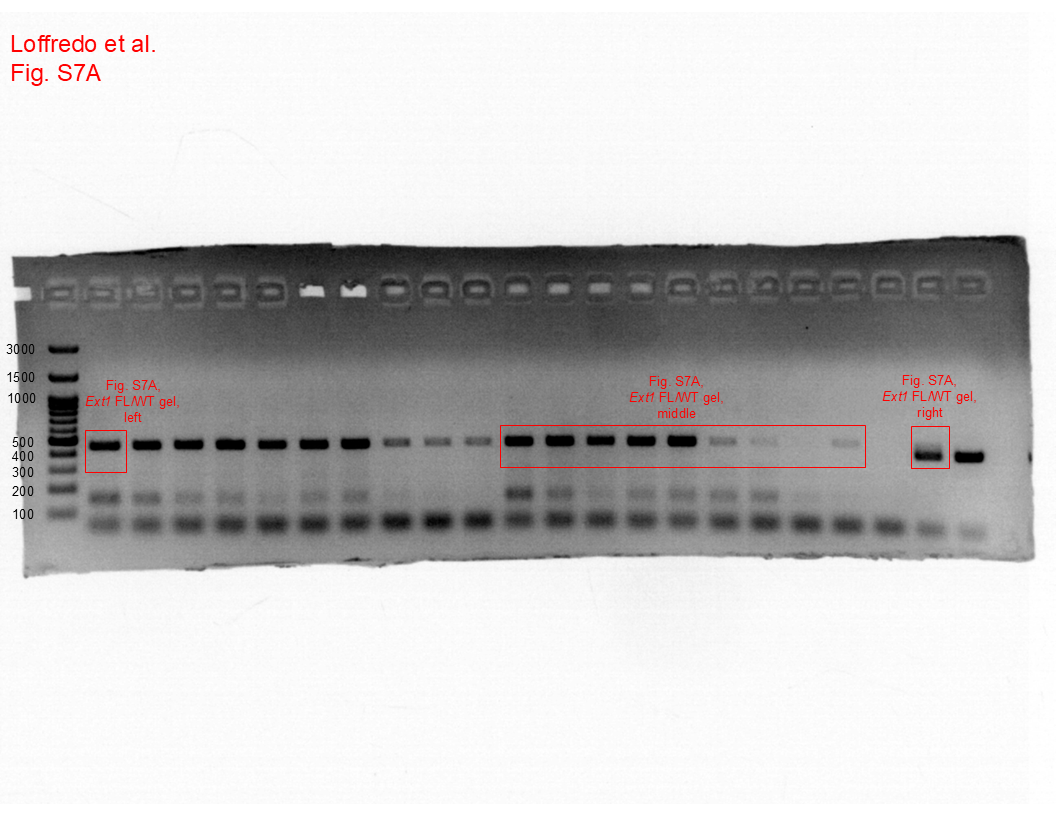

Supplement: Supplementary file 4 — Source Data [file 41467_2025_57362_MOESM4_ESM.zip › SourceData/DNAgel_FigS7A_Ext1-FL_Ext1-WT.tif]

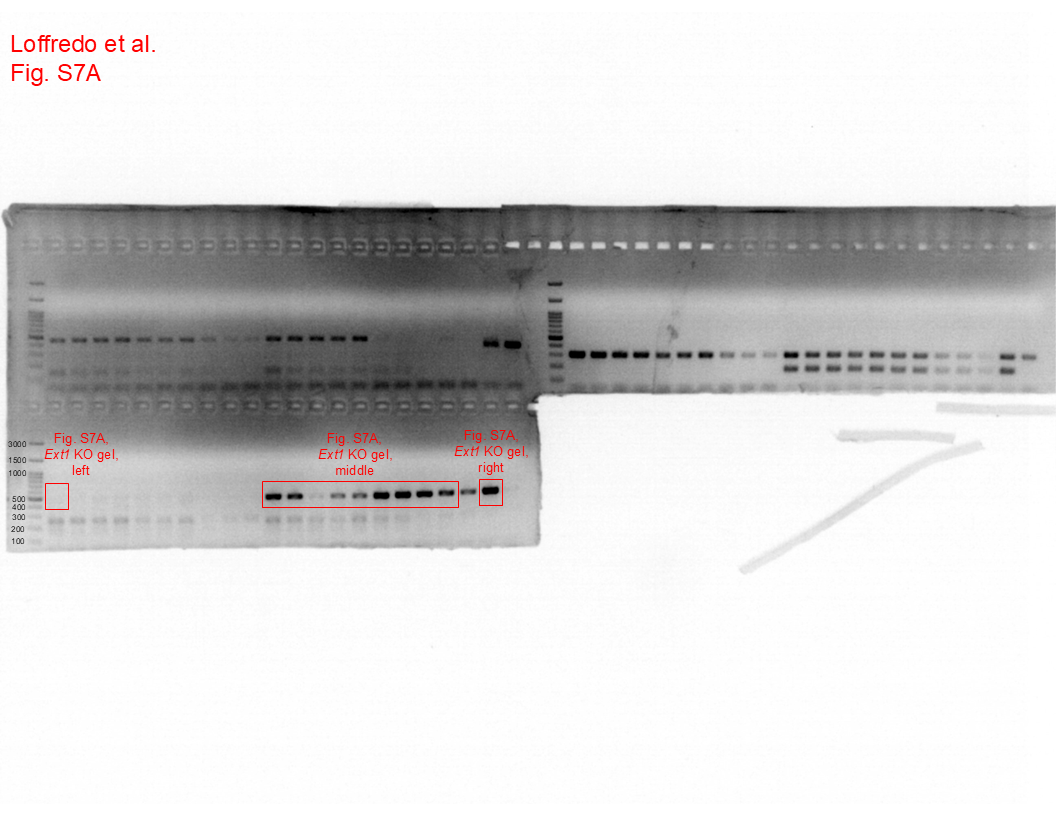

Supplement: Supplementary file 4 — Source Data [file 41467_2025_57362_MOESM4_ESM.zip › SourceData/DNAgel_FigS7A_Ext1-KO.tif]

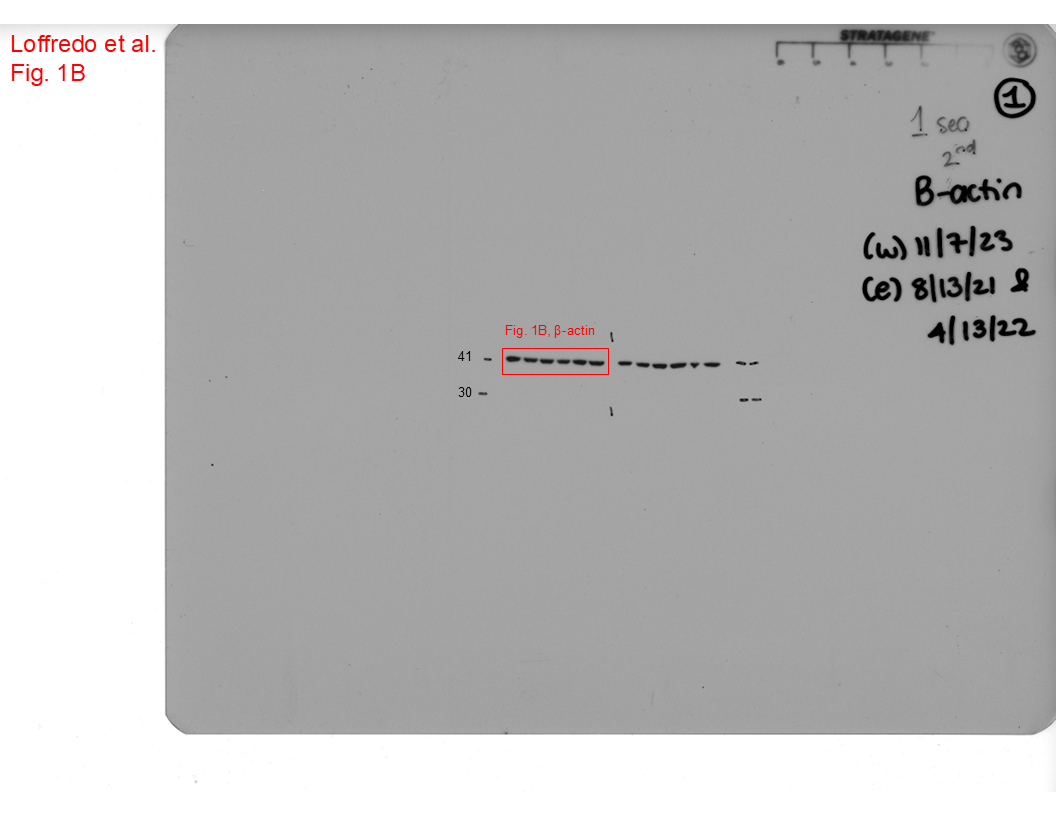

Supplement: Supplementary file 4 — Source Data [file 41467_2025_57362_MOESM4_ESM.zip › SourceData/Western_Fig1B_bActin.tif]

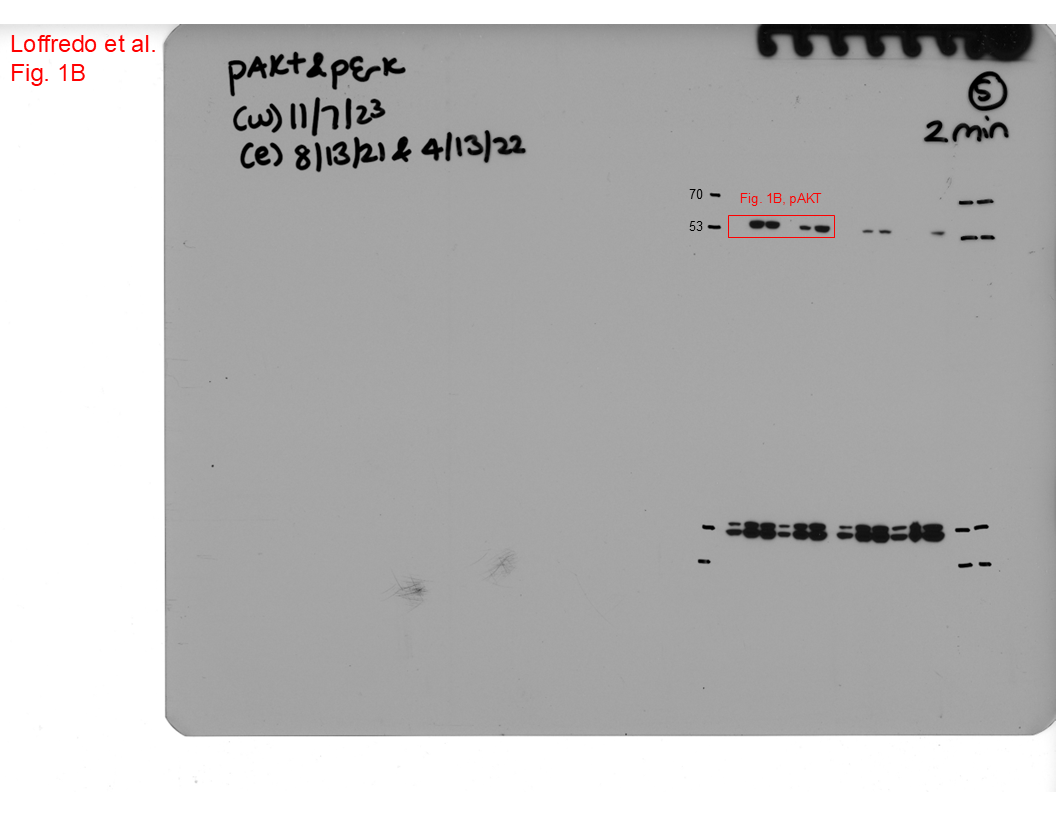

Supplement: Supplementary file 4 — Source Data [file 41467_2025_57362_MOESM4_ESM.zip › SourceData/Western_Fig1B_pAKT.tif]

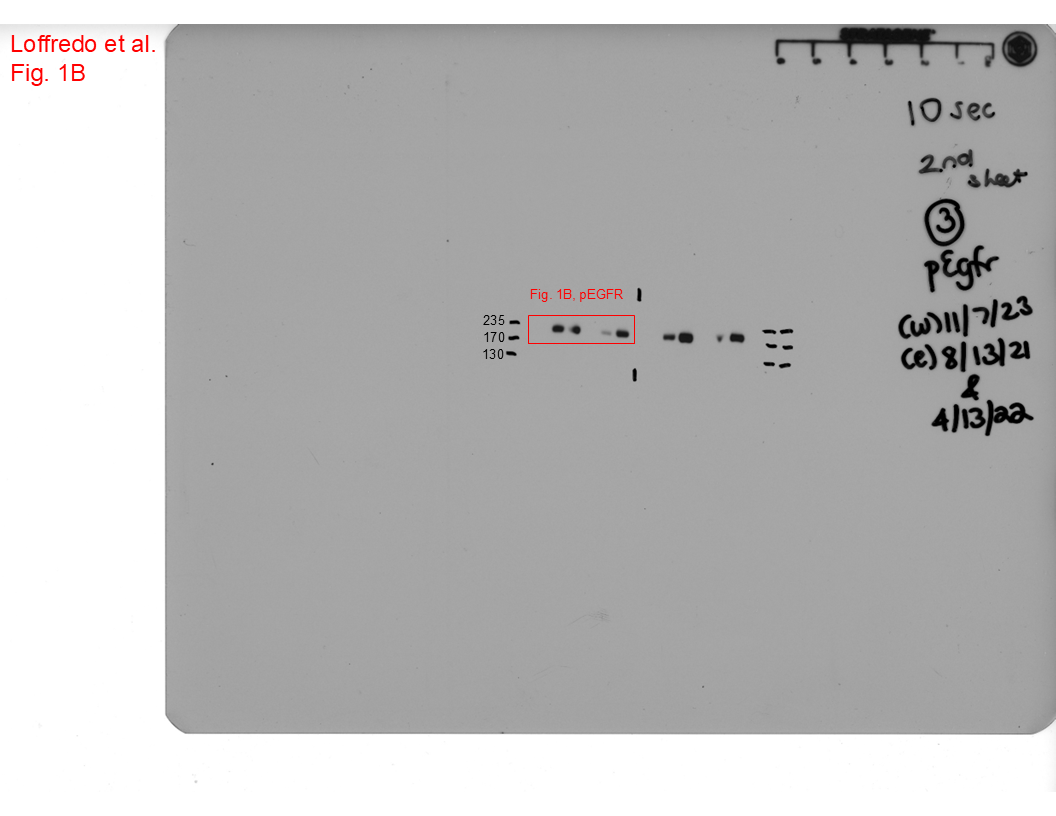

Supplement: Supplementary file 4 — Source Data [file 41467_2025_57362_MOESM4_ESM.zip › SourceData/Western_Fig1B_pEGFR.tif]

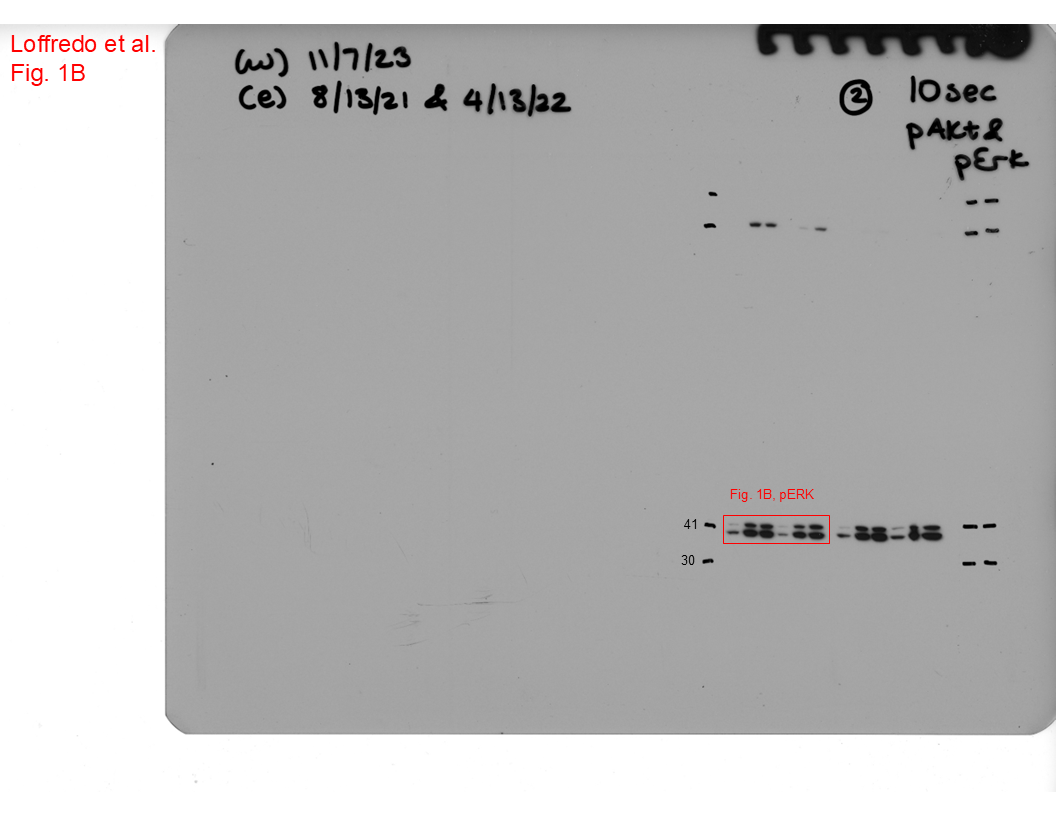

Supplement: Supplementary file 4 — Source Data [file 41467_2025_57362_MOESM4_ESM.zip › SourceData/Western_Fig1B_pERK.tif]

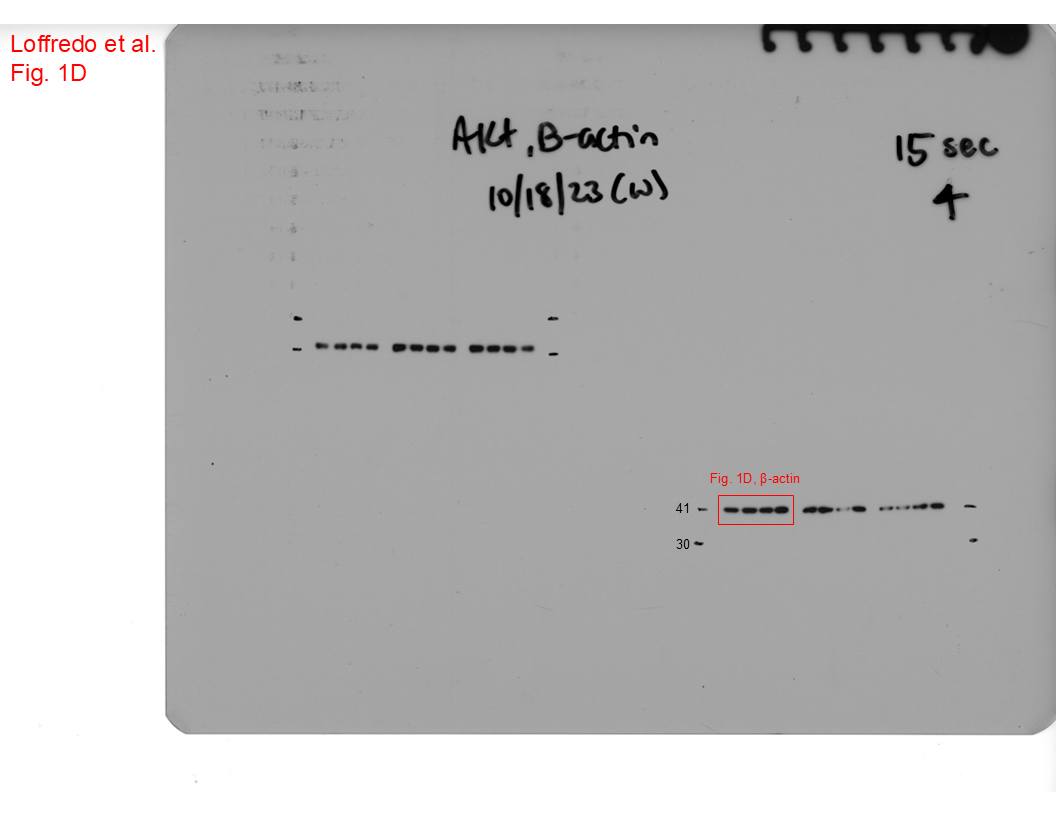

Supplement: Supplementary file 4 — Source Data [file 41467_2025_57362_MOESM4_ESM.zip › SourceData/Western_Fig1D_bActin.tif]

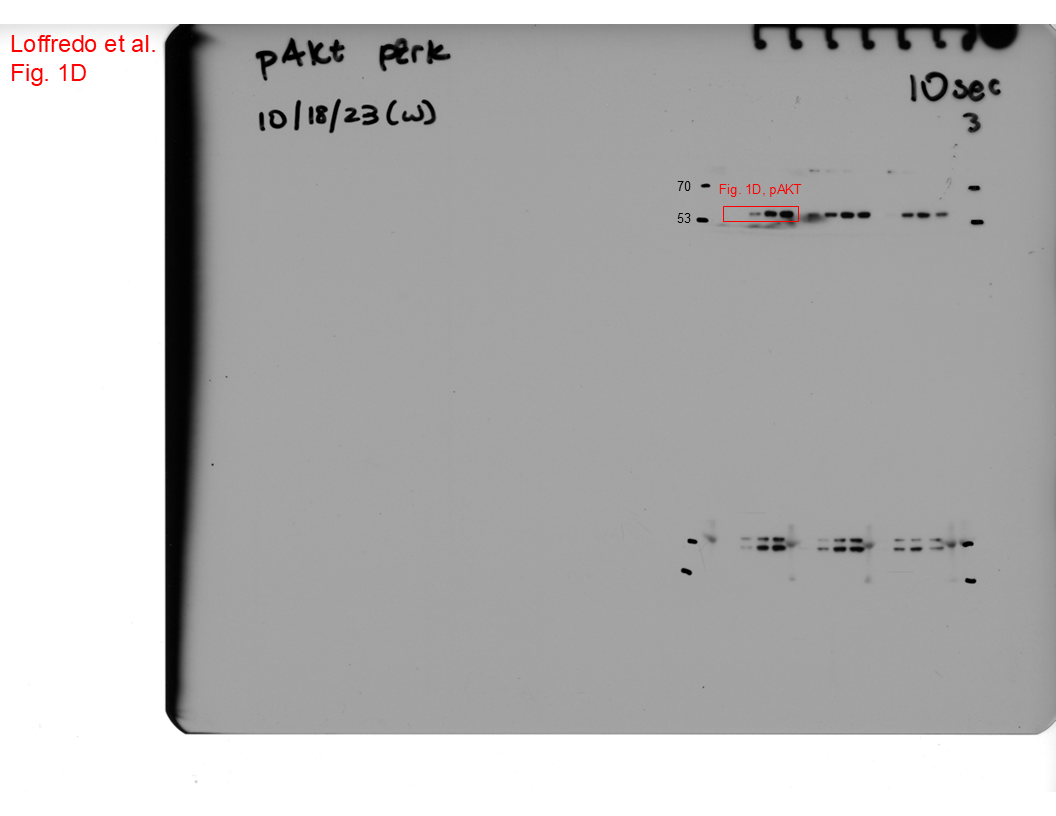

Supplement: Supplementary file 4 — Source Data [file 41467_2025_57362_MOESM4_ESM.zip › SourceData/Western_Fig1D_pAKT.tif]

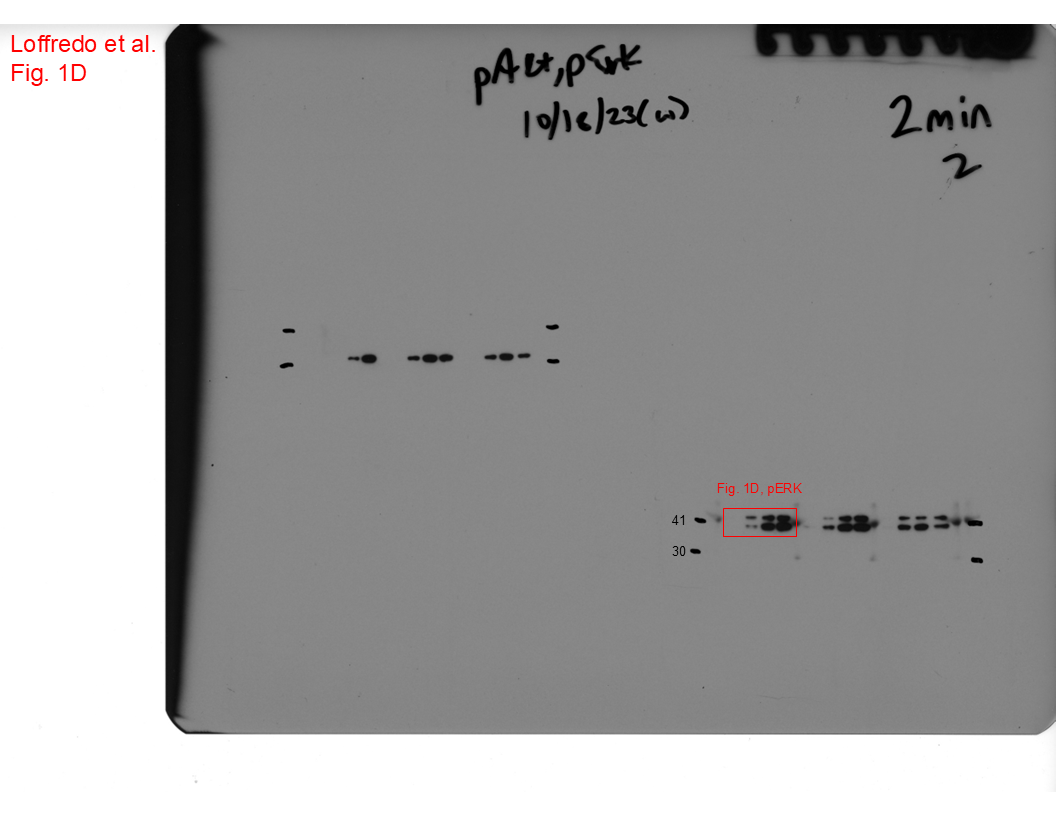

Supplement: Supplementary file 4 — Source Data [file 41467_2025_57362_MOESM4_ESM.zip › SourceData/Western_Fig1D_pERK.tif]

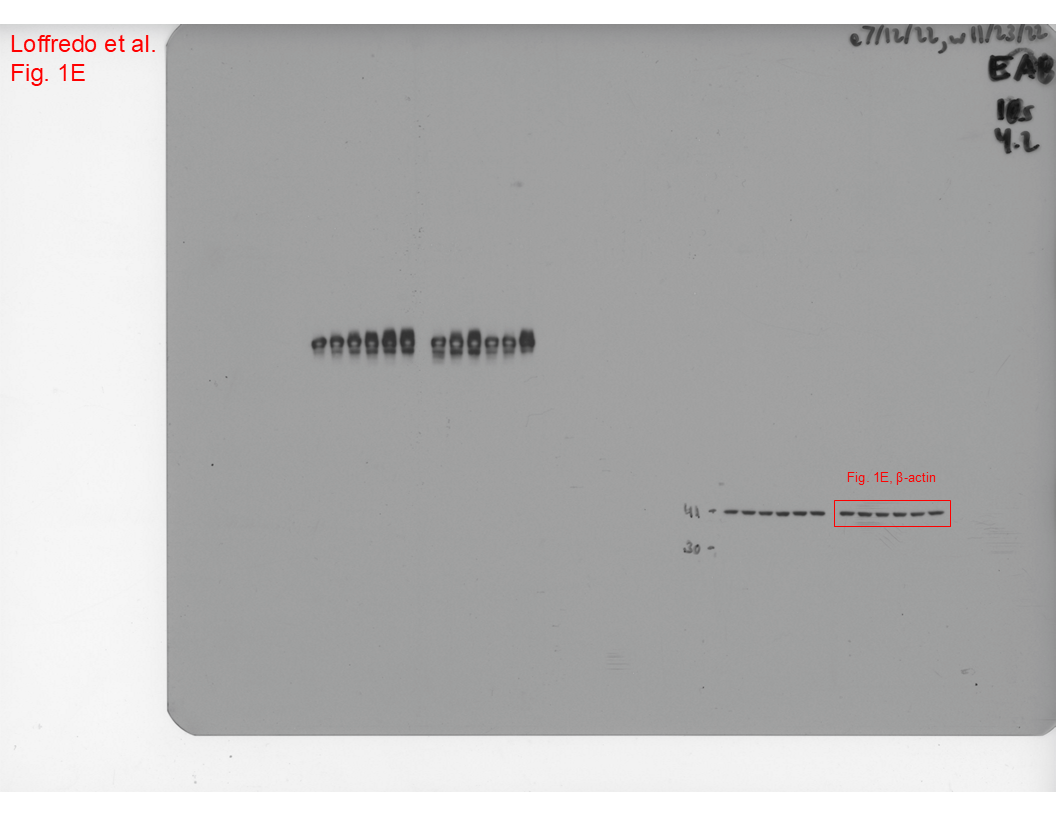

Supplement: Supplementary file 4 — Source Data [file 41467_2025_57362_MOESM4_ESM.zip › SourceData/Western_Fig1E_bActin.tif]

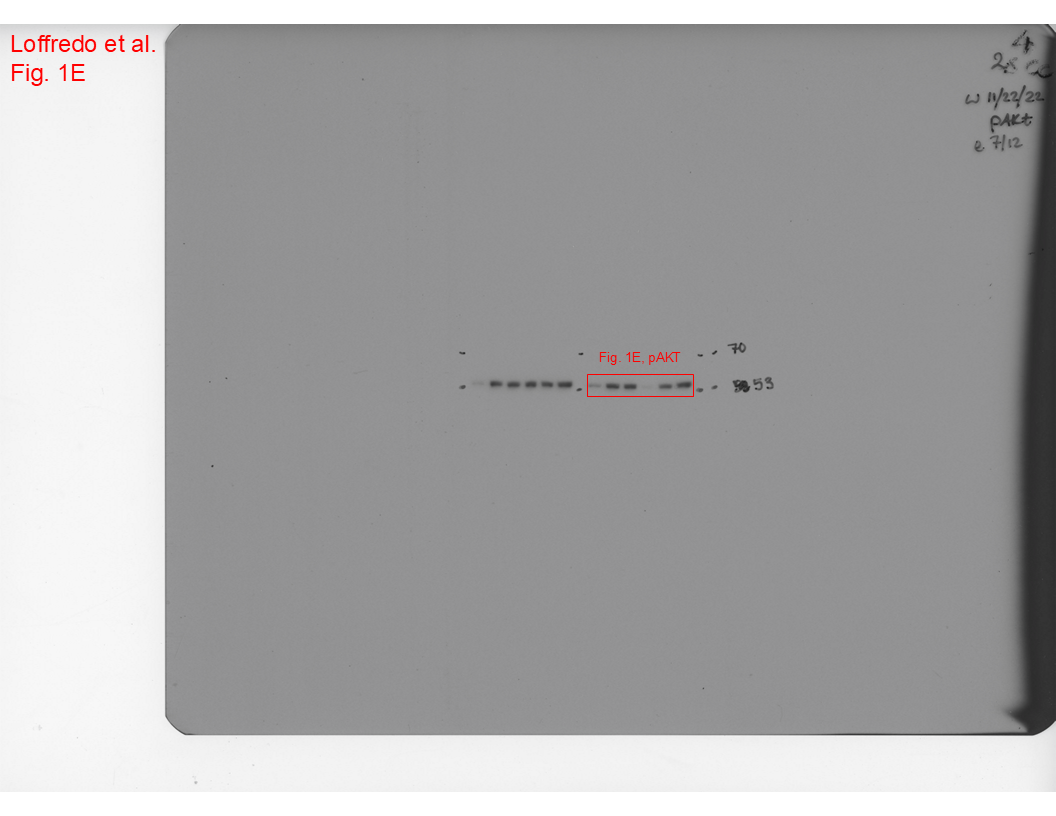

Supplement: Supplementary file 4 — Source Data [file 41467_2025_57362_MOESM4_ESM.zip › SourceData/Western_Fig1E_pAKT.tif]

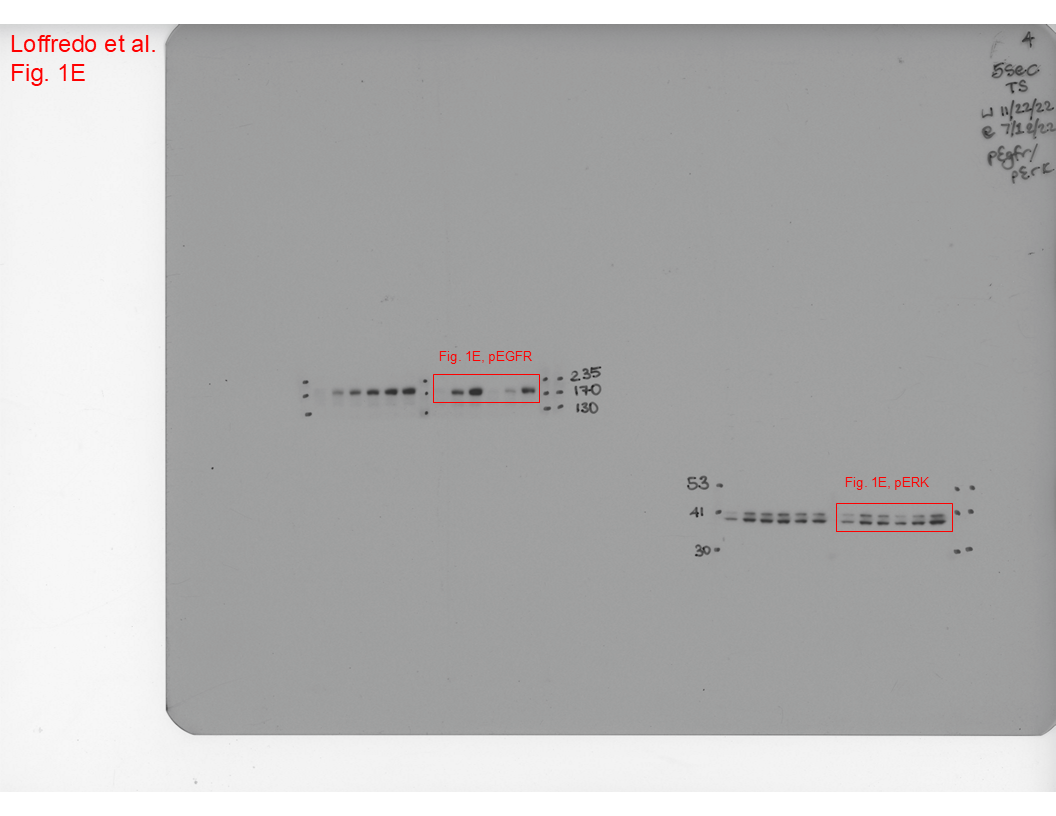

Supplement: Supplementary file 4 — Source Data [file 41467_2025_57362_MOESM4_ESM.zip › SourceData/Western_Fig1E_pEGFR_pERK.tif]

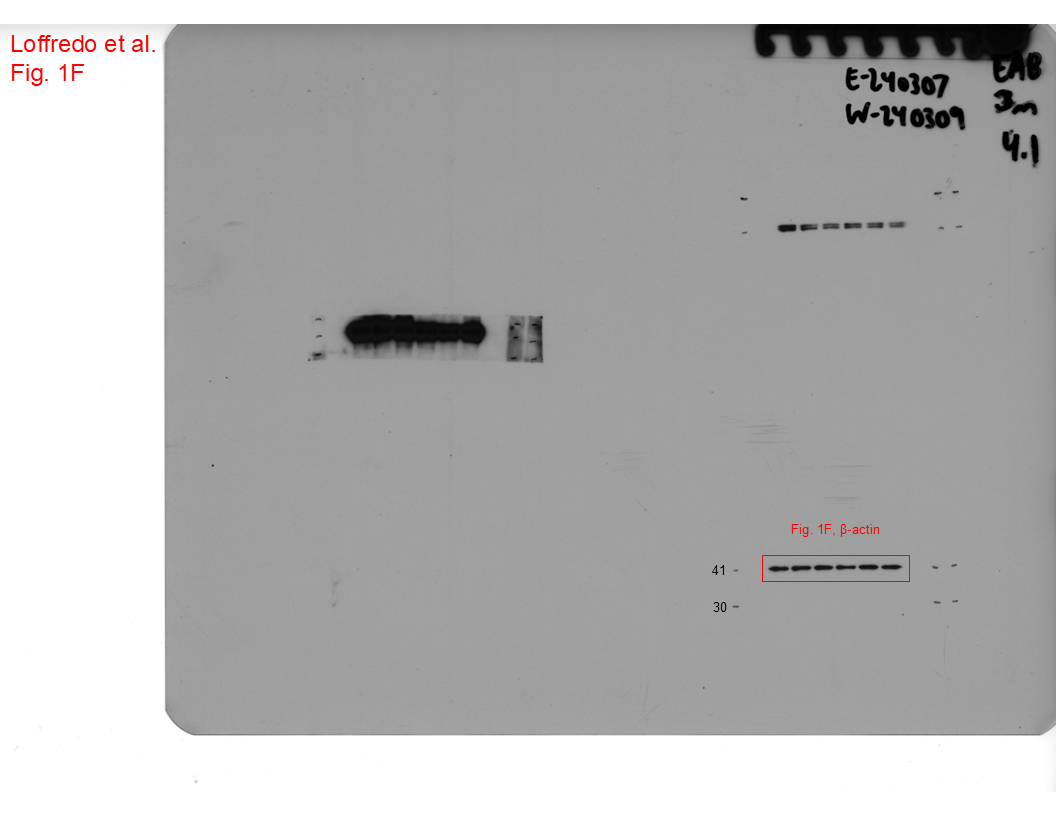

Supplement: Supplementary file 4 — Source Data [file 41467_2025_57362_MOESM4_ESM.zip › SourceData/Western_Fig1F_bActin.tif]

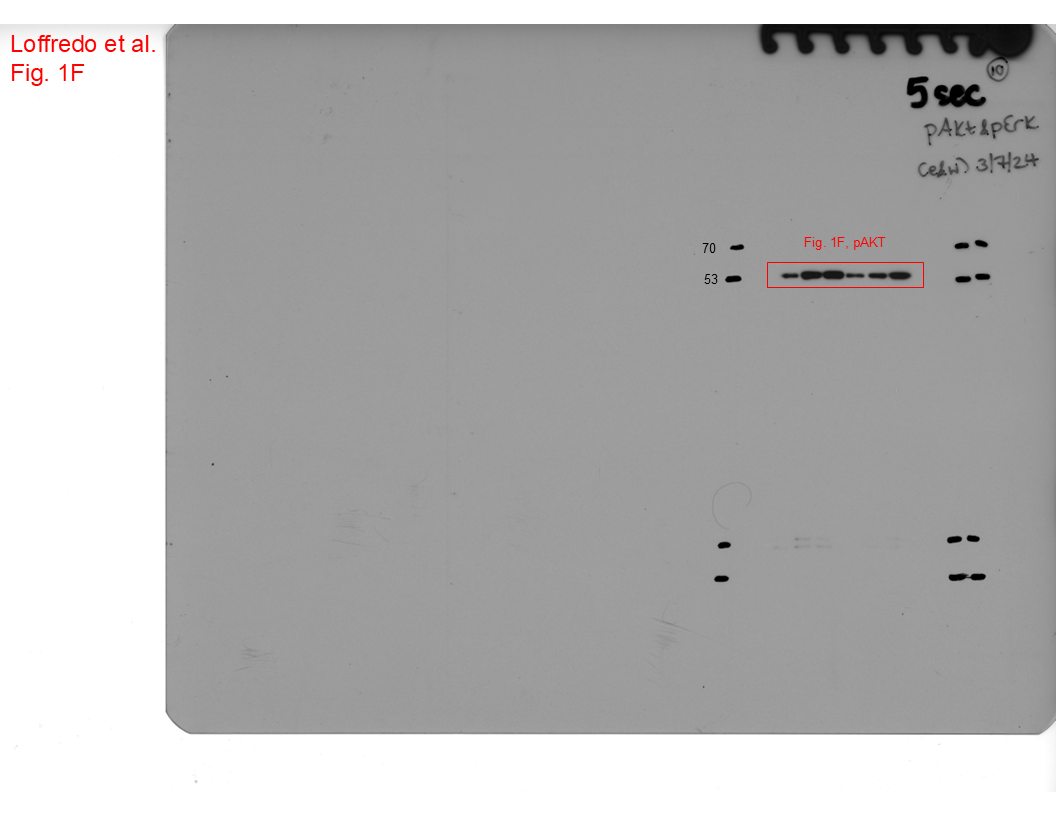

Supplement: Supplementary file 4 — Source Data [file 41467_2025_57362_MOESM4_ESM.zip › SourceData/Western_Fig1F_pAKT.tif]

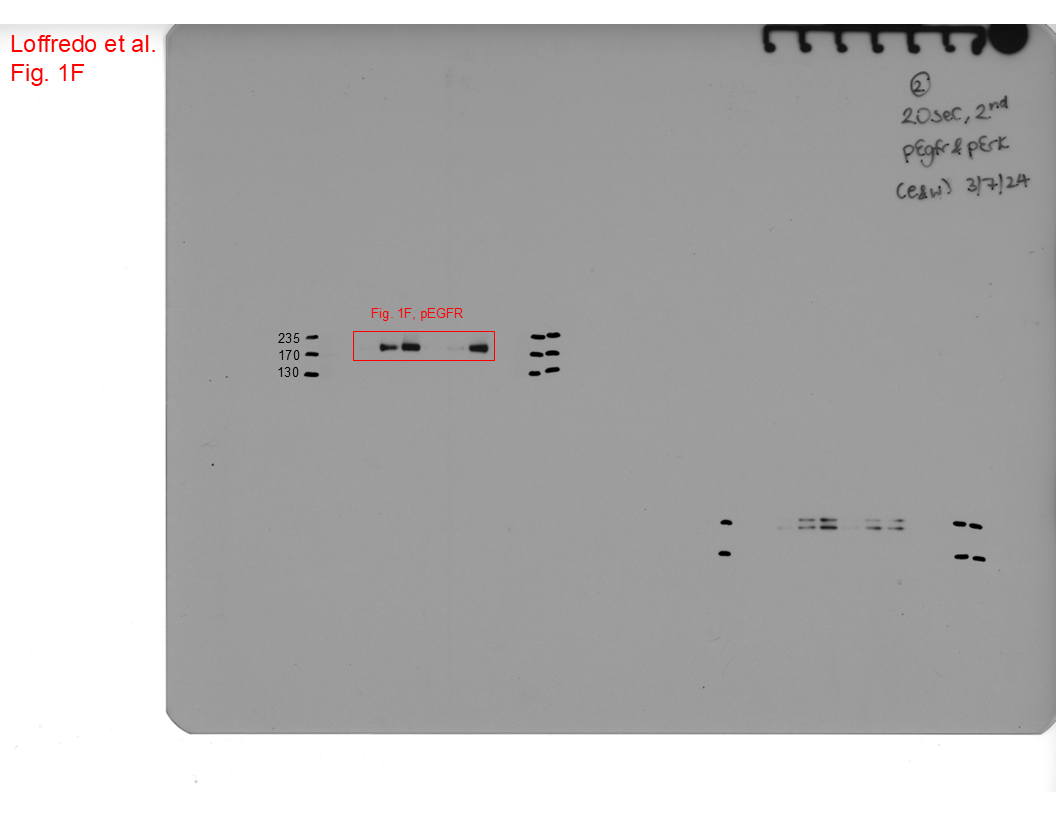

Supplement: Supplementary file 4 — Source Data [file 41467_2025_57362_MOESM4_ESM.zip › SourceData/Western_Fig1F_pEGFR.tif]

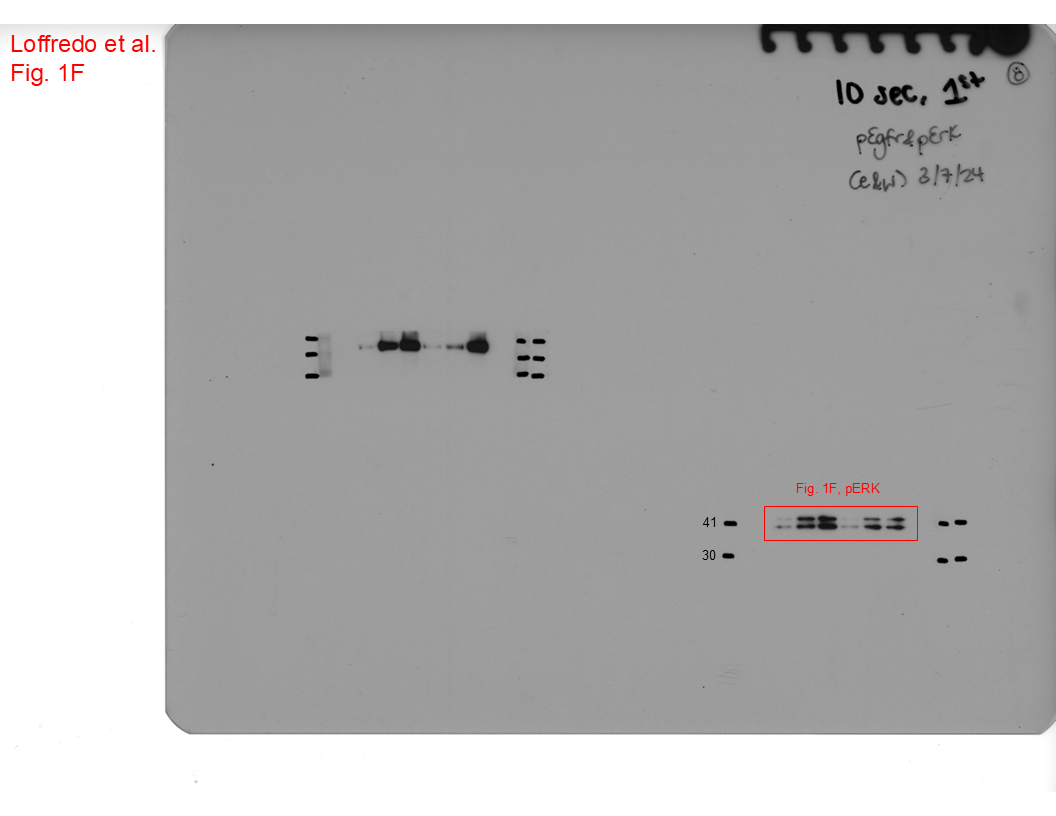

Supplement: Supplementary file 4 — Source Data [file 41467_2025_57362_MOESM4_ESM.zip › SourceData/Western_Fig1F_pERK.tif]

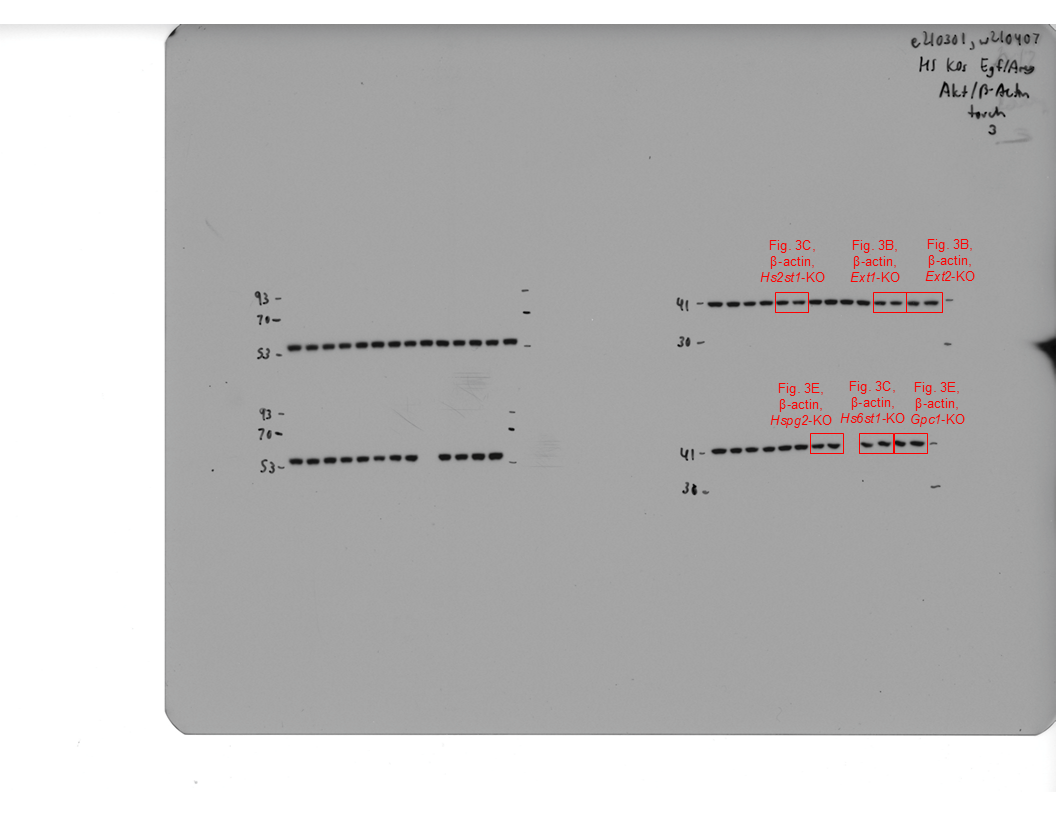

Supplement: Supplementary file 4 — Source Data [file 41467_2025_57362_MOESM4_ESM.zip › SourceData/Western_Fig3B_3C_3E_bActin_Ext1_Ext2_Hs2st1_Hs6st1_Gpc1_Hspg2.tif]

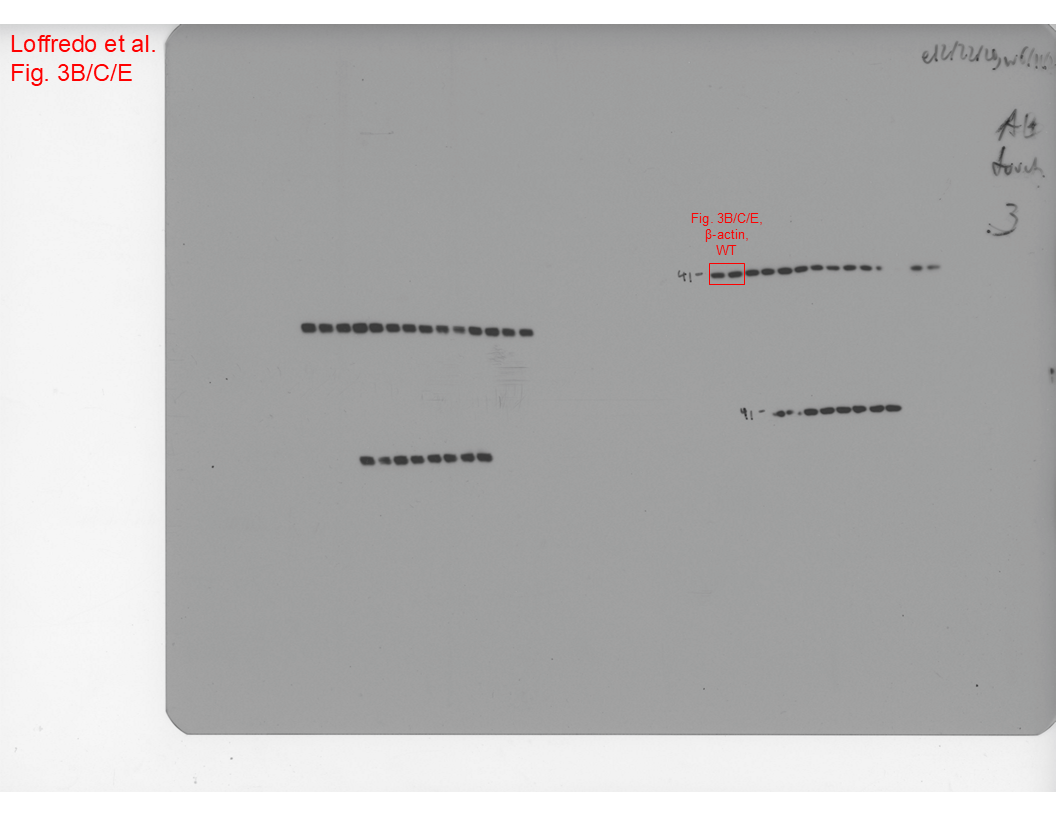

Supplement: Supplementary file 4 — Source Data [file 41467_2025_57362_MOESM4_ESM.zip › SourceData/Western_Fig3B_3C_3E_bActin_WT.tif]

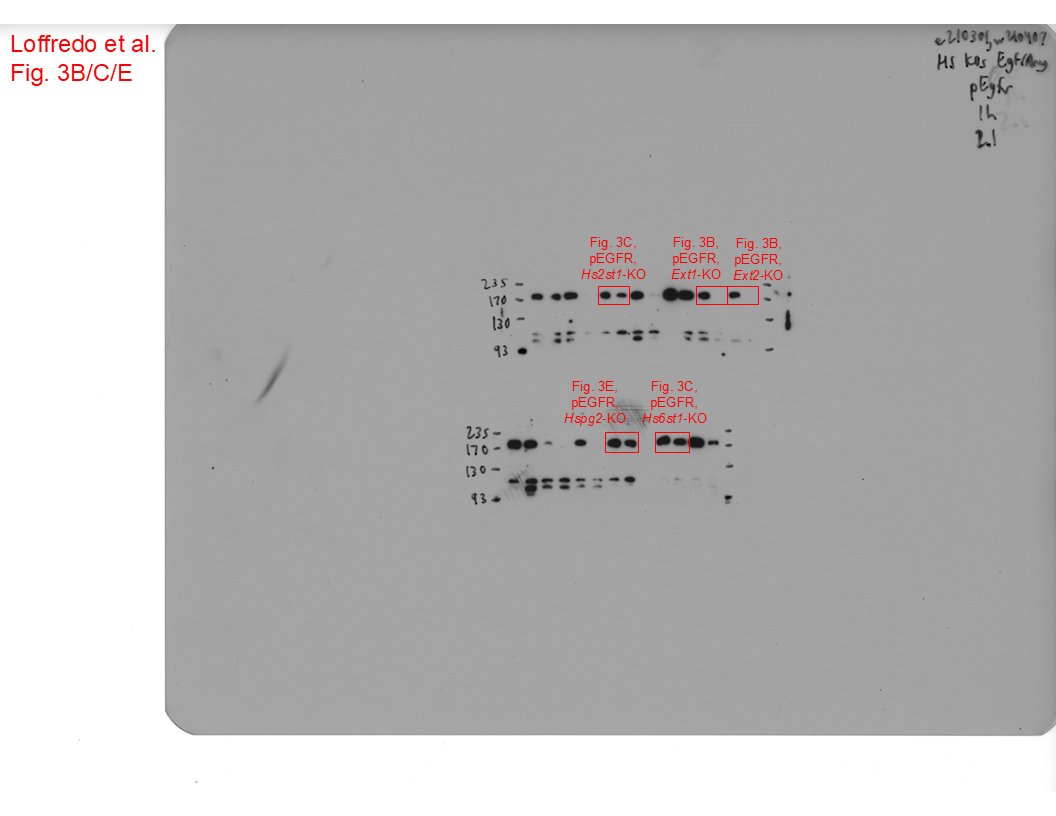

Supplement: Supplementary file 4 — Source Data [file 41467_2025_57362_MOESM4_ESM.zip › SourceData/Western_Fig3B_3C_3E_pEGFR_Ext1_Ext2_Hs2st1_Hs6st1_Hspg2.tif]

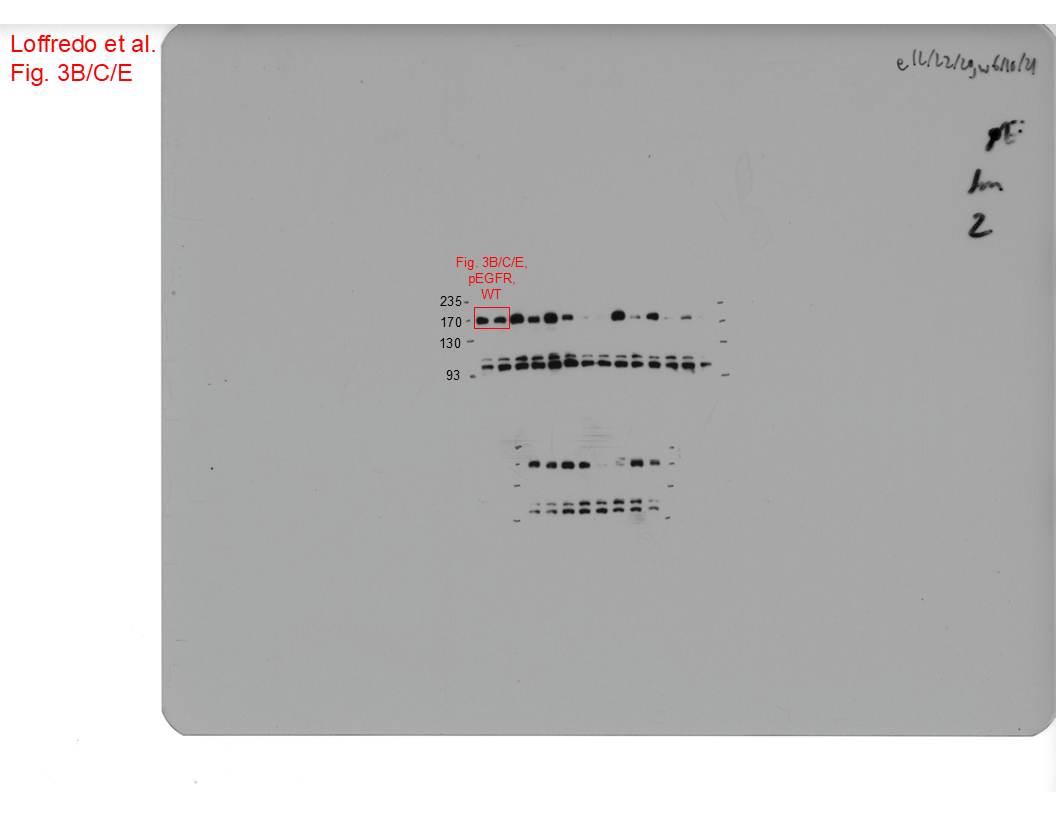

Supplement: Supplementary file 4 — Source Data [file 41467_2025_57362_MOESM4_ESM.zip › SourceData/Western_Fig3B_3C_3E_pEGFR_WT.tif]

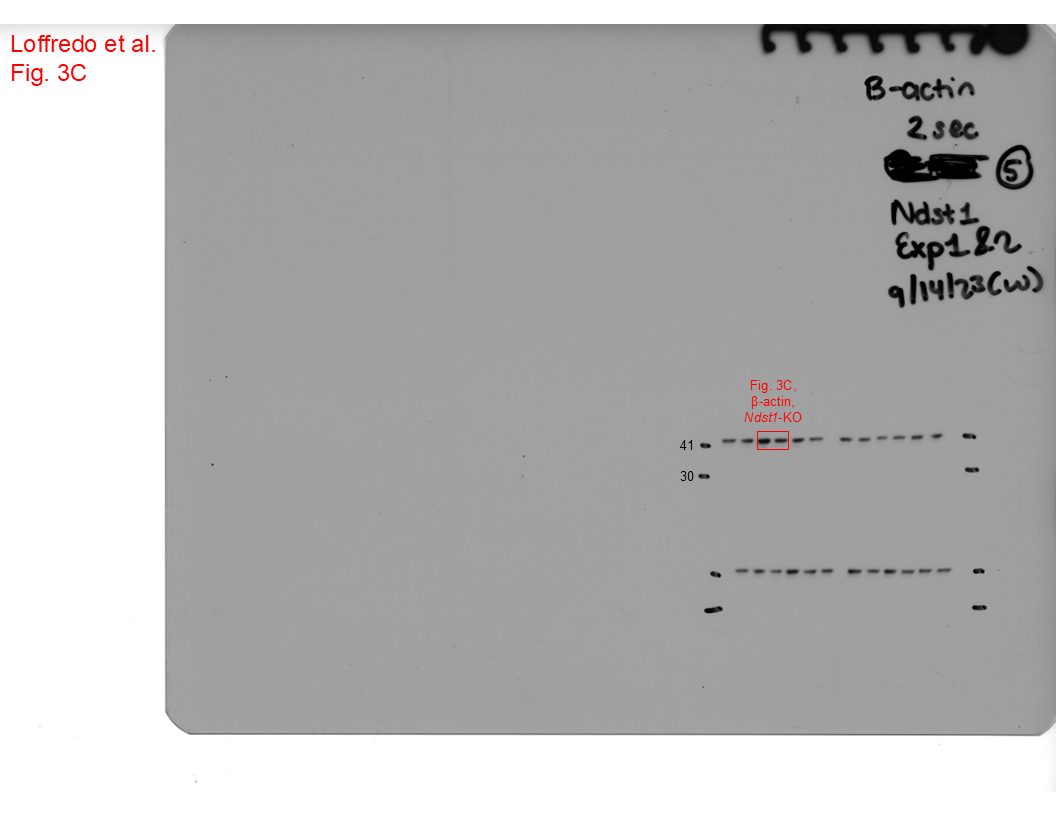

Supplement: Supplementary file 4 — Source Data [file 41467_2025_57362_MOESM4_ESM.zip › SourceData/Western_Fig3C_bActin_Ndst1.tif]

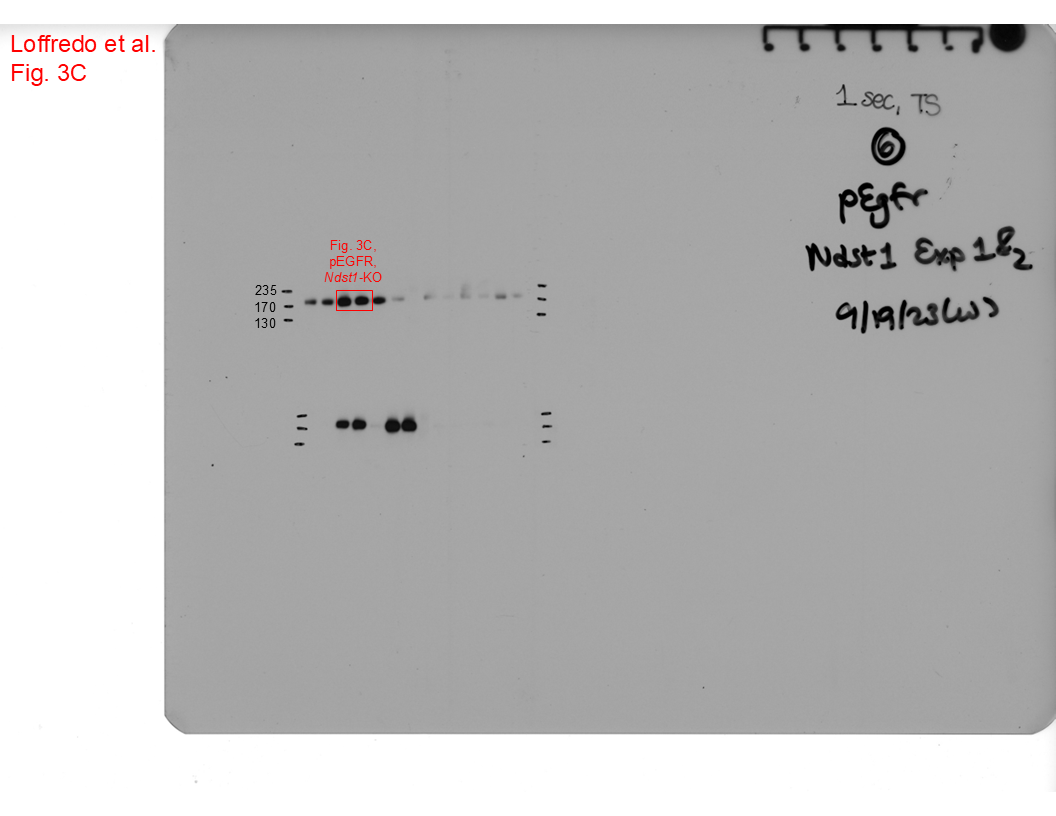

Supplement: Supplementary file 4 — Source Data [file 41467_2025_57362_MOESM4_ESM.zip › SourceData/Western_Fig3C_pEGFR_Ndst1.tif]

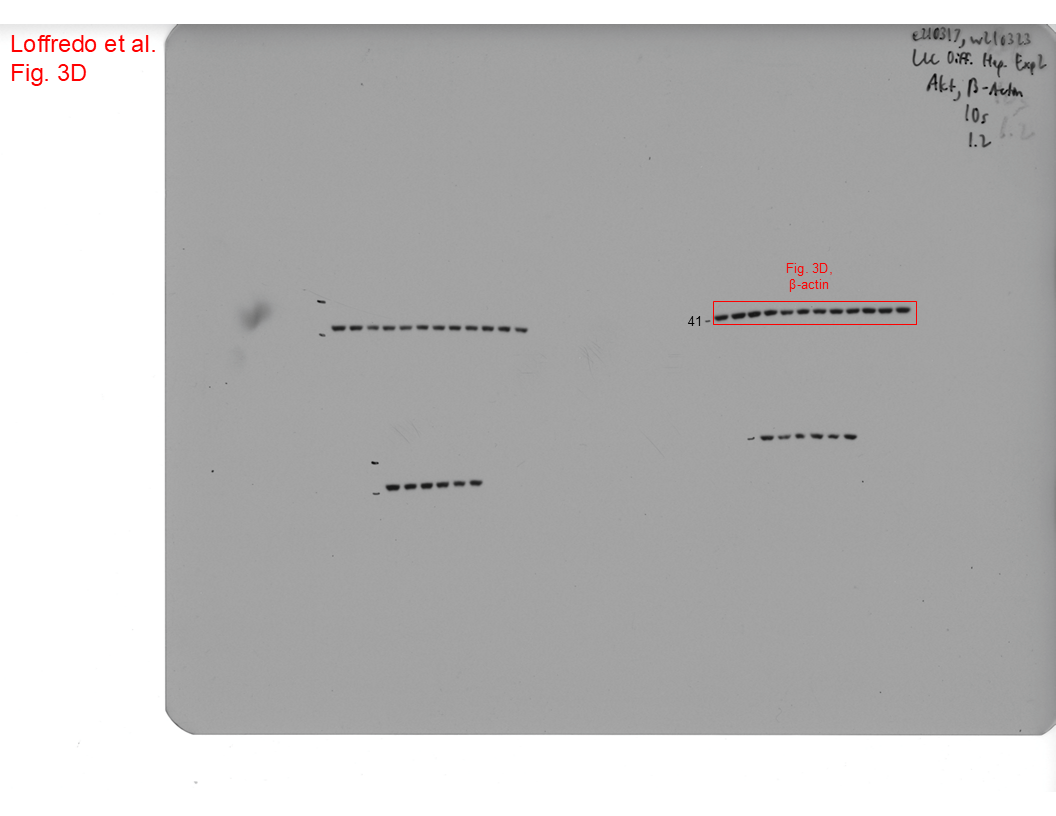

Supplement: Supplementary file 4 — Source Data [file 41467_2025_57362_MOESM4_ESM.zip › SourceData/Western_Fig3D_bActin.tif]

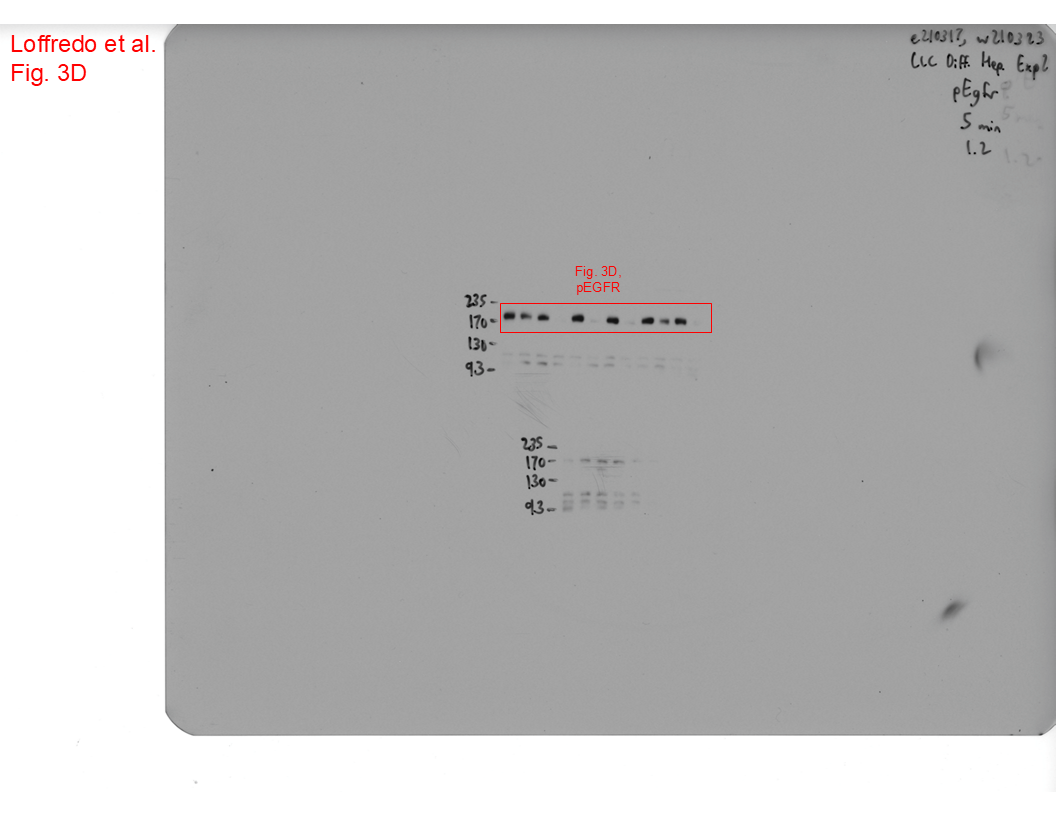

Supplement: Supplementary file 4 — Source Data [file 41467_2025_57362_MOESM4_ESM.zip › SourceData/Western_Fig3D_pEGFR.tif]

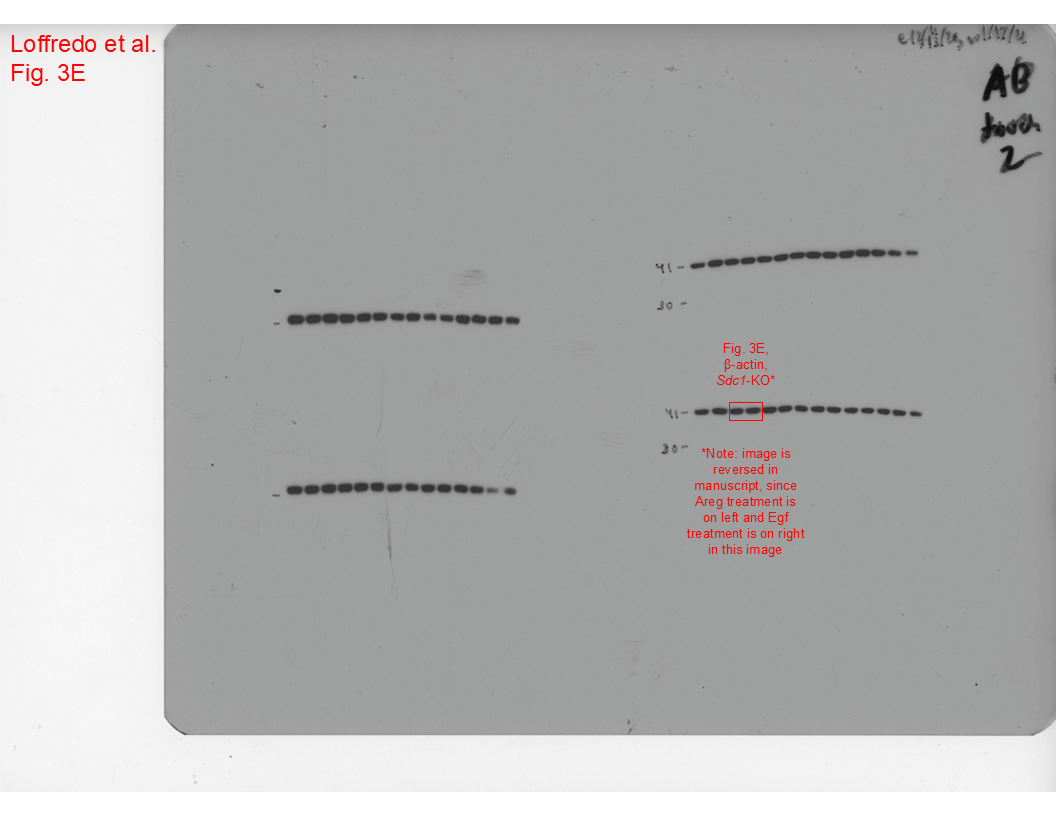

Supplement: Supplementary file 4 — Source Data [file 41467_2025_57362_MOESM4_ESM.zip › SourceData/Western_Fig3E_bActin_Sdc1.tif]

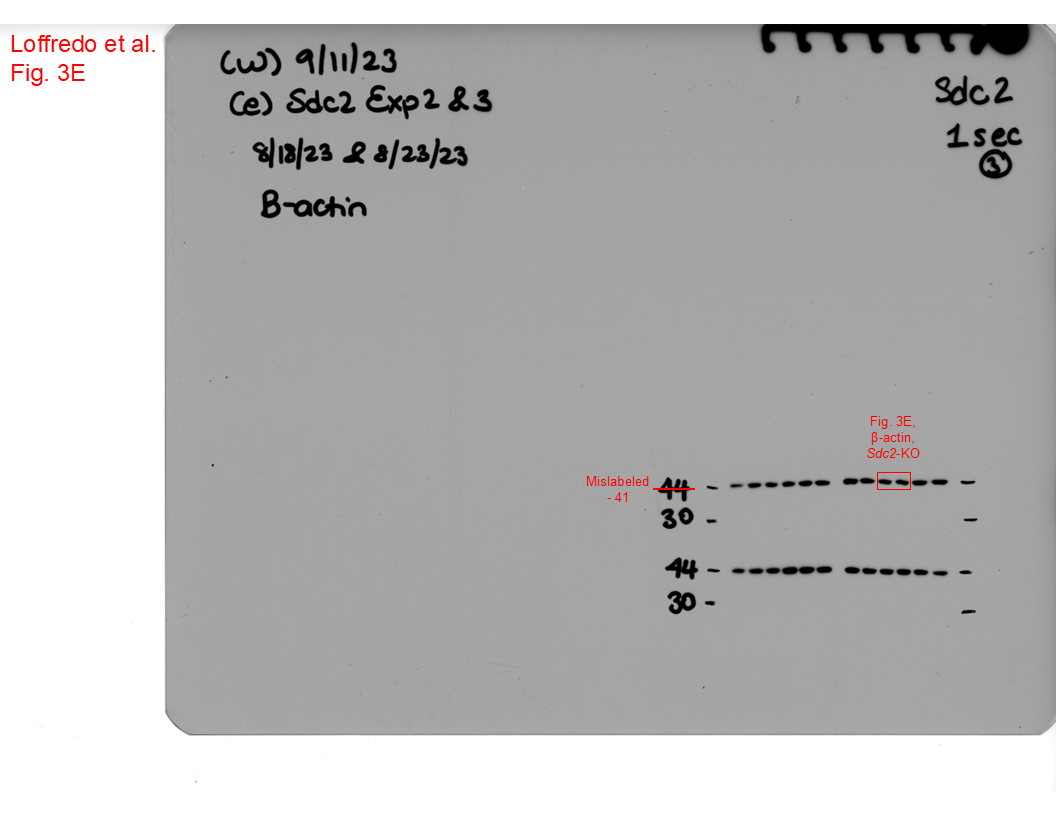

Supplement: Supplementary file 4 — Source Data [file 41467_2025_57362_MOESM4_ESM.zip › SourceData/Western_Fig3E_bActin_Sdc2.tif]

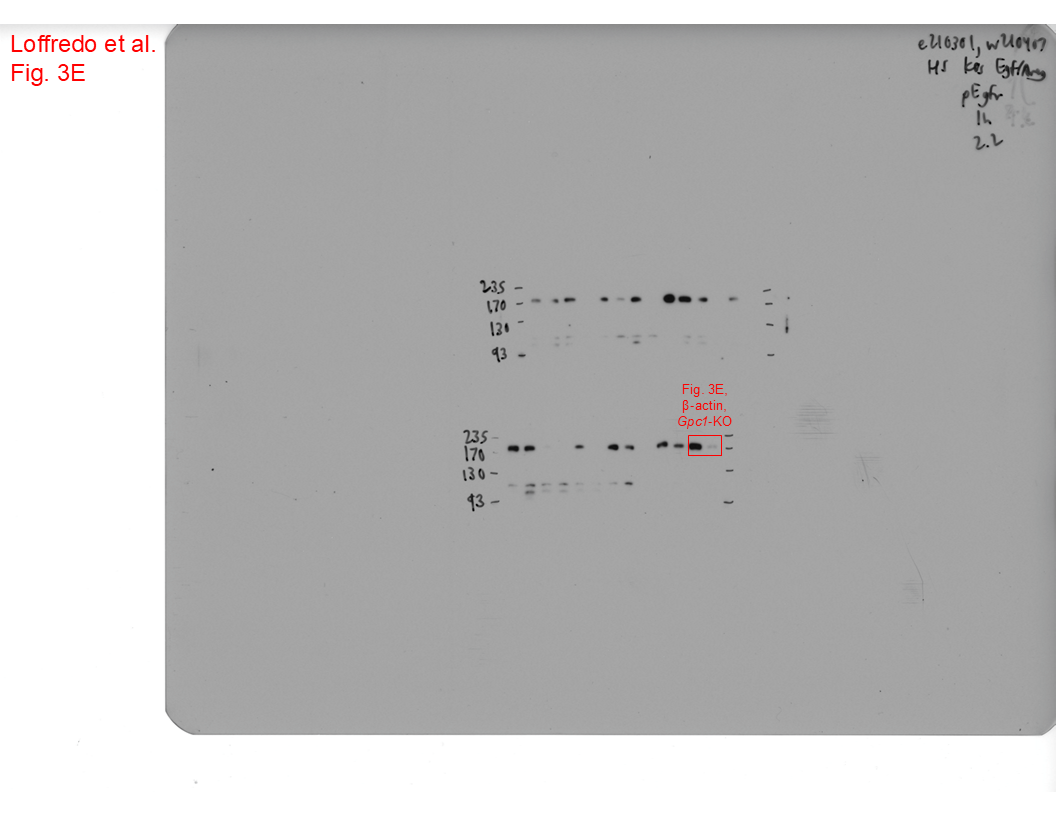

Supplement: Supplementary file 4 — Source Data [file 41467_2025_57362_MOESM4_ESM.zip › SourceData/Western_Fig3E_pEGFR_Gpc1.tif]

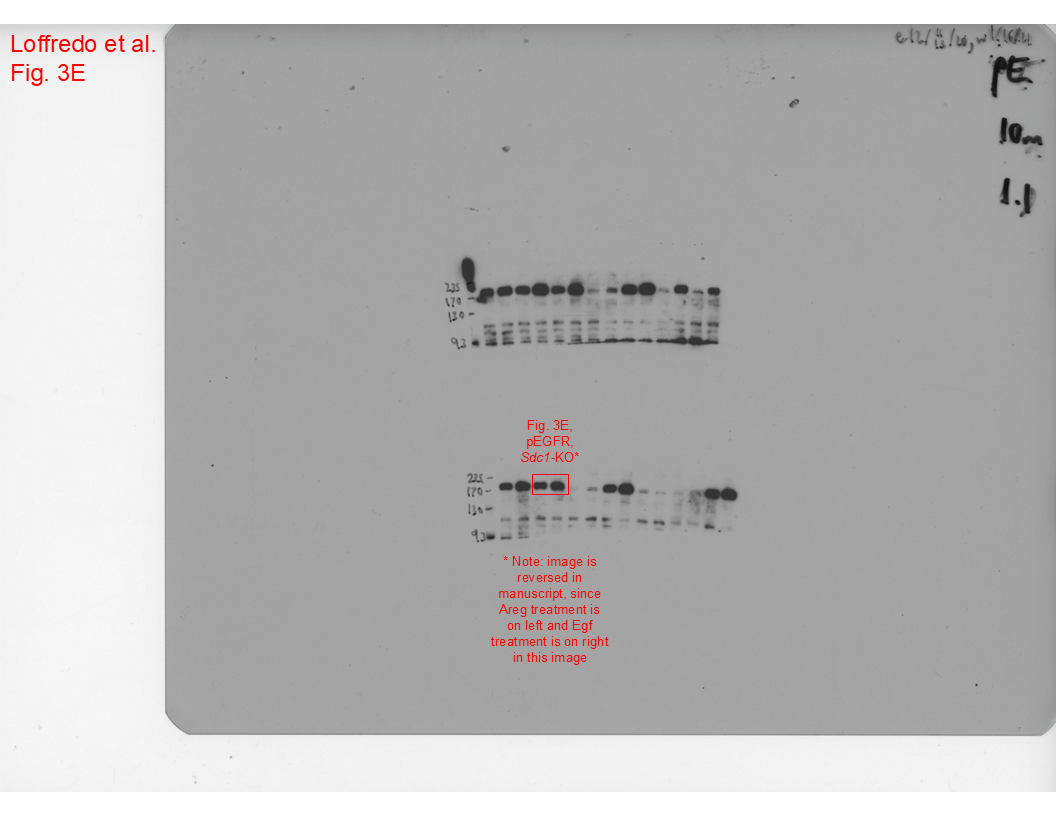

Supplement: Supplementary file 4 — Source Data [file 41467_2025_57362_MOESM4_ESM.zip › SourceData/Western_Fig3E_pEGFR_Sdc1.tif]

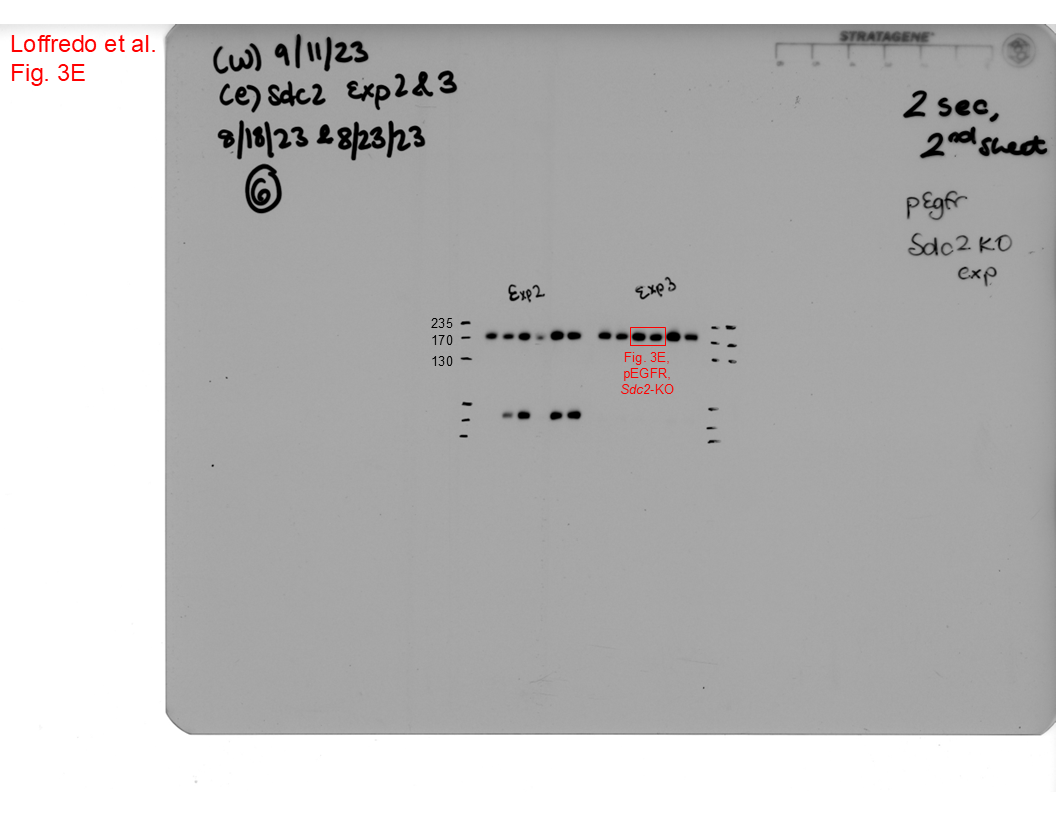

Supplement: Supplementary file 4 — Source Data [file 41467_2025_57362_MOESM4_ESM.zip › SourceData/Western_Fig3E_pEGFR_Sdc2.tif]

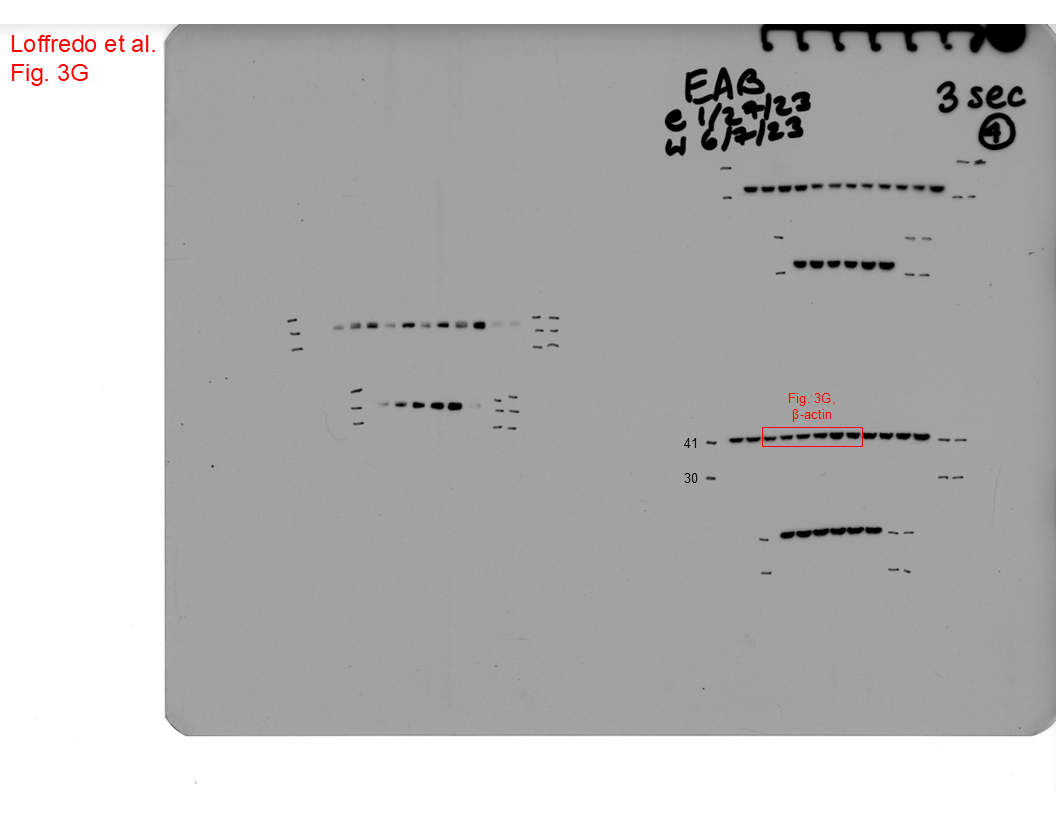

Supplement: Supplementary file 4 — Source Data [file 41467_2025_57362_MOESM4_ESM.zip › SourceData/Western_Fig3G_bActin.tif]

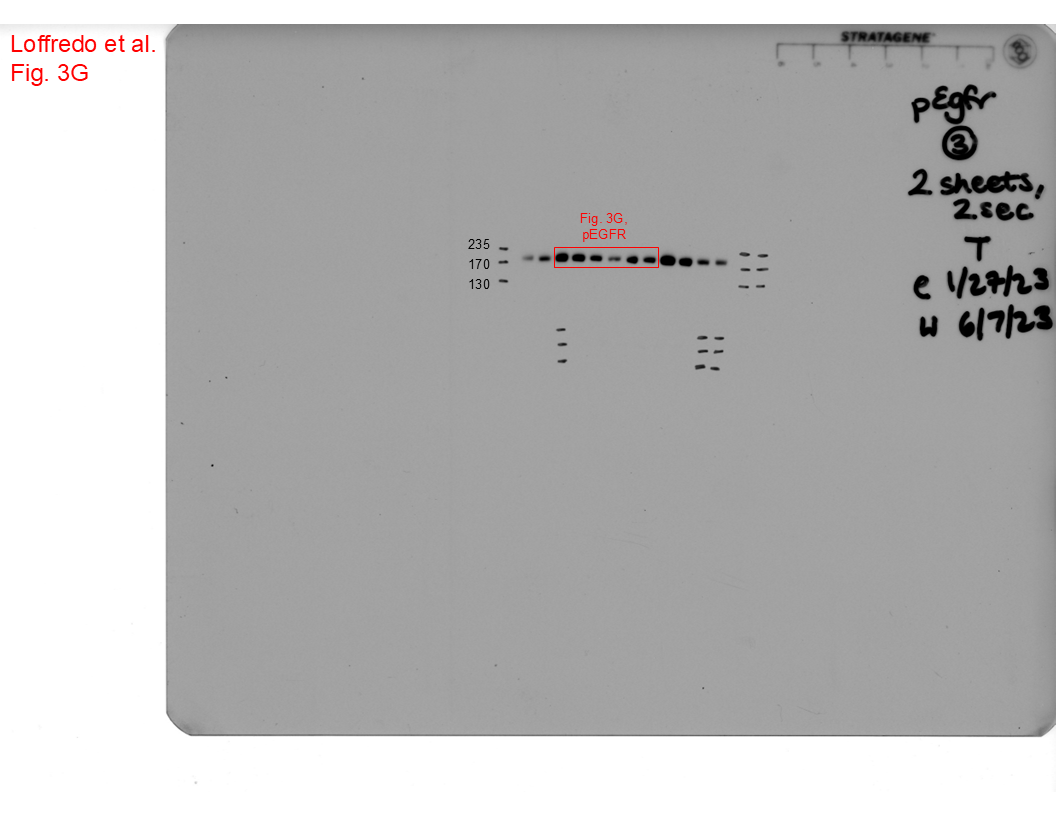

Supplement: Supplementary file 4 — Source Data [file 41467_2025_57362_MOESM4_ESM.zip › SourceData/Western_Fig3G_pEGFR.tif]

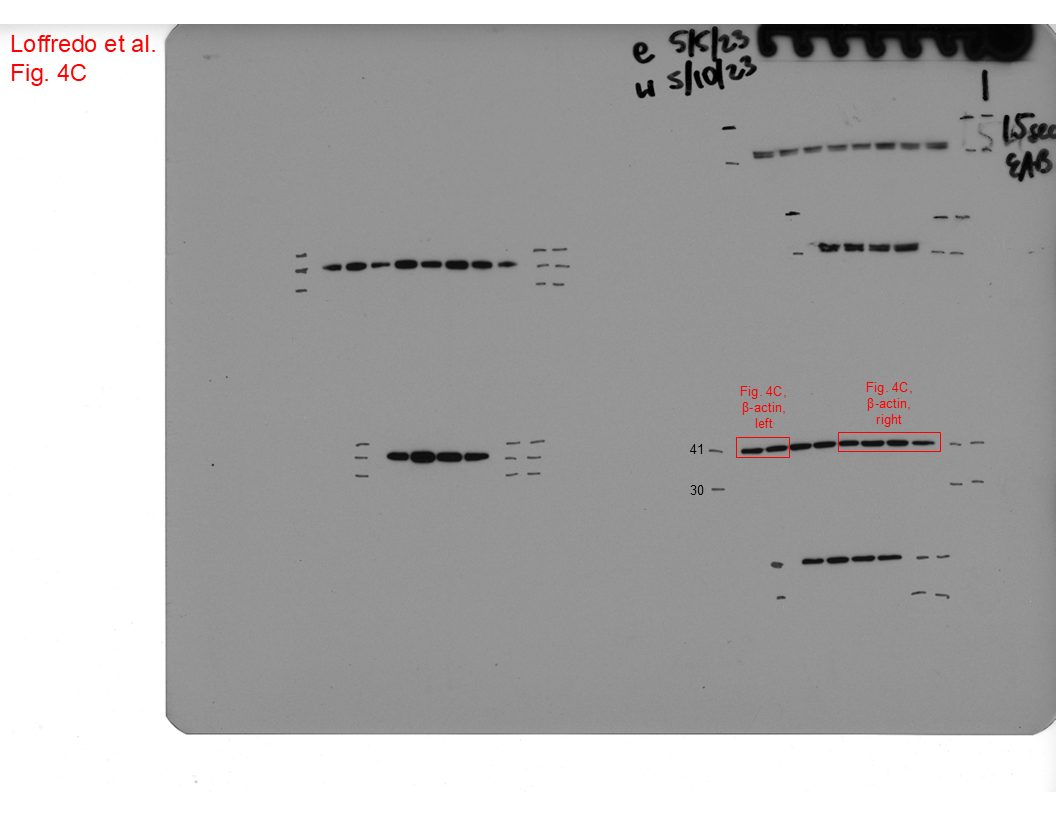

Supplement: Supplementary file 4 — Source Data [file 41467_2025_57362_MOESM4_ESM.zip › SourceData/Western_Fig4C_bActin.tif]

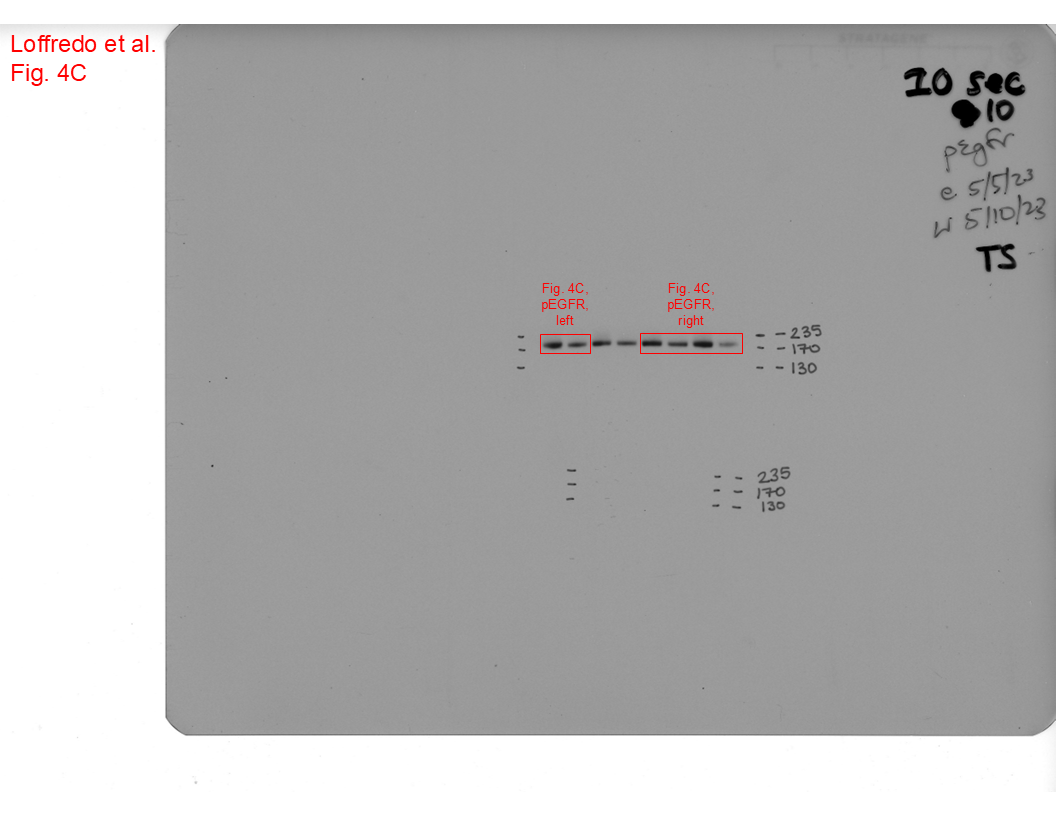

Supplement: Supplementary file 4 — Source Data [file 41467_2025_57362_MOESM4_ESM.zip › SourceData/Western_Fig4C_pEGFR.tif]

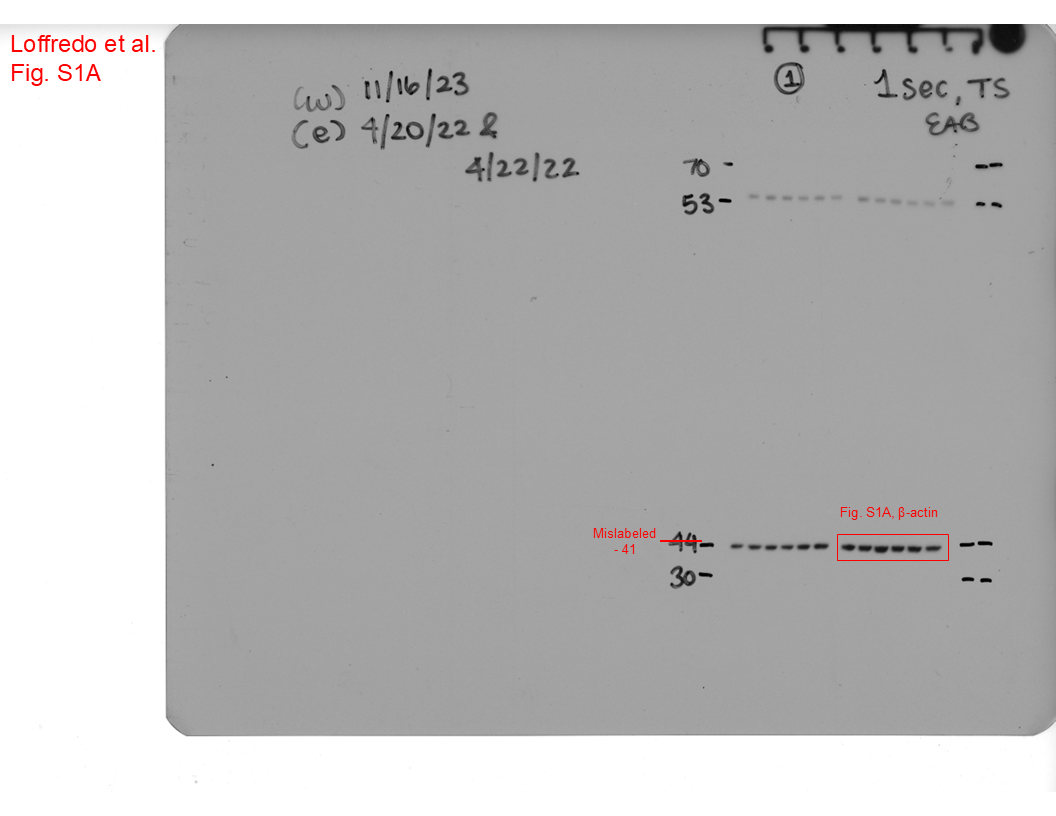

Supplement: Supplementary file 4 — Source Data [file 41467_2025_57362_MOESM4_ESM.zip › SourceData/Western_FigS1A_bActin.tif]

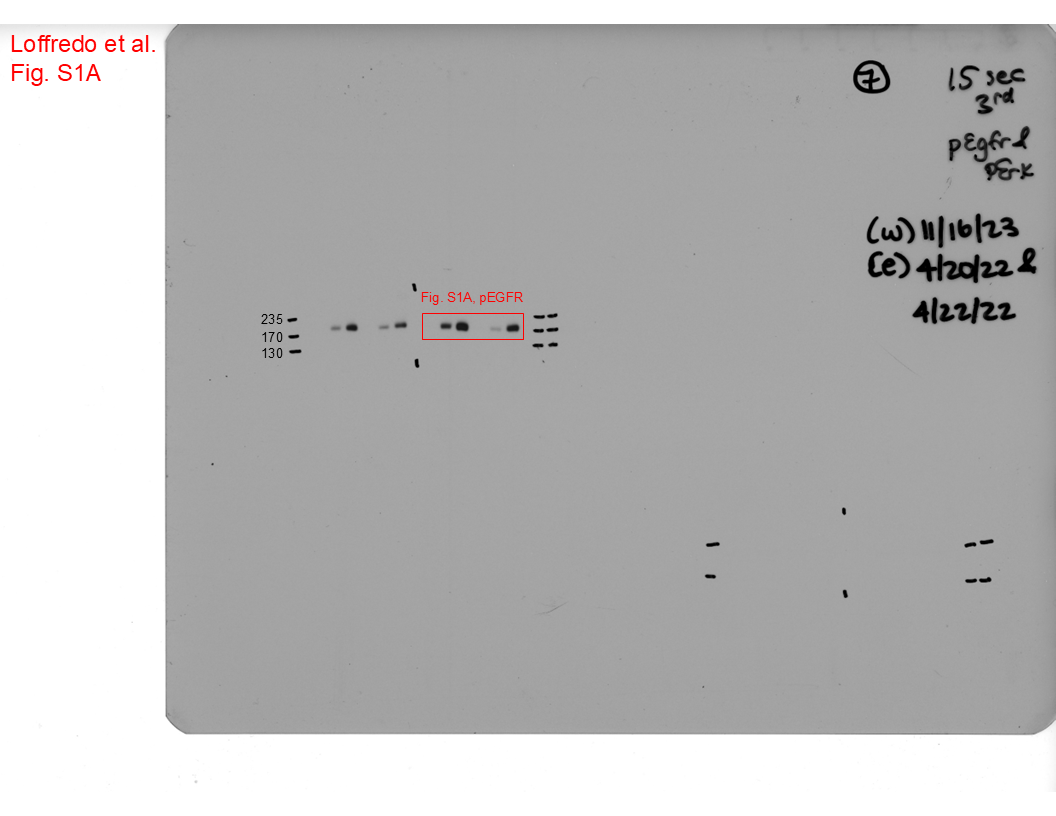

Supplement: Supplementary file 4 — Source Data [file 41467_2025_57362_MOESM4_ESM.zip › SourceData/Western_FigS1A_pEGFR.tif]

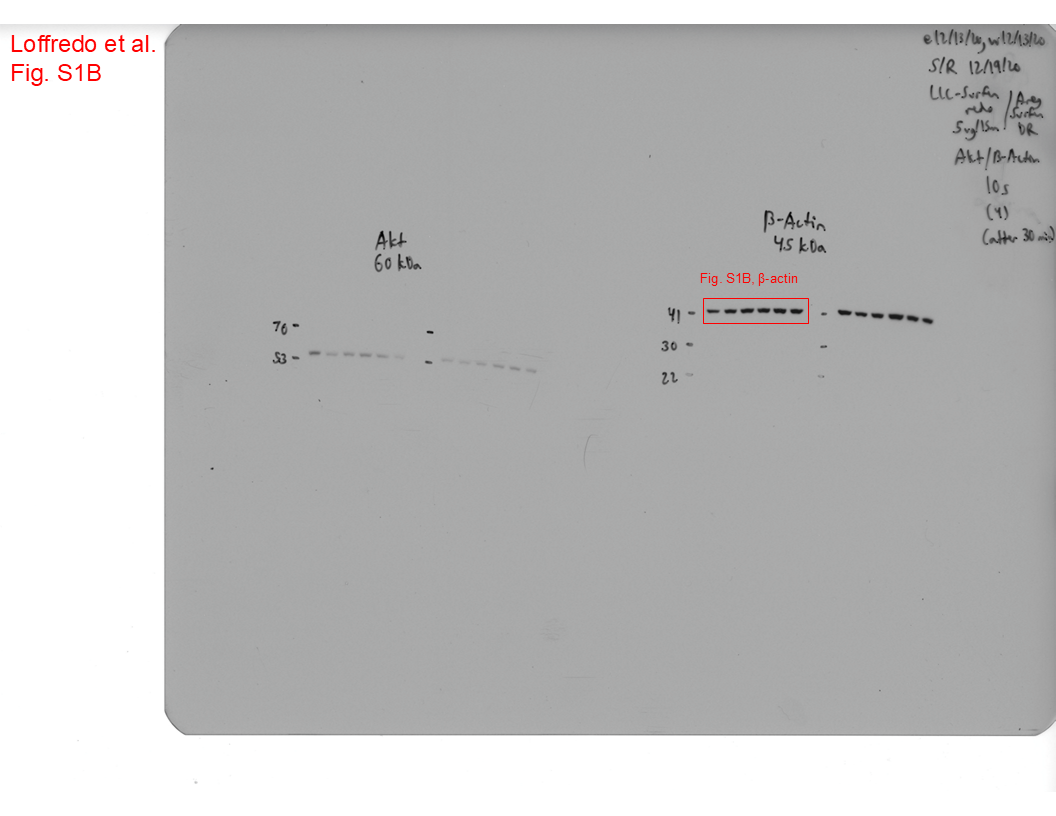

Supplement: Supplementary file 4 — Source Data [file 41467_2025_57362_MOESM4_ESM.zip › SourceData/Western_FigS1B_bActin.tif]

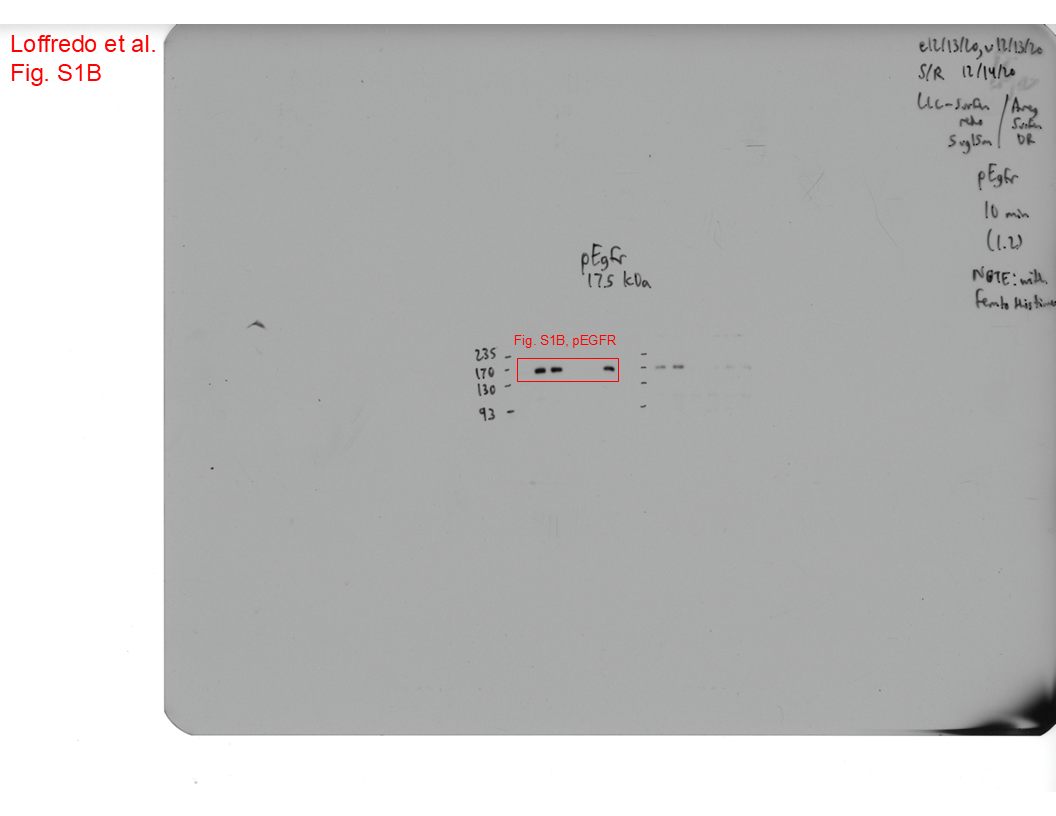

Supplement: Supplementary file 4 — Source Data [file 41467_2025_57362_MOESM4_ESM.zip › SourceData/Western_FigS1B_pEGFR.tif]

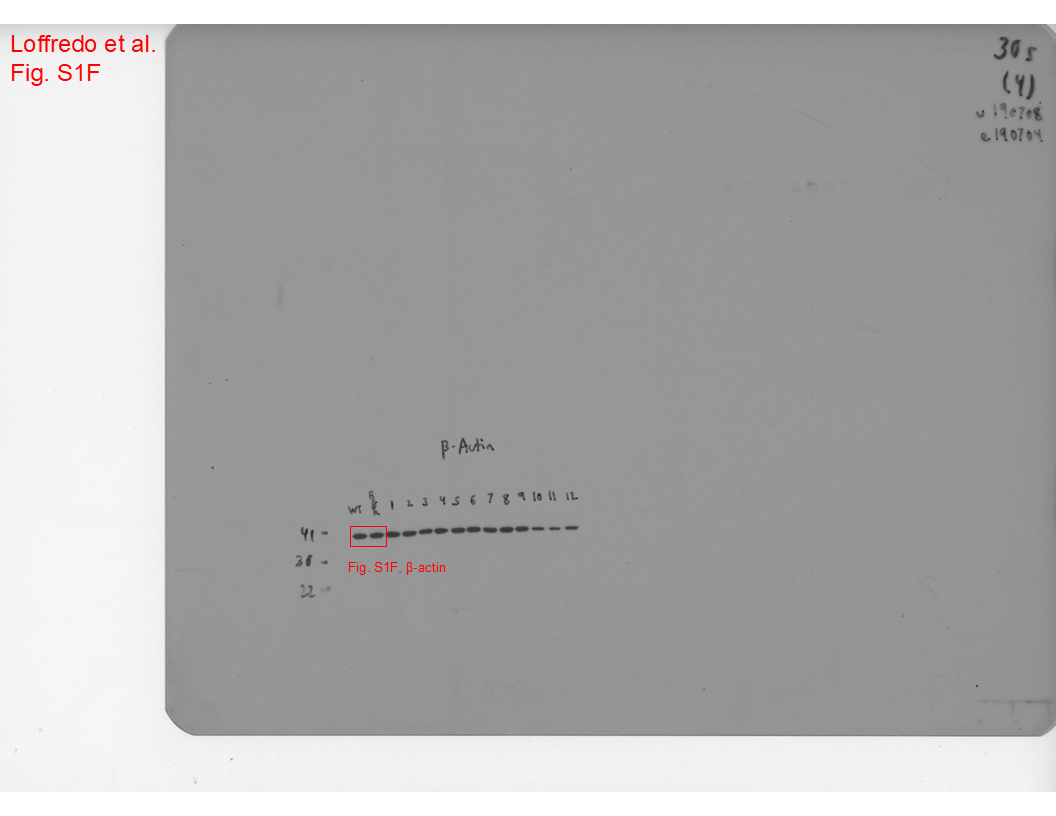

Supplement: Supplementary file 4 — Source Data [file 41467_2025_57362_MOESM4_ESM.zip › SourceData/Western_FigS1F_bActin.tif]

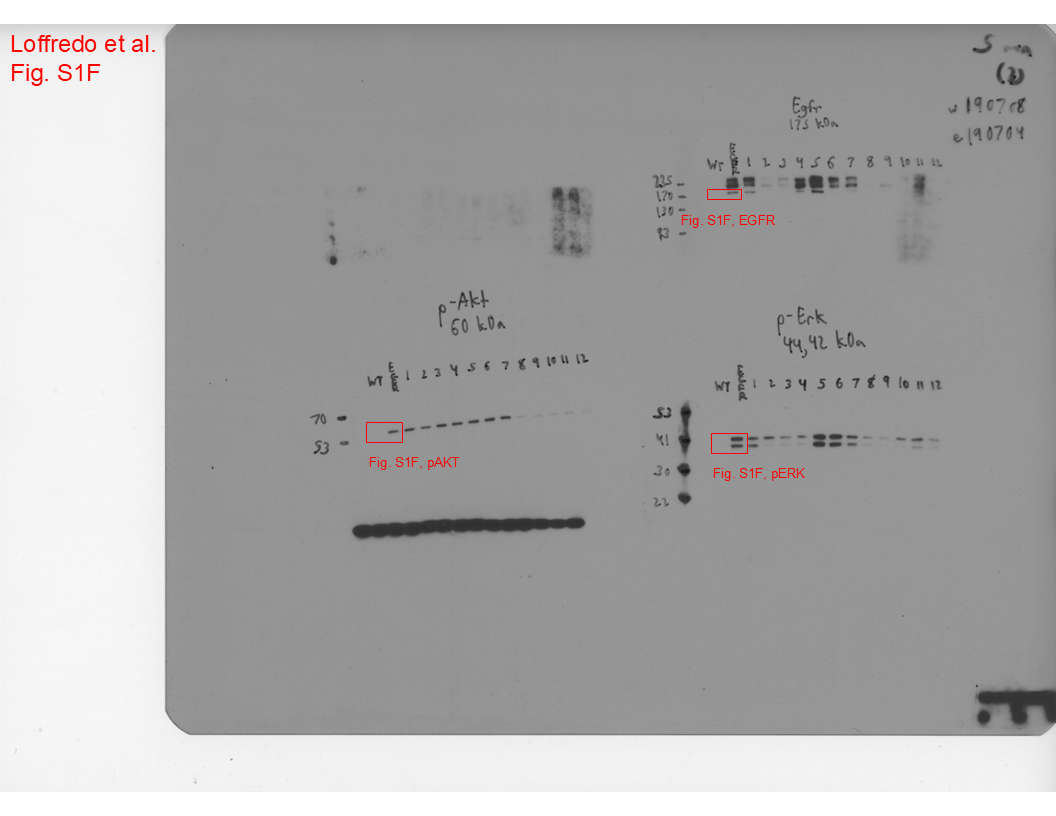

Supplement: Supplementary file 4 — Source Data [file 41467_2025_57362_MOESM4_ESM.zip › SourceData/Western_FigS1F_EGFR_pAKT_pERK.tif]

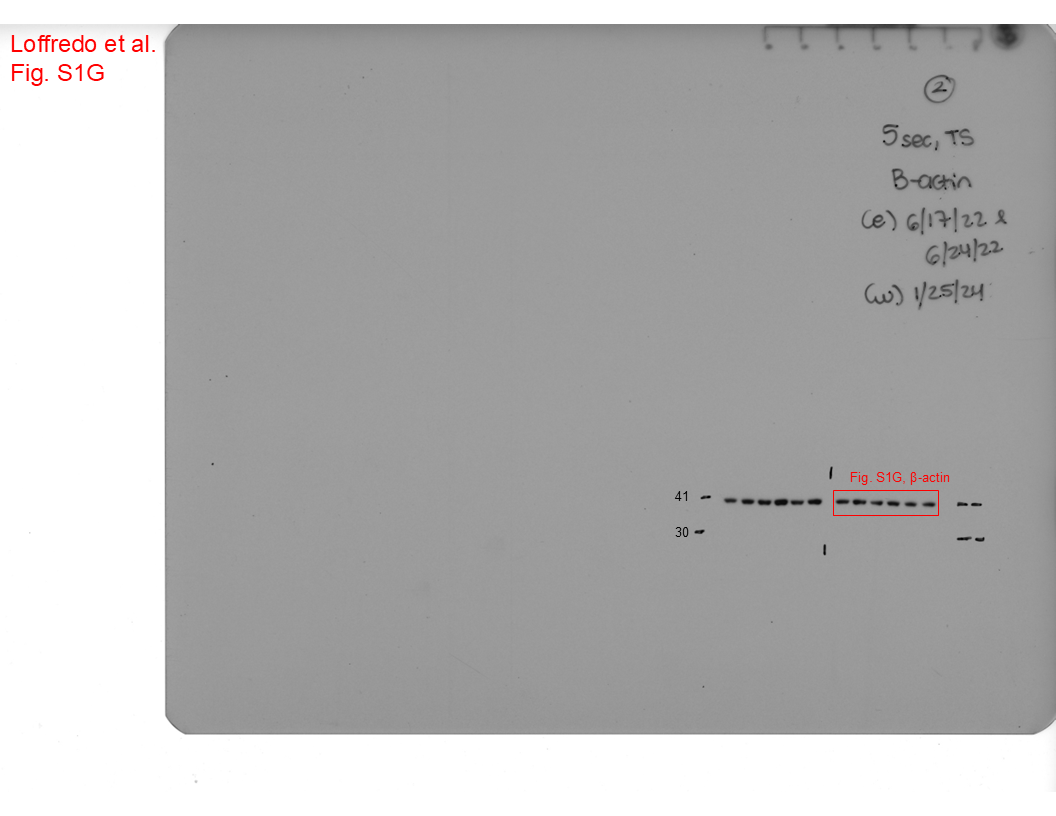

Supplement: Supplementary file 4 — Source Data [file 41467_2025_57362_MOESM4_ESM.zip › SourceData/Western_FigS1G_bActin.tif]

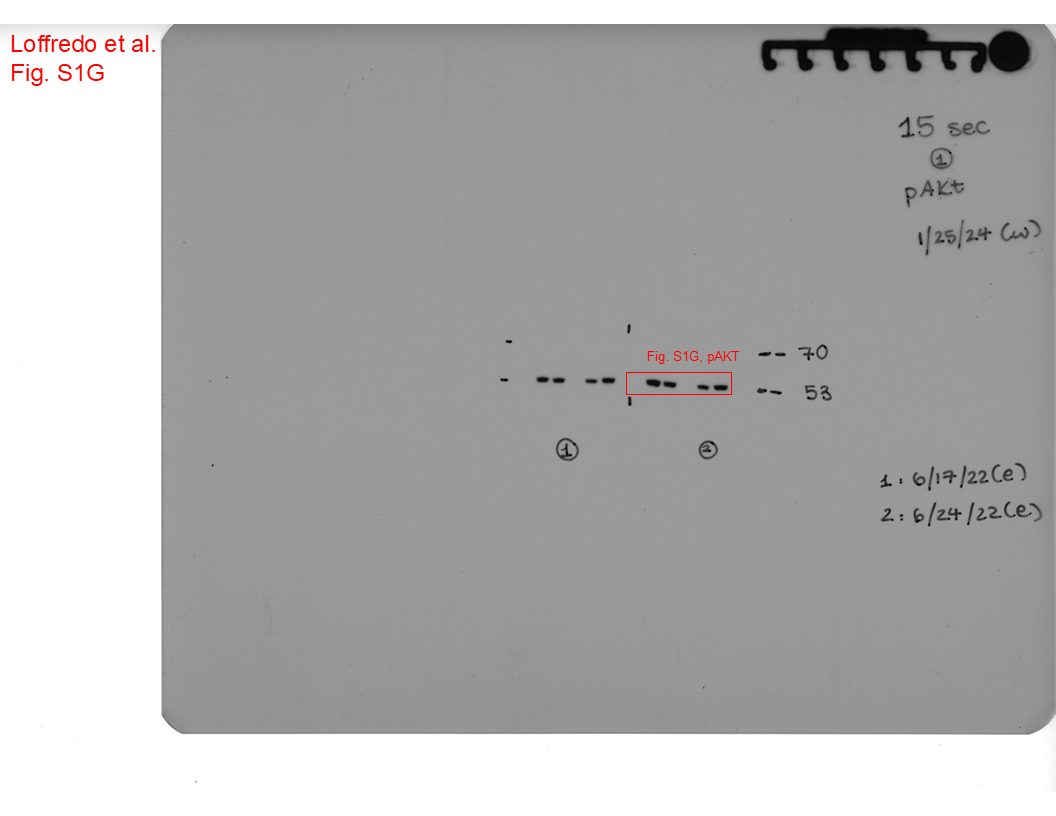

Supplement: Supplementary file 4 — Source Data [file 41467_2025_57362_MOESM4_ESM.zip › SourceData/Western_FigS1G_pAKT.tif]

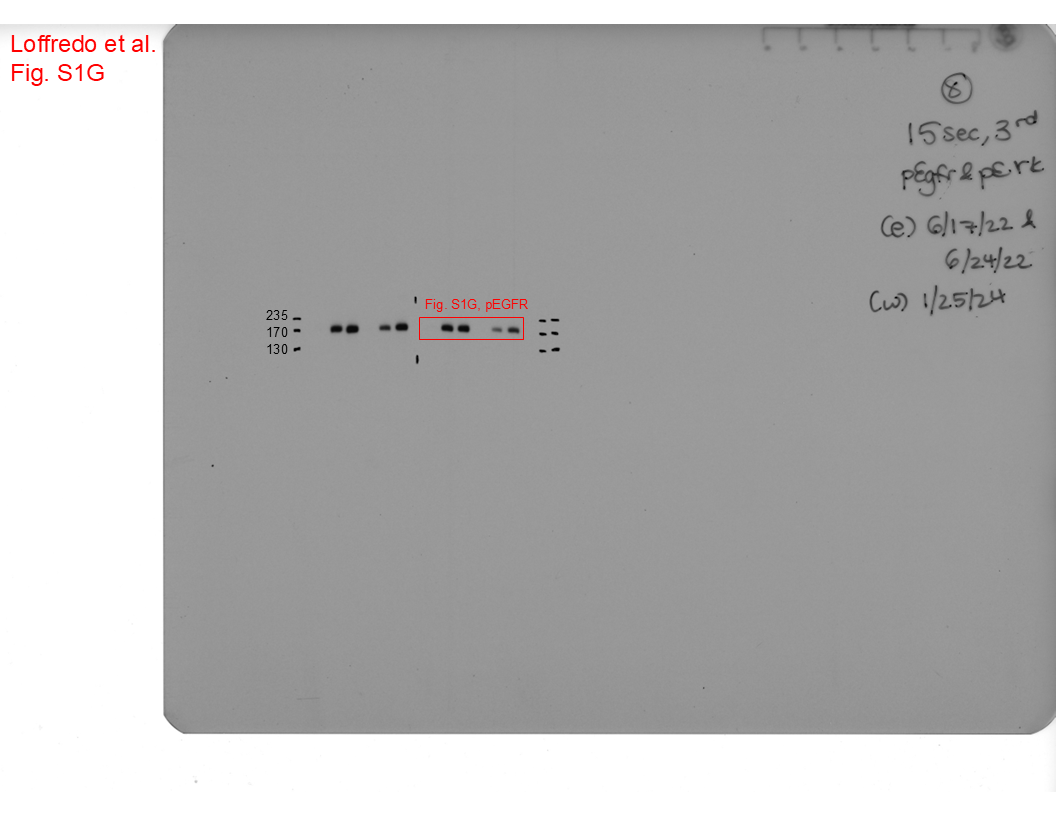

Supplement: Supplementary file 4 — Source Data [file 41467_2025_57362_MOESM4_ESM.zip › SourceData/Western_FigS1G_pEGFR.tif]

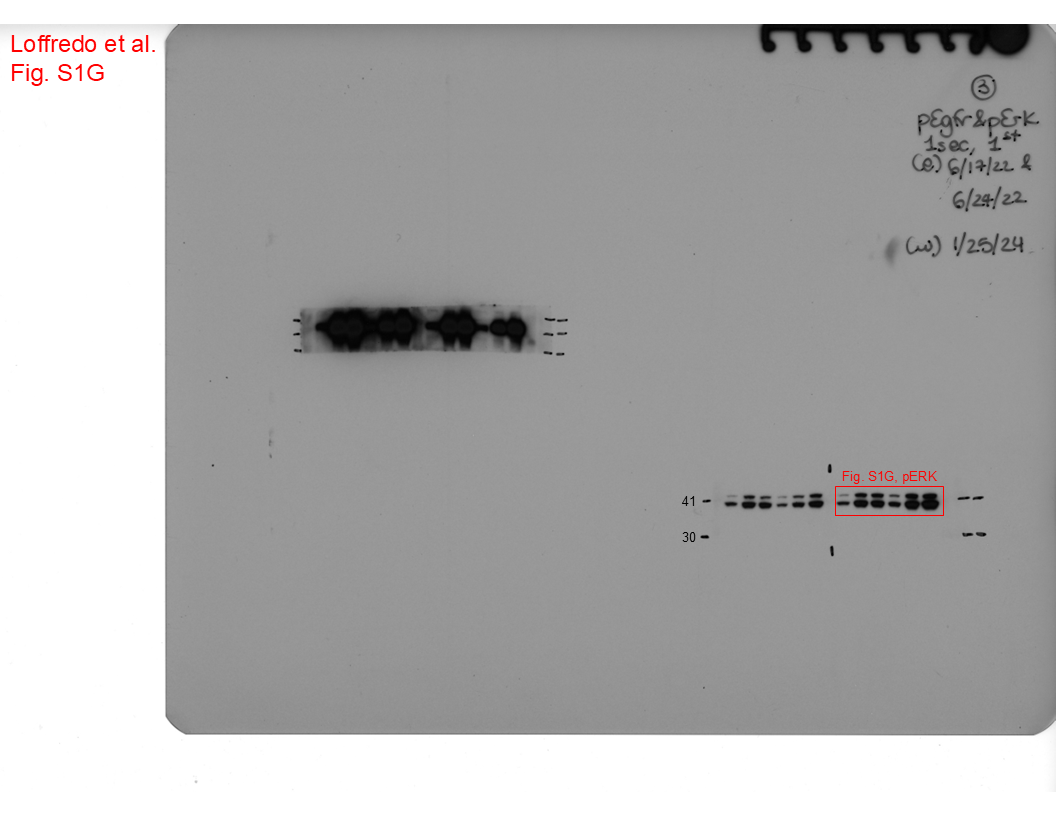

Supplement: Supplementary file 4 — Source Data [file 41467_2025_57362_MOESM4_ESM.zip › SourceData/Western_FigS1G_pERK.tif]
